# Efficacy and safety of a 3-day once-daily regimen of oral nafithromycin in comparison to oral moxifloxacin for the treatment of community-acquired bacterial pneumonia in adults: a phase III, randomized, double-blind controlled trial

**DOI:** 10.1016/j.lansea.2025.100666

**Published:** 2025-09-23

**Authors:** Himanshu Pophale, Monica Gupta, Lily Llorens, Piotr Iwanowski, Ranjeet Gutte, Rajesh Chavan, Anasuya Patel, Harsha Agrawal, Snehal Palwe, Prashant Joshi, Hariharan Periasamy, Mahesh Patel, Balaji Veeraraghavan, Sachin Bhagwat

**Affiliations:** a Jeevandhara Multispecialty Hospital, Pune, Maharashtra, India; b Samvedna Hospital, Varanasi, Uttar Pradesh, India; c Clinical Research, Wockhardt, Aurangabad (Chhatrapati Sambhajinagar), Maharashtra, India; d Discovery Research, Wockhardt, Aurangabad (Chhatrapati Sambhajinagar), Maharashtra, India; e Department of Clinical Microbiology, Christian Medical College, Vellore, Tamil Nadu, India

**Keywords:** Nafithromycin, Community-acquired bacterial pneumonia, Antibiotic resistance, Oral antibiotics, Fluoroquinolone, Ketolide, Macrolide

## Abstract

**Background:**

Nafithromycin, a novel macrolide belonging to the lactone ketolide subclass, exhibits excellent *in vitro* potency against pathogens causing community-acquired bacterial pneumonia (CABP), in conjunction with high and sustained pulmonary concentrations allowing for once-daily dosing. We aimed to compare efficacy and safety of nafithromycin with moxifloxacin for treatment of CABP.

**Methods:**

This was a phase III, randomized, double-blind, non-inferiority study in adults with CABP (PORT risk class II, III, IV), conducted at 31 sites across India. Patients were randomized (1:1) via block randomisation using interactive voice/web response system to receive oral nafithromycin 800 mg q24h for 3 days or oral moxifloxacin 400 mg q24h for 7 days. The primary efficacy endpoint was the proportion of patients with early clinical response (ECR) at Day 4 in the modified-intent-to-treat population (MITT). Favourable ECR was defined as alive and ≥1 level improvement in ≥2 CABP symptoms compared to baseline and without worsening of other CABP symptoms. A non-inferiority margin of 12.5% was utilised. This trial is registered with Clinical Trial Registry—India (CTRI/2019/11/021964).

**Findings:**

Between February 2021 and June 2023, 488 patients were enrolled with 244 randomized to each treatment. MITT population included 477 patients with 40% belonging to PORT risk class III/IV. Demography and baseline characteristics were comparable between groups. ECR was observed in 91.3% (220/241) of patients in nafithromycin group and 89.0% (210/236) of patients in moxifloxacin group of the MITT population [difference, 2.3%; 95% CI (−3.1, 7.8)] establishing statistical non-inferiority between treatments. Most common treatment-emergent adverse events reported (≥2% patients in any treatment group) were abdominal pain, diarrhoea, headache and nausea, which were all mild in severity.

**Interpretation:**

A 3-day regimen of oral nafithromycin was non-inferior to a 7-day regimen of oral moxifloxacin for the treatment of CABP.

**Funding:**

Co-funded by 10.13039/501100015512Wockhardt and 10.13039/501100014825BIRAC, Department of Biotechnology, Government of India.

**Research in context**

**Evidence before this study**

Community-acquired bacterial pneumonia (CABP) is a significant cause of morbidity and mortality in India, with an estimated 4 million cases per year. Furthermore, treatment failure with empirical therapy is not uncommon due to a multitude of reasons such as inadequate antimicrobial coverage, increased resistance, and patients with high-risk characteristics such as liver disease, renal impairment, immunocompromised or multilobar infiltrates. As it pertains to antimicrobial resistance, the most concerning is the emergence and increasing rates of macrolide resistance in *Streptococcus pneumoniae.* Disease management is further complicated by the challenge that determining the causative organism of CABP is not always possible from respiratory specimens in routine clinical practice making it difficult to optimise antibiotic therapy in a definitive manner. To assess the available evidences on the antibiotic treatment approach for CABP in India as well as globally, we searched PubMed for peer-reviewed papers from inception to March 31, 2025. The terms ‘community acquired bacterial pneumonia’, ‘community acquired pneumonia’, ‘clinical trials’, ‘treatment guidelines’ and ‘India’ were used in appropriate combinations. We also evaluated the treatment guidelines of Indian Council of Medical Research available online. Currently, guidelines and clinical practice for the management of CABP in India as well in other parts of the world with high macrolide resistance rates in *S. pneumoniae* include a β-lactam (±β-lactamase inhibitor) in combination with a macrolide to empirically cover both typical and atypical pathogens. While fluoroquinolones can be utilized, they are generally avoided due to concerns over adverse events—particularly in older patients—and, in India, the high prevalence of tuberculosis, which raises the risk of resistance selection and/or masking TB diagnosis. Thus, there is a clear need for clinically efficacious therapeutics that have a favourable safety profile, *in vitro* activity against clinically relevant mechanisms of resistance (β-lactam/macrolide-resistant *S. pneumoniae*), and an attractive short course dosing regimen (oral, once daily). To date, there has been no oral monotherapy regimen evaluated for the treatment of CABP as a 3-day course compared with a fluoroquinolone in a phase III clinical trial setting.

**Added value of this study**

To our knowledge, this is the first randomized trial evaluating an oral monotherapy option for the treatment of CABP as a 3-day treatment course. These pivotal data delineates the clinical utility and safety of nafithromycin for the treatment of CABP in a rigorous phase III study design. Demonstrating the efficacy of shorter courses of antibiotic therapy is not only offers compliance-benefit to the individual patient but also serves as a major advantage to help improve global antimicrobial resistance rates by reducing overall antibiotic consumption.

**Implications of all the available evidence**

In a setting where antimicrobial resistance is increasing and the development of new oral agents targeting community origin infections is limited, the availability of nafithromycin as a treatment option for CABP will help to fill a major unmet need.

## Introduction

Nafithromycin (WCK 4873), is a novel oral macrolide belonging to the lactone ketolide subclass with *in vitro* activity against clinically important typical and atypical respiratory pathogens such as *Streptococcus* *pneumoniae*, *Staphylococcus aureus*, Group A streptococci, *Haemophilus influenzae*, *Moraxella catarrhalis, Chlamydophila pneumoniae*, *Mycoplasma pneumoniae*, and *Legionella pneumophila*. Importantly, the optimized structure of nafithromycin allows it to overcome multiple mechanisms of macrolide resistance, such as ermB-mediated ribosomal modifications and *mef* (A/E)-mediated efflux pumps.^1^, ^2^, ^3^ While global prevalence of these mechanisms of resistance can vary, both of these are responsible for macrolide resistance in *S. pneumoniae* isolates.^4^ Based on recent *in vitro* susceptibility data, nafithromycin has the ability to overcome these mechanisms of resistance, as demonstrated in multiple studies.^4^, ^5^, ^6^, ^7^ Against *S. pneumoniae* including those resistant to macrolides, nafithromycin showed potent *in vitro* activity with MIC_90_ ranging from 0.06 to 0.25 mg/L in different studies conducted by independent investigators located in the US, China and India. As a part of a global SENTRY surveillance program (2016), nafithromycin was evaluated against 1911 *S. pneumonia*e isolates collected across different geographical regions such as the US, Europe, Latin America and Asia, wherein it demonstrated MIC_90_ ranging from 0.06 to 0.12 mg/L.^4^, ^5^, ^6^ Nafithromycin has also demonstrated good activity against key atypical pathogens like *M. pneumoniae*, *Chlamydia pneumoniae* and *L. pneumophila*.^7^, ^8^, ^9^

In addition to its *in vitro* activity against relevant respiratory pathogens, there are multiple attributes of nafithromycin that make it a suitable agent for the treatment of community-acquired bacterial pneumonia (CABP). Preclinical *in vivo* murine lung infection models have also demonstrated efficacy against *S. pneumoniae* and *S. aureus*.^10^, ^11^, ^12^, ^13^, ^14^ *In* vitro studies have demonstrated a meaningful post-antibiotic effect of ≥5 h against drug-resistant strains, providing additional support to the rationale of once-daily dosing when combined with its pharmacokinetic profile.^15^ Similar to other macrolides, nafithromycin has also been shown to have immunomodulatory effects.^16^ Finally, nafithromycin was also found to have bactericidal activity when evaluated in an intracellular infection model against *L. pneumophila*. Bactericidal activity of nafithromycin has also been established against *S. pneumoniae* in a time-kill study at clinically relevant concentrations.^7^^,^^17^ These data, coupled with the high intracellular concentrations observed in human alveolar macrophages (AM) further corroborate the use of nafithromycin against atypical respiratory pathogens.^18^

Pharmacokinetic studies have demonstrated a favourable pharmacokinetic profile that allows for once-daily dosing with excellent systemic exposures through oral route.^18^^,^^19^ Specifically, high lung penetration ratios (mean ELF/unbound plasma AUC_0-24_ of 69 and mean AM/unbound plasma AUC_0-24_ of 2635) have been demonstrated and are supportive of therapeutic efficacy based on susceptibilities of target pathogens.^18^ Also, therapeutically relevant concentrations of nafithromycin in the ELF and AM were sustained for up to 48 h after the 3rd dose in healthy participant studies.^18^ These features of nafithromycin collectively make it a promising agent for the treatment of CABP. Herein, we report the findings of the phase III study evaluating a 3-day oral once-daily regimen of nafithromycin compared to a 7-day oral once-daily regimen of moxifloxacin.

## Methods

### Study design and participants

This was a phase III, multi-center, double-blind, randomized study conducted at 31 sites throughout India between February 2021 and June 2023. The study was designed to evaluate oral nafithromycin 800 mg every 24 h for 3 days versus oral moxifloxacin 400 mg every 24 h for 7 days in adults with CABP. The clinical trial protocol is provided in Supplement 1.

The trial was conducted in accordance with Good Clinical Practice by the International Council for Harmonisation (ICH-GCP) guidelines and New Drugs and Clinical Trial 2019 rules. The study protocol and other appropriate study related information was approved by the Institutional Review Board (IRB) or ethics committee at each participating site prior to enrolment of study subjects. The names and approval reference number are the following; Shivam Ethics Committee (SEC/03/20/003), Shree Giriraj Hospital Research Ethics Committee (GH-FR-05, 00/21-03-2013), Institutional Ethics Committee, N.K.P. Salve Institute of Medical Sciences, and Lata Mangeshkar Hospital (ECR/88/Inst/MH/2013/RR-16, 4-OCT-2019), Eternal Heart Care Centre & Research Institute, Institutional Ethics Committee (018/2023), Samvedna Hospital Ethics Committee (ECR/45/Inst/UP/2013/RR-16, 28-JULY-2020), Ethics Committee, Unique Hospital (ECR/595/Inst/GJ/2014/RR-20, 6-AUG-2019), Institutional Ethics Committee Mar Augustine Golden Jubilee Hospital (ECR/402/Inst/KL/2013/RR-19, 24-JAN-2020), Ethics Committee of Bangalore Medical College & Research Institute (ECR/302/Inst/KA/2013/RR-16, 5-OCT-2021), Institutional Ethics Committee, JSS Medical College (JSSMC/IEC/120819/05/CT Final Approval/20-21), Sanjivani Hospital Ethics Committee (ECR/183/Inst/Ahm/2013/RR-19, 17-AUG-2019), Institutional Human Ethics Committee GMERS Medical College and Hospital (IHEC: 260/2019), Scientific Research and Ethical Review Committee (SRERC/2019/25/22), Institutional Ethics Committee, Mysore Medical College and Research Institute (MMC EC 148/21), OMEGA Ethics Committee (ECR/89/Inst/KA/2013/RR-20), Ethics Committee Vinaya Hospital (ECR/664/Inst/KA/2014/RR-17, 20-AUG-2019), Institutional Ethics Committee for Human Research (IECHR) Medical College & SSG Hospital Baroda (ECR/85/Inst/GJ/2013/RR-19, 8-Nov-2019), Institutional Ethics Committee Topiwala National College and BYL Nair Charitable Hospital (IEC/48/2020), Institutional Ethics Committee Paras Hospital (ECR/249/Inst/Har/2013/RR-16, 31-JULY-2020), Institutional Ethics Committee Govt Medical College (Pharma/IEC-GMCA/380/2019), Ethics Committee GSVM Medical College, Kanpur (CT/14/EC/Feb/2020), Institutional Ethics Committee Midland Healthcare & Research Center (IEC/12th/2020), Marudhar Hospital Ethics Committee (MHEC/2019/09/02), Institutional Ethics Committee Sai Sneh Hospital & Diagnostic Centre (IECSSH061021, IECSSH061121, IECSSH110722, IECSSH180722), Shree Hospital Ethics Committee (CR/553/Inst/MH/2014/RR-20, 18-JAN-2022), Siddhi Hospital Institutional Ethics Committee (ECR/739/Inst/MH/2015/RR-21), Institutional Ethics Committee Dr M K Shah Medical College & Research Centre & SMS Multispecialty Hospital (MKMSMCRC/IEC/2213-0901), Saikrupa Hospital Institutional Ethics Committee (SHIEC/013), and Viveka Hospital Ethics Committee (EC/001/2012). Voluntary written informed consent was obtained prior to subject enrolment in the study. The method used to collect sex/gender data was self-reported with the options being male or female.

Key inclusion criteria included adults (≥18 years of age) with PORT risk class II, III, or IV (PORT score of 51–105). In addition, at least 2 new or worsening symptoms of CABP (dyspnea, cough, purulent sputum or pleuritic chest pain); vital sign abnormalities (at least two of the following: fever or hypothermia, hypotension, tachycardia or tachypnoea); clinical signs or laboratory abnormalities (at least one of the following: hypoxaemia, auscultatory finding consistent with bacterial pneumonia or pulmonary consolidation, elevated white blood count (WBC) or leukopenia, or elevated immature neutrophils); and radiographic evidence of CABP. Key exclusion criteria included receipt of 1 or more doses of a potentially effective systemic antibacterial for the treatment of CABP within 72 h before randomisation except if the prior therapy was a single dose of a short-acting antibacterial, other types of pneumonia (i.e. hospital-acquired bacterial pneumonia, ventilator-associated bacterial pneumonia), pleural empyema, non-infectious cases of pulmonary infiltrates, evidence of significant immunologic disease, compromised hepatic or renal function, active or suspected pulmonary tuberculosis. Patients with clinical conditions necessitating in-patient treatment were excluded. A complete list of inclusion and exclusion criteria are provided in Supplement 1. Enrolment of PORT risk class II patients was capped at 60%. In addition, enrolment of subjects who received prior antibiotic therapy was capped at 25%. The stratification was only done by PORT Risk class and not for the investigational sites.

### Randomisation and study intervention

Patients were randomized 1:1 in a blinded manner to receive nafithromycin 800 mg (two-400 mg tablets) PO q24h for 3 days or moxifloxacin 400 mg (one capsule: over-encapsulated tablet) PO q24h for 7 days. Block randomisation (block size of four) was performed using interactive voice/web response system (IXRS). The IXRS system also confirmed the study drug assignment including the unique identification number(s) of the kits to be dispensed to the subject. The pharmacist was responsible for maintaining accountability and dispensing the oral study drugs according to the handling instructions.

To maintain the study blind, the study medication was packaged in kits containing the daily dose of tablets/capsules and matching placebo tablets/capsules, to be taken each day for the duration of treatment according to the randomized treatment allocation. Patients received either a nafithromycin or a moxifloxacin kit, while kits were identified only by a “kit number” to conceal the specific treatment allocation. Patients were instructed to take two tablets and one capsule daily for 7 days and to bring the kits to each study visit for accountability and compliance. Thus, the patients in nafithromycin group received 2 tablets of nafithromycin 400 mg and one placebo capsule per day during Day 1–3. From Day 4 to Day 7, patients received 2 placebo tablets and one placebo capsule per day in nafithromycin group. In the moxifloxacin group, from Day 1 to Day 7, patients received one moxifloxacin 400 mg capsule and two placebo tablets. All personnel at the study centres including the investigators as well as statistician, data managers, contract research organisation and sponsor remained blinded until the study enrolment was completed and database was locked. Randomisation was stratified by PORT risk class II or III/IV. Study drugs were administered approximately at the same time every day through the treatment period. The randomisation and subsequent treatment initiation was ‘empirical’ as this is the standard of care for CABP. Patients were to be treated in the study as outpatients, however inpatients were allowed for convenience or social purposes at the discretion of the investigator. Decisions to continue or discontinue study drug was based on clinical condition of the patient.

### Analysis populations

The intent-to-treat (ITT) population included patients who were randomized, regardless of whether they received the study drug. The modified-intent-to-treat (MITT) population included the ITT population who received at least one dose of the study drug and tested negative for *Mycobacterium tuberculosis* complex (MTBC) based on the results of the GeneXpert TB test. Considering the possibility of overlapping clinical symptoms of CABP and pulmonary tuberculosis, patients with suspected or confirmed tuberculosis have been excluded from the MITT population and considered as primary analysis population. These patients were analysed as per the randomized treatment groups. The microbiological MITT (mMITT) population included patients who received at least one dose of study drug and had at least 1 baseline pathogen known to cause CABP against which the investigational drug has antibacterial activity (e.g., *S. pneumoniae, S. aureus, H. influenzae, H. parainfluenzae, M. catarrhalis, L. pneumophila, M. pneumoniae, C. pneumoniae)*. The clinically evaluable (CE) population included all patients in the MITT population who met key inclusion/exclusion criteria, test of cure (TOC) visit occurred within 11–21 days of randomisation (unless patient was deemed a failure before this visit), did not receive a non-study antibacterial with potential efficacy against the baseline pathogen, did not have indeterminate outcome at TOC, received at least 80% of intended doses of study drug therapy, received at least 48 h (equivalent to 2 or more days, or at least 2 doses, given the once-daily dosing schedule) or 72 h (equivalent to 3 or more days, or at least 3 doses) of study drug to be considered an evaluable clinical failure or success, respectively, and did not have any significant protocol violations that may confound efficacy assessment at TOC. Even though the protocol-specified window for the TOC visit was 15 ± 4 (11–19) days from the study start, the window was widened to 11–21 days so that TOC visit was not missed because of social reasons. For the ITT, MITT, mMITT, and CE populations, analyses were performed based on the randomized treatment groups. The safety population included all patients who received any amount of study drug and the analyses were performed according to the treatment actually received.

### Endpoints/outcomes

On Day 4, counted from the first day of dosing, patients in the MITT population of both treatment groups were assessed for early clinical response (ECR) and categorized as favourable, unfavourable, or indeterminate. The historical evidence for the benefit of antimicrobial therapy in the treatment of CABP is based on CABP symptoms, evaluated after 3–5 days of therapy, irrespective of the duration of therapy. Therefore, an evidence-based non-inferiority margin for a symptom-based clinical endpoint could only be justified for the early clinical response (assessed on Day 4 in this study).^20^ A favourable ECR (programmatically determined; computer algorithm, written to match the protocol definition) was defined as alive and at least 1 level improvement (e.g., severe to moderate, moderate to mild, mild to absent) in at least 2 CABP symptoms (dyspnea, cough, production of purulent sputum or pleuritic chest pain) compared with the baseline visit and without worsening in any other CABP symptoms. The severity of symptoms were assessed by a 4-point scale by the investigator based on the CABP Symptom Severity Guidance for Investigator Assessment (Appendix III of study protocol, Supplement 1). Unfavourable response was defined as no improvement in at least 2 CABP symptoms compared with that at baseline, or worsening in any of the CABP symptoms compared with that at baseline, or death from any cause at or before Day 4. Patients with missing data or those lost to follow-up (FU) were classified as having an indeterminate response. The primary efficacy endpoint was the proportion of patients in the MITT population in each treatment group, who demonstrated a favourable ECR on Day 4. Patients with an indeterminate response were included in the denominator and considered as having an unfavourable response for this calculation; thus, no patients were excluded from the analysis. Study endpoints were in accordance to US Food and Drug Administration (FDA) CABP guidance and accepted by the Drugs Controller General of India (DCGI).^20^

Secondary endpoints include investigator-determined clinical outcomes at EOT (Day 7 + 2 days) and TOC (Day 15 ± 4) visits. For both of these endpoints, clinical cure was defined as being alive and symptoms of CABP being resolved or returned to premorbid conditions with improvement in cough such that antibacterial therapy was not needed. Clinical failure was defined as signs and symptoms of CABP did not resolve or return to premorbid conditions and/or worsened cough such that additional antibacterial therapy was initiated or death prior to assessment. Patients with missing data or those lost to follow up were defined as indeterminate. Subgroup analyses of ECR at Day 4 was also conducted. These included summaries by age, gender, BMI, markers of CABP, PORT risk class and renal function. Investigator-determined clinical cure rates at TOC by baseline pathogen and by infecting organism type in the mMITT population were also determined.

Safety data, including treatment emergent adverse events (TEAE) were summarized for the safety analyses. TEAE was defined as an adverse event occurring or worsening on or after the administration of the first dose of study drug. In addition to adverse events, vital signs, laboratory evaluations (hematology, chemistry panel, urine analysis) and ECG parameters were evaluated.

### Statistical analysis

All analyses were conducted in accordance with the study protocol. For the primary efficacy endpoint, the proportion of patients in the MITT analyses set with a favourable ECR at Day 4 was calculated for each treatment group. The ‘treatment difference’ (nafithromycin minus moxifloxacin) was derived as the difference in these proportions between the groups. A two-sided 95% confidence interval (CI) for the treatment difference was determined using the method of Miettinen and Nurminen.^21^ Non-inferiority was concluded if the lower limit of the two-sided 95% CI was greater than −12.5%.

For subgroup analyses, the number and percentage of patients with a favourable clinical response at Day 4 were summarized by treatment group, and the treatment difference, along with its two-sided 95% CI, was similarly estimated using the Miettinen and Nurminen method. Secondary endpoints were analysed by summarizing the number and percentage of patients in each outcome category for each treatment group. The treatment difference in proportions, along with the corresponding two-sided 95% CI, was also calculated using the Miettinen and Nurminen method. The non-inferiority hypothesis was tested only for the primary efficacy endpoint. All analyses related to secondary endpoints and subgroup comparisons were considered descriptive.

To determine the study sample size, a favourable ECR rate at Day 4 of 85%–90% was projected with an anticipated dropout rate by Day 4 of at most 5%. Since patients who dropped out prior to Day 4 were included in the denominator for the proportion of patients with favourable ECR at Day 4, a favourable ECR rate at Day 4 of 81%–86% was anticipated in each treatment group. Based on these rates, a non-inferiority margin of 12.5% and using the Farrington-Manning sample size approach for the Miettinen and Nurminen method, it was determined that approximately 414 patients would need to be in the MITT population in order to meet 90% power at the 1-sided 2.5% significance level. Assuming a tuberculosis positivity rate of 15%, a sample size of 488 patients would be needed in the MITT population. Statistical analyses were performed using SAS Software, Version 9.4 (SAS Institute Inc., Cary, NC, USA).

### Sensitivity analysis

Two sensitivity analyses for the primary efficacy endpoint were pre-specified in the statistical analysis plan (SAP, provided in Supplement 2). A comparison of ECR rates at Day 4 between treatment groups was conducted within the MITT analysis set using the Miettinen-Nurminen method, stratified by the randomisation factor: PORT Risk Class. The stratification included classes II (eligible) and III/IV (eligible: score 91–105; ineligible: score 106–130). Classes I and V were considered ineligible for the study and thus excluded from the stratified analysis.

In the primary analysis, subjects with missing data were classified as having an indeterminate response. These subjects were included in the denominator and considered to have an unfavourable outcome. A sensitivity analysis was also performed in which subjects with an indeterminate response in the MITT analysis set were imputed as having a favourable outcome. This analysis employed the same statistical approach as the primary analysis using the Miettinen-Nurminen method.

### Role of funding source

This study was co-funded by Wockhardt and Biotechnology Industry Research Assistance Council (BIRAC), India. Wockhardt was the sponsor of the study, responsible for developing the study protocol, study conduct and publishing. The data management was undertaken by a contract research organisation. Authors had full and independent access to all study data and accepted responsibility to submit for publication. The authors were not paid to write this article.

The registration number for the study is CTRI/2019/11/021964 and the registration date is 11/11/2019. The URL for the clinical trial registration page: https://ctri.nic.in/Clinicaltrials/advsearch.php.

## Results

**Fig. 1:**
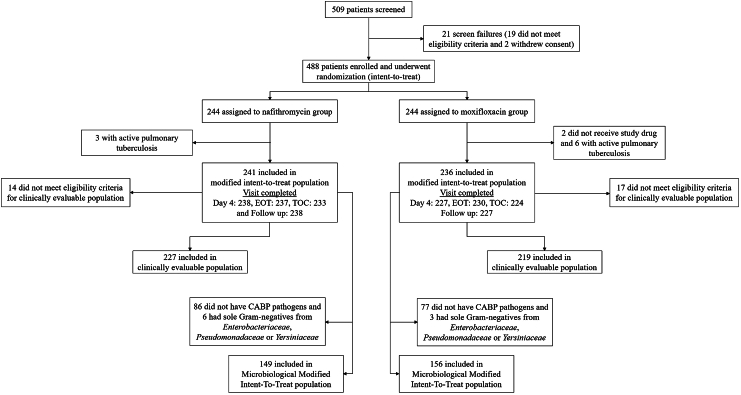
**Patient disposition and analyses populations**.

A total of 488 patients were enrolled from February 10, 2021 to June 12, 2023 in the study with 244 randomized to each treatment group (Fig. 1). Of these, 477/488 (97.8%) were included in the MITT population and 486/488 (99.6%) in the safety population. In the MITT population, 465/477 (97.5%) of patients completed the study through the FU visit. 12 patients (2.5%) did not complete study drug due to withdrawal of consent or adverse event (Fig. 1).

Demographics and baseline characteristics were comparable between treatment groups in the MITT population (Table 1). The majority of patients were <65 years old (89.3%) and male (80.9%). The mean weight and height were 63.79 kg and 163.97 cm, respectively. 60% of patients were PORT risk class II which met the protocol defined cutoff. While all the patients were screened, no patients in either treatment group were identified as concurrently bacteremic. One dose of short acting antibiotic was administered to approximately 7% of patients in either of the treatment group within 72 h of randomisation. These short-acting antibiotics were amoxicillin, cefixime, cefpodoxime, doxycycline and erythromycin. The majority of the patients (413, 86.6%) had fever (>38 °C orally or > 38.5 °C rectally or tympanically) [nafithromycin: 214/413 (88.8%); moxifloxacin 199/413 (84.3%)]. Demographics and baseline characteristics were also similar between treatment groups in the safety population (data not shown).

**Table 1:** Demographics and baseline characteristics (MITT population).

Demographics	Nafithromycin (N = 241)	Moxifloxacin (N = 236)	Overall (N = 477)
**Age (years)**			
Mean (SD)	40.56 (13.92)	42.66 (15.16)	41.60 (14.57)
Median (min–max)	38 (19.0, 76.0)	38 (18.0, 87.0)	38 (18.0, 87.0)
**Age category, n (%)**			
<65 yrs	220 (91.3)	206 (87.3)	426 (89.3)
≥65 yrs	21 (8.7)	30 (12.7)	51 (10.7)
**Gender, n (%)**			
Male	196 (81.3)	190 (80.5)	386 (80.9)
Female	45 (18.7)	46 (19.5)	91 (19.1)
**Race, n (%)**			
Indian (From Indian Subcontinent)	241 (100.0)	236 (100.0)	477 (100.0)
**Ethnic origin, n (%)**			
Not Hispanic or Latino	241 (100.0)	236 (100.0)	477 (100.0)
**Geographic region, n (%)**			
Indian Subcontinent	241 (100.0)	236 (100.0)	477 (100.0)
**Weight (kg)**			
Mean (SD)	63.36 (10.71)	64.23 (11.59)	63.79 (11.15)
Median (min–max)	64.0 (32.5, 99.0)	64.9 (36.8, 111.9)	64.2 (32.5, 111.9)
**Body mass index (kg/m^2^)**			
Mean (SD)	23.60 (3.91)	23.89 (4.11)	23.75 (4.01)
Median (min–max)	23.4 (13.7, 37.7)	23.5 (13.7, 44.8)	23.4 (13.7, 44.8)
**Body mass index (kg/m^2^) category, n (%)**			
<18.5 (underweight)	23 (9.5)	14 (5.9)	37 (7.8)
18.5–< 25 (normal)	128 (53.1)	133 (56.4)	261 (54.7)
≥25–< 30 (overweight)	78 (32.0)	74 (30.6)	152 (31.3)
≥30–< 35 (obese)	10 (4.1)	15 (6.4)	25 (5.2)
≥35 (morbidly obese)	3 (1.2)	2 (0.8)	5 (1.0)
**Medical history and co-morbidities, n (%)**			
Diabetes	8 (3.3)	8 (3.3)	16 (3.3)
Hypertension	8 (3.3)	15 (6.2)	23 (4.7)
**Disease Severity, n (%)**			
PORT risk class II	146 (60.6)	140 (59.3)	286 (60.0)
PORT risk class III/IV	95 (39.4)	96 (40.7)	191 (40.0)
Bilateral involvement	75 (31.1)	90 (38.1)	165 (34.6)
**Vital signs, mean (SD)**			
Systolic Blood Pressure (mmHg)	124.3 (11.2)	125.6 (9.1)	125.0 (10.2)
Diastolic Blood Pressure (mmHg)	81.8 (8.4)	80.8 (7.0)	81.3 (7.8)
Heart Rate (beats/min)	99.7 (18.3)	95.7 (18.4)	97.7 (18.5)
Respiratory rate (breaths per minute)	29.8 (5.9)	29.2 (5.3)	29.5 (5.6)
**Creatinine clearance (mL/min), n (%)**			
<30	0 (0.0)	0 (0.0)	0 (0.0)
30–60	21 (8.7)	22 (9.3)	43 (9.0)
>60–90	58 (24.1)	63 (26.7)	121 (25.4)
>90	162 (67.2)	151 (64.0)	313 (65.6)
**Systemic inflammatory response syndrome, n (%)**	231 (95.9)	218 (92.4)	449 (94.1)
**Previous antibiotic usage, n (%)**	17 (7.1)	15 (6.4)	32 (6.7)

**Fig. 2:**
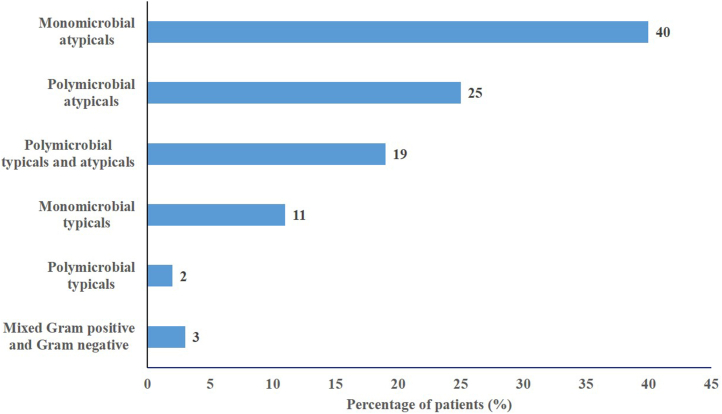
**Distribution of baseline pathogens by infecting organism type in mMITT population (n = 305)**.

A baseline pathogen was identified in 305 patients (mMITT population). A qualifying typical pathogen was identified in 107/305 (35.1%) of mMITT population [nafithromycin: 51/149 (34.2%); moxifloxacin 56/156 (35.9%)]. The most common typical pathogens in mMITT analyses were *S. pneumoniae* [nafithromycin: 7/149 (4.7%); moxifloxacin 12/156 (7.7%)]*, H. influenzae* [nafithromycin: 15/149 (10.1%); moxifloxacin 11/156 (7.1%)]*, M. catarrhalis* [nafithromycin: 21/149 (14.1%); moxifloxacin 13/156 (8.3%)]*,* and *H. parainfluenzae* [nafithromycin: 13/149 (8.7%); moxifloxacin 11/156 (7.1%)]. The most common atypical pathogen was *C. pneumoniae* which was identified in 64.3% of patients [nafithromycin: 95/149 (63.8%); moxifloxacin 101/156 (64.7%)]. *M. pneumoniae* and *L. pneumophila* were identified in 29.2% and 31.5% of subjects, respectively, with similar frequency between the two treatment groups. The distribution of monomicrobial and polymicrobial infections in the mMITT population are shown in Fig. 2. The MICs of nafithromycin for baseline typical pathogens ranged 0.06–128 mg/L for *S. aureus*, 0.002–0.12 mg/L for *S. pneumoniae* (including macrolide-resistant phenotype), 1–16 mg/L for *H. influenzae*, and 0.03–1 mg/L for *M. catarrhalis*.

An early clinical response (ECR) at Day 4 was observed in 91.3% (220/241) of patients in the nafithromycin group and 89.0% (210/236) of patients in the moxifloxacin group of the MITT population [difference, 2.3 95% CI (−3.1, 7.8)] establishing non-inferiority of nafithromycin compared with moxifloxacin (Table 2). Unfavourable clinical response rates were in 18/241 (7.5%) in the nafithromycin group and 17/236 (7.2%) in the moxifloxacin group. Patients with missing data or lost to follow up were classified as indeterminate [nafithromycin (3/241, 1.2%), moxifloxacin (9/236, 3.8%)].

**Table 2:** Favourable early clinical response (ECR) and investigator-determined clinical cure rate (IDCCR) in MITT, mMITT and CE populations.

Non-est = non-estimabl.

In one of the sensitivity analyses, using the PORT risk class stratification factor, a treatment difference of 0.01 with corresponding 95% confidence interval of −0.055 to 0.078 was obtained for the PORT risk class II and 0.041 with corresponding 95% confidence interval of −0.056 to 0.139 for the PORT risk class III/IV. In the other sensitivity analysis, 3/241 (1.2%) of patients in the nafithromycin treatment group and 9/236 (3.8%) in the moxifloxacin treatment group were imputed as favourable ECR. The favourable ECR was 92.5% (223/241) in nafithromycin treatment group and 92.8% (219/236) in the moxifloxacin treatment group with corresponding treatment difference of −0.003 (95% CI: −0.051, 0.046). Thus, the preplanned sensitivity analyses continued to support non-inferiority of nafithromycin compared to moxifloxacin.

ECR rates observed in the mMITT and CE populations were consistent with the rates observed in the primary study population. Investigator-determined clinical cure rates (IDCCR) at EOT and TOC were similar between treatment groups in all study populations and were relatively high.

**Fig. 3:**
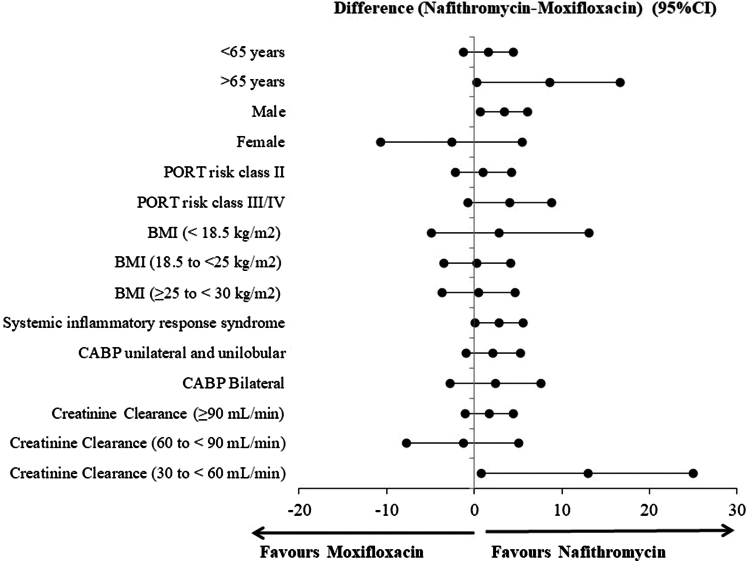
**Treatment difference in the favourable clinical response at day 4 by demographics, baseline characteristics and CABP markers (MITT population**).

While this study was not designed for statistical inferences in subgroups, clinical response at Day 4 by demographics, baseline characteristics and markers of CABP were similar between both treatment groups (Fig. 3). The key subgroup analysis demonstrated consistent efficacy of nafithromycin regardless of age, sex, PORT risk class, BMI, extent of pulmonary involvement, systemic inflammatory response, and renal function categories (mild and moderate) as shown in Fig. 3. Overall, IDCCR at TOC between treatment groups was similar irrespective of the patient had a monomicrobial or polymicrobial infection. Note the distribution of monomicrobial and polymicrobial infections in the study were also similar. Atypical pathogens were the most common baseline organism isolated in the study. IDCCR rates at TOC for all atypical organisms were 126/129 (97.7%) compared to 130/134 (97.0%) for nafithromycin and moxifloxacin, respectively. Gram-negative fastidious pathogens were the most common typical organism in both treatment groups at baseline. IDCCR at TOC for these pathogens were similar between treatment groups at 42/44 (95.5%) compared to 32/36 (88.9%), for nafithromycin and moxifloxacin, respectively.

The overall incidence of TEAEs was 20.1% with nafithromycin and 15.3% with moxifloxacin (Table 3). Most TEAEs were mild in severity, less than 2% were classified as moderate and there were no severe TEAEs. The moderate TEAEs were comparable between both the treatment groups. There were no deaths or serious TEAEs. Discontinuation of study drug occurred in 2 patients in the moxifloxacin group due to vomiting, pruritus, and rash. There was no discontinuation of study drug in the nafithromycin group due to TEAEs.

**Table 3:** Overall summary of treatment-emergent adverse events (Safety population).

Patient category	Nafithromycin (N = 244)	Moxifloxacin (N = 242)
Total number of TEAEs	70	51
Number of study treatment related TEAEs	17	14
Patients with any TEAE, n (%)	49 (20.1)	37 (15.3)
Patients with treatment related TEAE, n (%)	11 (4.5)	7 (2.9)
**TEAE by maximum severity, n (%)**		
Mild	48 (19.7)	34 (14.0)
Moderate	4 (1.6)	3 (1.2)
Severe	0 (0)	0 (0)
Serious TEAE, n (%)	0 (0)	0 (0)
TEAE leading to drug discontinuation, n (%)	0 (0)	2 (0.8)
TEAE leading to death, n (%)	0 (0)	0 (0)

TEAEs in ≥2% of patients in either group, n (%)				
System Organ Class (SOC) Preferred Term (PT)	Nafithromycin (N = 244)		Moxifloxacin (N = 242)	
	Total	Related	Total	Related
**Gastrointestinal disorders**				
Abdominal pain upper	9 (3.7)	0 (0)	7 (2.9)	0 (0)
Diarrhoea	5 (2.0)	1 (0.4)	10 (4.1)	2 (0.8)
Nausea	7 (2.9)	6 (2.5)	7 (2.9)	4 (1.7)
**Nervous system disorders**				
Headache	15 (6.1)	0 (0)	6 (2.5)	0 (0)

The most common TEAEs reported by patients (≥2%) were abdominal pain, diarrhea, headache and nausea, which were all mild in severity. The TEAEs of headache and abdominal pain in both the treatment groups was unrelated to the study drug as assessed by the investigator. Potentially clinically significant (PCS) changes in hematology, chemistry, including liver parameters were also analysed. With respect to changes in hematology and chemistry parameters, 8.2% (20/244) and 8.2% (20/244) in the nafithromycin group and 7.4% (18/242) and 6.6% (16/242) in the moxifloxacin group, respectively, had at least one PCS change. Overall, there were no clinically meaningful differences between treatment groups in hematology, chemistry, coagulation, vital signs and ECG results. Of note, there were 3 patients in the moxifloxacin group with potentially clinically significant change in QTcF interval (QTcF interval of >500 msec and >60 msec rise from baseline) compared to none in nafithromycin group. These observations in the moxifloxacin treatment group are in line with the safety profile of fluoroquinolone antibiotics, including moxifloxacin, known for prolongation of QT interval.

For changes in liver parameters, post baseline elevations ≥3× upper limit of normal for ALT or AST or >2× upper limit of normal for total bilirubin were infrequent and comparable between treatment groups. In both treatment groups, liver enzymes returned to baseline or near baseline values at the TOC visit. No patients in either treatment group met criteria for Hy's law.

## Discussion

This Phase III trial demonstrated non-inferiority of a 3-day course of oral nafithromycin versus a 7-day course of oral moxifloxacin in adult patients with CABP based on an ECR rate of 91% for nafithromycin and 89% for moxifloxacin in the MITT population. In addition to typical CABP pathogens, atypical CABP pathogens were frequent at baseline, a trend common in India among CABP patients. These results were consistent across subgroups analysed as well, such as age, gender, BMI, markers of CABP, PORT risk class, renal function and baseline pathogens relevant for CABP, such as *H. influenzae, H. parainfluenzae, M. catarrhalis, C. pneumoniae, L. pneumophila,* and *M. pneumoniae.* Investigator-determined clinical cure rates at EOT and TOC were also high and comparable between treatment groups. Overall, both agents were safe and well tolerated, with no new safety signals identified and no severe TEAE reported.

Historically, the standard duration of treatment in many CABP trials was 7–14 days which over the past 2 decades many experts have raised the question if these longer durations are warranted.^22^ After further review, durations in many CABP trials were shortened to 5–7 days and the efficacy is well supported by clinical evidence.^23^, ^24^, ^25^, ^26^ While antibiotics are generally considered safe, reducing the overall exposure is associated with many benefits, particularly as it relates to antimicrobial resistance rates, cost, and development of *C. difficile* infections or intestinal dysbiosis. To change clinical practice and prescribing patterns, it is generally accepted that well designed randomized controlled trials are needed.^27^ This makes the current study establishing non-inferiority of nafithromycin as a 3-day regimen, unique and progressive. Prior to the initiation of this Phase III study, a global Phase II study was performed to help inform the nafithromycin dosing regimen.^28^ The following treatment arms were evaluated: 1) nafithromycin 800 mg orally once daily for 3 days, 2) nafithromycin 800 mg orally once daily for 5 days, and 3) moxifloxacin 400 mg orally once daily for 7 days. Overall clinical cure rates in both nafithromycin treatment arms were high and comparable to the moxifloxacin arm, therefore the 3-day regimen was selected for the current Phase III study. A 3-day therapy of nafithromycin was efficacious owing to high and sustained lung ELF concentrations in conjunction with high potency and bactericidal action. Moreover, in both Phase II and Phase III studies, efficacy rates observed at early assessment visit sustained at EOT and TOC.

Moxifloxacin has been used as a comparator agent in multiple CABP trials and has consistently demonstrated high clinical cure rates.^29^, ^30^, ^31^ Moreover, it is active against both typical and atypical respiratory pathogens. It is also currently recommended as one of the respiratory fluoroquinolones for the treatment of CABP by both American Thoracic Society and IDSA guidelines.^23^ While it is an appropriate comparator for this clinical trial, the class of fluoroquinolones is associated with several safety concerns such as tendon rupture, peripheral neuropathy, aortic aneurysm and dissection, impact on blood glucose, and CNS effects.^32^ This further illustrates the need for new and efficacious treatment options for CABP, particularly in different drug classes that can be utilised in patients at higher risk of developing these adverse effects.

Strengths of this study include the evaluation of an oral short course (3-day) of nafithromycin in a robust clinical trial design against a highly effective comparator. Overall, the study had very low dropout or treatment discontinuation rates. In addition to standard respiratory specimen culture workup, urine antigen and serology were also utilised which allowed for additional baseline pathogen detections and an ample mMITT study population. At the same time, non-culture-based methods do not allow for MIC testing, thereby limiting the amount of susceptibility data. Lastly, the entire study population was Indian limiting the racial diversity, however a previously conducted Phase II study was multinational (Europe, U.S. and South Africa) with diverse ethnicity. Notably, despite differences in the ethnicity of CABP patients between Phase II and Phase III trials, the efficacy and safety outcomes of both nafithromycin and moxifloxacin were comparable, suggesting generalizability of the outcome of this study.

This study was designed to robustly and reliably evaluate the efficacy and safety of nafithromycin in CABP patients often treated in out-patient settings. Therefore, subjects with serious co-morbidities or life expectancy of less than 2 months were not eligible corroborating absence of mortality in this study. Despite most enrolled patients being younger, a high proportion of patients with PORT III and IV could be enrolled as they manifested more severe symptoms of disease although not at imminent risk of a fatal outcome. It is accepted that higher PORT score is a function of age, co-morbidities and severity of infection.

In conclusion, a 3-day course of oral nafithromycin was non-inferior to a 7-day course of oral moxifloxacin for the treatment of CABP in adults. Nafithromycin has excellent *in vitro* activity against relevant typical and atypical respiratory pathogens. These data provide strong evidence for the therapeutic utility of nafithromycin in CABP as a safe and efficacious monotherapy option, particularly in the setting of increased macrolide resistance.

## Contributors

HP (Himanshu Pophale) and MG were the study investigators responsible for the conduct of the trial, data acquisition and interpretation of the results. LL was involved in preparation of statistical analysis plan, contributed to the design of the study protocol and data analyses. PI contributed to the design of the study protocol, supervision, monitoring and data analyses. RG was responsible for management and coordination of the trial. RC, AP, HP (Hariharan Periasamy), HA, SP and PJ contributed in designing the study protocol, data analyses and writing—review and editing. BV participated in data analysis particularly related to microbiology. BV had full access to study data and verified the data. MP and SB were involved in conceptualization, funding acquisition, designing of the study protocol and writing—review and editing. LL, RG, and PI had full access to and verified all raw data pertaining to this manuscript/study. All authors had access to the underlying data, approved the final version of the manuscript and SB had the final responsibility to submit for publication.

## Data sharing statement

Data from this study would be made available on reasonable request from healthcare providers, clinical trial investigators, and researchers to address specific scientific or clinical queries. Wockhardt will review such request positively for access to de-identified patient-level clinical trial data via email communications.

## Declaration of interests

HP (Himanshu Pophale) and MG received the investigator grant from Wockhardt for the study conduct. PI is a consultant for Wockhardt, India. LL, RG, RC, AP, SP, PJ, HP (Hariharan Periasamy), MP and SB are employees of Wockhardt, India. RG, RC, AP, SP, MP and SB own stock or stock options in Wockhardt. HA was employee of Wockhardt, India during the study conduct. BV has none to declare.

## Appendix A Supplementary data

Protocol Phase 3 Nafithromycin

Statistical analysis plan
