# Supplementary material for: Efficacy and safety of a 3-day once-daily regimen of oral nafithromycin in comparison to oral moxifloxacin for the treatment of community-acquired bacterial pneumonia in adults: a phase III, randomized, double-blind controlled trial
Source: Lancet Reg Health Southeast Asia. 2025 Sep 23;41:100666. doi: 10.1016/j.lansea.2025.100666 (PMC12494923; doi:10.1016/j.lansea.2025.100666)
Supplement: Protocol Phase 3 Nafithromycin [file mmc1.pdf]

1 TITLE PAGE

Protocol Title	A Phase III, Randomised, Multicentre, Double-Blind, Comparative Study to Determine the Efficacy and Safety of Oral Nafithromycin Versus Oral Moxifloxacin in the Treatment of Community-Acquired Bacterial Pneumonia (CABP) in Adults
Short Title	Phase III study of oral nafithromycin in CABP
Protocol Number	W-4873-301
Study Drug	Nafithromycin
Original Protocol Date	26 June 2018
Amendment (if any)	Protocol Amendment 03
Protocol Amendment Date	5 February 2019
Sponsor	Wockhardt Bio AG<br>Grafenauweg 6<br>Zug-6300, Switzerland<br>Phone: +41-417275220<br>Fax: +41-417275221
Sponsor Medical Monitor	Dr Mushtaque Mastim
IND Number	123465

Confidentiality Statement

The information contained herein is the property of Wockhardt Bio AG, Grafenauweg 6, Zug-6300, Switzerland. Use, reproduction, issue, loan or disclosure of the contents of this document to third parties in any form whatsoever is not permitted without written authority from us except that this document may be disclosed to appropriate institutional review committees so long as they are requested to keep it confidential. The information given in this document may not be used or made public without our explicit consent, and is to be regarded as a trade secret in that it contains unpublished results of our research which are used in our business.

2 PROTOCOL APPROVAL PAGE

**Protocol Number**	W-4873-301
**Protocol Title**	A Phase III, Randomised, Multicentre, Double-Blind, Comparative Study to Determine the Efficacy and Safety of Oral Nafithromycin Versus Oral Moxifloxacin in the Treatment of Community-Acquired Bacterial Pneumonia (CABP) in Adults
**IND Number**	123465
**Version**	Protocol Amendment 03
**Date**	5 February 2019

Personnel Approving Protocol	Signature/Date
Name<br>Project Lead	Dr Manishkumar D Shah<br>07 Feb 2019
Name<br>Medical Monitor	Dr. MUSHTAQUE MASTIM<br>Mushtaque 07/Feb/2019.
Name<br>Head, Global Clinical Development	DR. ASHIMA BHATIA<br>Ashima Bhatia 07 Feb 2019

3 REVISION HISTORY

Version Number	Date	Summary of Changes
Final Protocol	26 June 2018	Not applicable
Protocol Amendment 01	13 August 2018	As Given below
Protocol Amendment 02	22 October 2018	As Given below
Protocol Amendment 03	5 February 2019	As Given below

Protocol Amendment 01, 13 August 2018: Summary of Changes:

Section No.(Protocol Amendment 01)	Section name	Original Text	Amended Text	Rationale
Section 6	Synopsis	PORT Risk Class II will be capped at 50%	PORT Risk Class II will be capped at 60%	To clarify a specific limit of cap rather than a range
Section 11.1 and Section 20.3.1	Description of the study design and Enrolment/Randomisation/Masking Procedures	PORT Risk Class II will be capped at 50 to 60%	PORT Risk Class II will be capped at 60%	To clarify a specific limit of cap rather than a range
Section 6, Section 11.2.1.2 and section 18.2.2	Synopsis, Secondary Efficacy Endpoints and Clinical Outcome at EOT and TOC.	Clinical Failure: The signs and symptoms of CABP (dyspnoea, production of purulent sputum or pleuritic chest pain) did not resolve and/or cough worsened, such that non-study antibacterial therapy must be initiated for the treatment of CABP or death occurred prior to assessment or subject received alternative anti microbial therapy for the treatment of CABP prior to this visit.	Clinical Failure: The signs and symptoms of CABP (dyspnoea, production of purulent sputum or pleuritic chest pain) did not resolve or return to premorbid and/or cough worsened, such that non-study antibacterial therapy must be initiated for the treatment of CABP or death occurred prior to assessment or subject received alternative anti microbial therapy for the treatment of CABP prior to this visit.	To further clarify the criteria for the clinical failure
Section 14.1.3	Product Storage and Stability	• Storage and dispensing of nafithromycin tablets can be undertaken at room temperature (below 25°C) in its packaging (while protected from moisture). Detailed instructions regarding storage are presented in the IB.<br>• Levofloxacin tablets should be stored at 15° to 30°C (59° to	• Storage and dispensing of nafithromycin tablets and corresponding clinical placebo tablets can be undertaken at room temperature (below 25°C) in its packaging (while protected from moisture). Detailed instructions regarding storage are presented in the IB.<br>• Levofloxacin tablets and corresponding	Inclusion of clinical placebo for the storage condition

Section No.(Protocol Amendment 01)	Section name	Original Text	Amended Text	Rationale
		86°F) in well-closed containers.	clinical placebo capsules should be stored at 15° to 30°C (59° to 86°F) in well-closed containers.	
Section 14.1.5.2 and Section 14.1.2	Levofloxacin and Formulation, Appearance, Packaging and Labelling	Removed the following text from Section 13.1.5.2, Levofloxacin: Levofloxacin or matching may be presented as over-encapsulated tablet.	Added the following text to Section 13.1.2, Formulation, Appearance, Packaging and Labelling	Administrative change
Section 19.3	Time Period and Frequency for Event Assessment and Follow up	Deaths occurring till 30 days after the last day of study participation should be reported, if Investigator becomes aware of the same.	Deaths occurring within 40 days of first dose should be reported, if Investigator becomes aware of the same.	This allows for reporting up to 30 days after an end of therapy visit
Section 20.1.8.2	Secondary Efficacy Analyses	For each clinical outcome, the number and percentage of subjects with each response category (e.g., for the TOC Visit: Cure, Failure or Indeterminate) will be tabulated by treatment group and site group and baseline pathogen (obtained from respiratory or blood cultures, antigen or serology tests).	For each clinical outcome, the number and percentage of subjects with each response category (e.g. for the TOC Visit: Cure, Failure or Indeterminate) will be tabulated by treatment group and baseline pathogen (obtained from respiratory or blood cultures, antigen or serology tests).	For each clinical outcome, the number and percentage of subjects with each response category (e.g., for the TOC Visit: Cure, Failure or Indeterminate) will not be tabulated by site group
Section 20.1.11	Interim Analysis	No formal interim analysis of efficacy is planned. Periodic reviews of unblinded accumulated safety data will be performed by the DMC.	No formal interim analysis of efficacy is planned.	To clarify that Internal Data Monitoring Committee review of the data is not linked to interim analysis
Section 20.3.1	Enrolment/Randomisation/Masking Procedures	Subjects will be randomly assigned to 1 of the 2 treatment arms using an IXRS in the ratio of 1:1. Enrolment of PORT Risk Class II will be capped at 50% to 60% and enrolment of subjects with allowed prior systemic antibiotic use will be capped initially at 25%	Subjects will be randomly assigned in blinded manner to 1 of the 2 treatment arms using an IXRS in the ratio of 1:1. Enrolment of PORT Risk Class II will be capped at 60% and enrolment of subjects with allowed prior systemic antibiotic use will be capped initially at	Clarification for the randomisation assignment

Section No.(Protocol Amendment 01)	Section name	Original Text	Amended Text	Rationale
		(subject to change during study conduct).	25% (subject to change during study conduct).	
Section 6 and Section 9.2.1	Synopsis and Rationale for Therapeutic Treatment With 800 mg of Nafithromycin Once Daily for 3 Days	Deletion of following statement: "No SAEs have been reported for any dose in clinical studies conducted to date."	Not applicable	Rectification of error

Protocol Amendment 02, 22 October 2018: Summary of Changes:

Section No.(Protocol Amendment 02)	Section name	Original Text	Amended Text	Rationale
Section 1, Section 6, Section 9.2.2, Section 13 and all applicable sections	Title, Synopsis, Rationale for Moxifloxacin as Comparator, Study Treatment and all applicable places	Levofloxacin 500mg PO q24h for 7 days	Moxifloxacin 400mg PO q24h for 7 days	Recommendation of Investigational New Drug Committee, Directorate General of Health Services, India
Section 6 and Section 12.2	Synopsis and Exclusion Criteria	• NA<br>• Known infection with human immunodeficiency virus (HIV) (before Screening) and a cluster of differentiation 4 (CD4) count that is unknown or documented to be < 200 cells/mm3 within the last year or an Acquired Immune Deficiency Syndrome (AIDS)-defining illness; note that neither HIV nor CD4 testing is required at Screening	• Active or suspected pulmonary tuberculosis<br>• History or diagnosis of human immunodeficiency virus (HIV)	Recommendation of Investigational New Drug Committee, Directorate General of Health Services, India
Section 6, Section 14.1, Section 14.1.3 and Section 23.4	Synopsis, Screening Visit, Local Laboratory Assessments and 23.4 Appendix IV: Safety Laboratory Tests Conducted by the Central Laboratory	NA	HIV by serology	Recommendation of Investigational New Drug Committee, Directorate General of Health Services, India
Section 6,	Synopsis, Study	NA	Best possible efforts	To provide operational

Section No.(Protocol Amendment 02)	Section name	Original Text	Amended Text	Rationale
Section 14.6 and Section 14.7	Day 5 Visit and Study Day 6 Visit		should be made to conduct an in-person visit. In the event that an in-person visit is not possible due to any reason, assessment may be conducted through a telephone contact.	flexibility
Section 14.1.2	Radiographic Evaluation	If a CXR is performed, anteroposterior and lateral views are preferred, and confirmation of new or progressive pulmonary infiltrates consistent with CABP may occur with either (or both) CXR view; however, posteroanterior (i.e., portable) views are also acceptable.	If a CXR is performed, posteroanterior and lateral views are required (unless it is medically not feasible) along with confirmation of new or progressive pulmonary infiltrates consistent with CABP. Anteroposterior (i.e., portable) views are also acceptable if performing posteroanterior and lateral views is medically not feasible.	To clarify required radiographic evaluations for the purpose of eligibility assessment
Section 6, Table 1. Schedule of Assessments	Synopsis, Table 1. Schedule of Assessments	Blood will be collected for central laboratory testing at the Screening, Day 5, EOT and TOC visits; blood will also be collected for central laboratory testing at Day 3 for serum transaminases (ALT and AST) and total bilirubin levels. Local laboratory evaluations will be conducted at FU in subjects with clinically significant laboratory abnormalities noted at or after the TOC Visit.	Blood will be collected for central laboratory testing at the Screening, Day 3, EOT and TOC visits. Local laboratory evaluations will be conducted at FU in subjects with clinically significant laboratory abnormalities noted at or after the TOC Visit.	Rectification of error
Section 6 and Section 11.1	Synopsis and Description of Study Design	Subjects will be assessed daily by the Investigator on Day 1 through Day 7, irrespective of the treatment setting (inpatient or outpatient).	Subjects will be assessed daily by the Investigator on Day 1 through Day 4, irrespective of the treatment setting (inpatient or outpatient).	Rectification of error

Protocol Amendment 03, 5 February 2019: Summary of Changes:

Section No.(Protocol Amendment 03)	Section name	Original Text	Amended Text	Rationale
Section 6 and Section 12.2	Synopsis and Exclusion Criteria	History or diagnosis of human immunodeficiency virus (HIV)	Known history or diagnosis of human immunodeficiency virus (HIV) infection by serology. In the event that the results of HIV serology are not available promptly, the total white blood cell count is ≥500 cells per cubic millimetre, and all the other eligibility criteria are met, the subject can be randomized and given the first dose of study treatment for management of CABP. If the HIV serology is positive, the subject can continue in the study if the Investigator deems it will not interfere with optimal study participation (e.g. evaluation of study drug efficacy, determination of safety, or completion of the expected course of treatment) and medical management of the underlying CABP. Investigators should discuss all positive cases with the Medical Monitor.	For confirmed exclusion of HIV cases
Section 6, Section 12.2 and Section 19.1.5	Synopsis, Exclusion Criteria and Clinically Evaluable Analysis Set	Active or suspected pulmonary tuberculosis	A: Active or suspected pulmonary tuberculosis (TB).<br><br>B: At Indian sites, the subject not agreeing to diagnostic evaluation of tuberculosis by Xpert TB test (using GeneXpert)#<br><br>#*In the event that the results of Xpert TB*	Recommendation of Investigational New Drug Committee, Directorate General of Health Services, India

Section No.(Protocol Amendment 03)	Section name	Original Text	Amended Text	Rationale
			test are not available promptly and all the other eligibility criteria are met, the subject can be randomized and given the first dose of study treatment for management of CABP. The subject will be discontinued from study therapy prior to the second dose if the results of Xpert TB test suggest "detection" of Mycobacterium tuberculosis complex (MTBC) and is indicative of active pulmonary tuberculosis.)<br><br>Subjects discontinued from study due to detection of MTBC indicative of active pulmonary tuberculosis will be excluded from Clinically Evaluable Analysis Set.	
Section 6, Section 10, Section 11.2, Section 17 and Section 19	Synopsis, Statistical Methods, Study Objectives, Study Endpoints, Efficacy Evaluation and Statistical Methods	ITT Analysis set	MITT Analysis Set	This modification allows the power of the study to be maintained in lieu of eligibility criteria amendment which excludes subjects with tuberculosis
Section 6 and Section 14.1.1	Table 1. Schedule of Assessments, footnote "g"	Assess CABP symptom severity (Appendix III, Section 23.3)	At screening visit, assessment of premorbid symptoms (approximately 7 days prior to onset of current CABP symptoms) should be conducted. Assess present as well as premorbid (approximately 7 days	To collect data relevant to premorbid symptoms for assessment of clinical outcome

Section No.(Protocol Amendment 03)	Section name	Original Text	Amended Text	Rationale
			prior to onset of current CABP symptoms) CABP symptom severity (Appendix III, Section 23.3)	
Section 6, Section 16.1 and Section 14.1.6	Synopsis, Table 1. Schedule of Assessments, Screening Respiratory Specimens, Microbiological Assessments	NA	An expectorated sputum sample will be collected for diagnostic evaluation of tuberculosis by Xpert TB test (using GeneXpert) in addition to collection of expectorated sputum or other deep respiratory sample for microbiological assessments as per protocol (section16.1). Collection of two sputum/respiratory samples, on the day of screening, could be scheduled at the discretion of investigator based on convenience of the subject.	Recommendation of Investigational New Drug Committee, Directorate General of Health Services, India
Section 18.4.2	Serious Adverse Event Reporting	Initial SAE Form must be duly completed, signed and dated by the Investigator/ designee and sent to PV team and Global Clinical Development Department, Wockhardt Ltd within 24 hours of occurrence of the event via email or fax to the attention of the following: Common email: drugsafety@wockhardt.com Fax Number for sites in India: +91-22-26523885	Initial SAE Form must be duly completed, signed and dated by the Investigator/ designee and sent to PV team and Global Clinical Development Department, Wockhardt Ltd or designated CRO within 24 hours of occurrence of the event via email or fax. Relevant contact information will be provided to the clinical study sites.	Administrative changes to allow potential participation of other countries in the study.
Section 6 and Section 12	Synopsis, Statistical Methods and Study Population	Sample size: Approximately 414 adult subjects diagnosed with CABP	Sample size: Approximately 488 adult subjects diagnosed with CABP	This modification allows the power of the study to be maintained with respect to study objectives in lieu of eligibility criteria

Section No.(Protocol Amendment 03)	Section name	Original Text	Amended Text	Rationale
				amendment which excludes subjects with tuberculosis
Section 6 and Section 11.1	Synopsis and Description of Study Design	NA, text added.	Hospitalisation for convenience or social purposes may be permitted at the discretion of investigator. Such convenience or social admissions should be documented in electronic Case Report Form (eCRF), however, will not qualify as admission due to Serious Adverse Event (SAE) will also not qualify for the relevant secondary efficacy assessment. The investigator shall seek necessary approval for or notify ethics committee about convenience /social admissions, as per local requirements.	Administrative reasons
Section 4 and Section 20	Statement of Compliance , Ethics/Protection of Human Subjects	International Conference for Harmonisation (ICH). This study will be conducted in compliance with the Code of Conduct for Research Involving Human Volunteers as issued by the ICH-GCP and the principles of the Declaration of Helsinki.	International Council for Harmonisation (ICH)<br>Declaration of Helsinki by World Medical Association.	Rectification of error
Section 5, Section 6, Section 9.1, Section 9.2.2 and Section 18	Signature Page, Synopsis: Study Centres (Planned), Background and Rationale, Rationale for Moxifloxacin, Background Information, Rationale for Moxifloxacin as Comparator, Safety	NA	NA	In the listed sections, administrative changes have been made to allow potential participation of other countries in the study.

Section No.(Protocol Amendment 03)	Section name	Original Text	Amended Text	Rationale
	Evaluation			
Section 19.1.12	Interim Analysis	NA	A blinded (aggregated across treatment groups) review of the percentage of subjects in the ITT population who tested positive for MTBC (as per the results of the Xpert TB test) will be conducted when approximately 50% of subjects have been enrolled. If the percentage subjects who tested positive for MTBC is higher than anticipated, the target number of enrolled subjects may be increased to ensure the study is sufficiently powered for the MITT analyses set.	This modification allows the power of the study to be maintained with respect to study objectives in lieu of eligibility criteria amendment which excludes subjects with tuberculosis
Section 23.1	Appendix I: Allowed and Disallowed Prior Antibiotics	NA	Added Omadacycline and Clarithromycin XL in disallowed Medicines	Rectification of error
Section 23.3	Appendix IV: Safety Laboratory Tests Conducted by Central lab	Deleted HIV by Serology, Coagulation: Prothrombin time/International normalized ratio, partial thromboplastin time	NA	Rectification of error, Listed tests will be done at local laboratory
Section 5, Section 6, Section 8, Section 10.2, Section 11.2.1, Section 12, Section 12.3, Section 12.5, Section 12.6.1, Section 13. 1, Section 13.2, Section 14, Section 15.1, Section 17.2, Section 18, Section 19 and Section 20	Signature page, synopsis, List of Abbreviations, Secondary objectives, Efficacy endpoints, Study Population, Exclusion criteria, Strategies for Recruitment and Retention, Premature discontinuation from study drug administration, Study Agents Control Description, Concomitant	NA	NA	In the listed sections relevant edits have been made for the purposes of correction of typographical errors, providing clarifications and incorporating administrative changes.

Section No.(Protocol Amendment 03)	Section name	Original Text	Amended Text	Rationale
	therapy, Study Procedures and conduct (microbiological assessment), Pharmacokinetic Blood Sample Collection, Clinical outcome assessment, Safety Evaluation, Statistical Methods, Ethics / Protection of Human Subjects.			
Section 6 and Section 12.3	Synopsis and Exclusion Criteria	• Compromised hepatic or renal function, including but not limited to: aspartate aminotransferase or alanine aminotransferase > 3 times ULN<br>• History of tendon disorders related to fluoroquinolone administration	• Compromised hepatic or renal function, including but not limited to: aspartate aminotransferase or alanine aminotransferase ≥ 3 times ULN<br>• History of tendon disorders	Rectification of error
Section 6 and Section 9.1	Synopsis and Background Information	• Approximately 30 to 45 study centres in India<br>• NA	• Approximately 30 to 45 study centres in India and Latin America<br>• *Streptococcus pneumoniae* was identified as the most common pathogen, accounting for up to 35% of CAP cases in Latin America. The second most commonly identified organism was *Staphylococcus aureus* in 17% of culture-positive cases. *Haemophilus influenzae* was also an important cause of CAP, identified as the causative agent in 23.2% of adult pneumonia cases in the Regional System	To allow potential participation of Latin America for accommodating enrolment of additional subjects for the power of the study to be maintained with respect to study objectives in lieu of eligibility criteria amendment which excludes subjects with tuberculosis

Section No.(Protocol Amendment 03)	Section name	Original Text	Amended Text	Rationale
			for Vaccines II (SIREVA II) study between 2000 and 2005. The most frequently occurring atypical pathogens were *Mycoplasma pneumoniae* (13%), *Chlamydia pneumoniae* (6%), and *Legionella pneumophila* (3%). The mean rate of CAP due to penicillin-resistant *S. pneumoniae* was 39%. The mortality in Latin America due to lower respiratory tract infections has been reported to be 6%, compared with 4% in developed regions, and CAP was the third most frequent cause of death in adults in 31 Latin American countries in 2001–2003.	

4 STATEMENT OF COMPLIANCE

The study will be carried out in accordance with Good Clinical Practice (GCP) as required by the following:

- Schedule Y guidelines (rules 122A, 122B, 122D, 122DA, 122DAA and 122E). Requirements And Guidelines For Permission To Import And / Or Manufacture Of New Drugs For Sale Or To Undertake Clinical Trials, Drugs and Cosmetics Rules, 1945, India
- United States (US) Code of Federal Regulations (CFR) applicable to clinical studies (45 CFR Part 46, 21 CFR Part 50, 21 CFR Part 56 and 21 CFR Part 312)
- International Council for Harmonisation (ICH) E6 (R2); INTEGRATED ADDENDUM TO ICH E6 (R1): GUIDELINE FOR GOOD CLINICAL PRACTICE. Step 4 version dated 09 November 2016
- The principles of Declaration of Helsinki by World Medical Association.

5 SIGNATURE PAGE

The signatures below provide the necessary assurances that this trial will be conducted according to all stipulations of the protocol, including all statements regarding confidentiality, in the Declaration of Helsinki, ICH-GCP guidelines and other applicable national and regional laws and regulations.

- The Investigators will comply with procedures for data recording and reporting
- The Investigators will permit monitoring, auditing and inspection by the Sponsor, its designated representatives and regulatory authorities

Site Investigator:

Signature: ______________________________ Date: ____________

Name
Title

6 SYNOPSIS

**Study Number**	W-4873-301
**Title of the Study**	A Phase III, Randomised, Multicentre, Double-Blind, Comparative Study to Determine the Efficacy and Safety of Oral Nafithromycin Versus Oral Moxifloxacin in the Treatment of Community-Acquired Bacterial Pneumonia (CABP) in Adults
**Study Centres (Planned)**	Approximately 30 to 45 study centres in India and Latin America
**Development Phase**	III
**Background and Rationale**	Lower respiratory tract infections (LRTIs), including community-acquired bacterial pneumonia (CABP), are among the five leading causes of death and years of life lost worldwide; the associated age-standardised death rate for LRTIs was 36.8 per 100,000 population in 2016 worldwide. The global incidence of pneumonia is estimated to be between 1.5 and 14.0 cases per 1000 person-years, varying by region, season and patient characteristics. Short-term mortality (in-hospital and 30-day mortality) for hospitalised patients with CABP ranges from 4.0% to 18.0%. Costs related to CABP are high, and few approaches (such as reducing the length of hospital stay, adequate use of antibiotics and the introduction of vaccines) have reduced these costs to date. *Streptococcus pneumoniae* remains the predominant pathogen of CABP worldwide, independent of age. Other common pathogens include *Haemophilus influenzae*, *Moraxella catarrhalis*, *Staphylococcus aureus* and atypical bacteria (including *Mycoplasma pneumoniae*, *Chlamydophila pneumoniae* and *Legionella pneumophila*).<br><br>A major therapeutic challenge affecting the treatment of CABP is the widespread resistance of *S. pneumoniae* to ß-lactams (especially penicillins and cephalosporins) and macrolides. The prevalence of multi-drug-resistant (MDR) *S. pneumoniae* is also worrisome. Community-acquired MDR *S. pneumoniae* poses treatment challenges for outpatient CABP—most notably the lack of effective oral antibacterial options—because of resistance to the most commonly used oral penicillins, cephalosporins and/or macrolides. Failure of therapy due to resistance continues to contribute to the morbidity and mortality of CABP, and treatment failures of even mild disease result in increased hospitalisation and contribute to increased healthcare costs.<br><br>Nafithromycin (also known as WCK 4873) is an oral antibacterial agent of the ketolide class, which is structurally related to the macrolide class. Diverse in vitro, in vivo, preclinical pharmacokinetic (PK) and safety studies have provided strong scientific evidence of the therapeutic potential of nafithromycin for difficult-to-treat respiratory tract infections such as CABP caused by MDR pathogens. Preclinical studies have demonstrated that nafithromycin has potent activity against macrolide- and ketolide-resistant strains of *S. pneumoniae* and Group A streptococci. Additionally, nafithromycin has in vitro activity against other important CABP pathogens such as methicillin-susceptible *S. aureus* (MSSA), *H. influenzae*, *M. catarrhalis* and atypical pathogens (*M. pneumoniae*, *C. pneumoniae* and *L. pneumophila*).<br><br>The ketolide class of antibiotics provides a convenient orally administered and effective therapeutic option for pneumococci, streptococci and Gram-negative respiratory pathogens. Innovative structural modifications among newer ketolides provide them with several distinguishing features such as high-affinity binding to domain V and domain II of the 23S ribosomal ribonucleic acid (rRNA) target, in contrast to macrolides that bind only to domain V. Such dual-target binding by ketolides overcomes multiple macrolide resistance mechanisms such as (1) *erm* gene-encoded methylases (macrolide, lincosamide and streptogramin B-type resistance, involving the methylation of the 23 rRNA target), (2) point mutations within rRNA domain V and (3) diverse mutations in ribosomal proteins L-4 and L-22. Additionally, newer ketolides such as nafithromycin are not susceptible to *mef* efflux pump-mediated resistance that affects the activity of macrolides against *S. pneumoniae*

	and *Streptococcus pyogenes*. Thus, these newer ketolides possess several features that not only confer activity against various resistotypes, irrespective of ß-lactam and macrolide susceptibility, but also minimise the risk of resistance emergence or induction of cross-resistance to other agents. A new ketolide antibiotic with improved coverage of MDR respiratory pathogens, in particular those resistant to the older macrolides, would help address this unmet need.<br>Nafithromycin is expected to have the following advantages over older, available macrolides based on data from in vivo, in vitro and preclinical studies conducted to date:<br>1. Comprehensive coverage of key CABP pathogens, including atypical pathogens<br>2. Mechanism-based activity potential against macrolide- and ketolide-resistant pneumococci and Group A streptococci<br>3. Excellent target-organ-tissue concentration leading to:<br>a. Enabling potent activity versus MDR pathogens in vivo<br>b. Once-a-day dosing convenience<br>c. Potential for shorter duration of therapy<br>4. Minimal cytochrome P450 (CYP) inhibition leading to ease of co-administration of other drugs<br>5. Favourable hepatic safety potential owing to favourable drug disposition resulting from lower accumulation in the liver
**Objectives**	**Primary Objectives**<br>• To demonstrate that oral nafithromycin is non-inferior to oral moxifloxacin in the clinical response at Day 4 in the Modified Intent-to-Treat (MITT) analysis set<br>• To assess overall safety of oral nafithromycin in the safety analysis set<br>**Secondary Objectives**<br>• To assess the clinical response at Day 4 in the Microbiological Modified Intent-to-Treat (mMITT) and Clinically Evaluable (CE) analyses sets<br>• To assess the clinical outcome at End of Treatment (EOT) in the MITT, mMITT and CE analyses sets<br>• To assess the clinical outcome at Test of Cure (TOC) in the MITT, mMITT and CE analyses sets<br>• To assess re-admission to the hospital (or admission to the hospital if not previously hospitalised) for any reason before Follow-Up (FU) (Day 31 ± 4 days) in the MITT analysis set<br>• To determine the PK of oral nafithromycin (in PK analysis set)<br>• To assess by-pathogen clinical response at Day 4 and by-pathogen clinical outcome at TOC in the mMITT analysis set
**Study Endpoints**	**Primary Efficacy Endpoints**<br>*Clinical Response at Day 4 (MITT Analysis Set)*<br>• Favourable Clinical Response: Alive and improvement of at least 1 level (e.g., severe to moderate, moderate to mild, mild to absent) in at least 2 CABP symptoms (dyspnoea, cough, production of purulent sputum or pleuritic chest pain) compared with that at the Baseline Visit, without worsening in any other of the 4 CABP symptoms. Severity of symptoms is based on a 4-point scale (absent, mild, moderate or severe).<br>• Unfavourable Clinical Response: No improvement of at least 1 level in at

	least 2 CABP symptoms compared with that at baseline; or worsening in any of the 4 CABP symptoms compared with that at baseline; or death from any cause at or before Day 4.<br>• Indeterminate: Study data are missing for evaluation of efficacy at Day 4 for any reason, including loss to FU.<br>**Secondary Efficacy Endpoints**<br>*Clinical Response at Day 4 (mMITT and CE Analyses Sets)*<br>• Favourable Clinical Response, Unfavourable Clinical Response and Indeterminate: Definitions are as mentioned in the Primary Efficacy Endpoints section<br>*Clinical Outcome at EOT (MITT, mMITT and CE Analyses Sets)*<br>• Clinical Cure: Alive and signs and symptoms of CABP (dyspnoea, production of purulent sputum or pleuritic chest pain) are resolved or return to premorbid conditions and cough is improved such that further antibacterial therapy is not needed and the subject otherwise cannot be declared a clinical failure.<br>• Clinical Failure: The signs and symptoms of CABP (dyspnoea, production of purulent sputum or pleuritic chest pain) did not resolve or return to premorbid and/or cough worsened, such that non-study antibacterial therapy must be initiated for the treatment of CABP or death occurred prior to assessment or subject received alternative anti-microbial therapy for the treatment of CABP prior to this visit.<br>• Indeterminate: Study data are missing for the evaluation of efficacy at the assessment visit for any reason, including loss to FU.<br>*Clinical Outcome at TOC (MITT, mMITT and CE Analyses Sets)*<br>• Clinical Cure, Clinical Failure or Indeterminate: Definitions are as above. Failures at EOT will be carried forward to TOC.<br>*Hospitalisation Prior to FU (MITT Analysis Set)*<br>• Hospital re-admission for any reason between the Day 1 and the FU visits, if previously hospitalised and discharged or initial hospital admission for any reason between the Day 2 and the FU visits (Day 31 ± 4 days), if not previously hospitalised on Day 1.<br>*By-Pathogen Clinical Response at Day 4 and By-Pathogen Clinical Outcome at TOC in the mMITT Analysis Set*<br>• The by-subject clinical response at Day 4 and clinical outcome at TOC defined above will be applied to each pathogen to obtain the:<br>▪ By-pathogen Clinical Response at Day 4: Favourable, Unfavourable or Indeterminate (definitions as mentioned above)<br>▪ By-pathogen Clinical Outcome at TOC: Clinical Cure, Clinical Failure or Indeterminate (definitions as mentioned above)<br>**Safety Endpoints**<br>• Incidence of subjects with treatment-emergent adverse events (TEAEs), serious adverse events (SAEs) and discontinuations due to TEAEs<br>• Incidence of subjects with potentially clinically significant (PCS) changes in safety laboratory parameters, vital signs and electrocardiograms (ECGs)
**Study Design**	This is a Phase III, prospective, multicentre, randomised, double-blind, comparative efficacy and safety study of oral nafithromycin versus oral moxifloxacin for the treatment of male and female adults with CABP. Subjects providing informed consent and meeting all study eligibility criteria will be enrolled in the study and randomised in a 1:1 ratio to either of the following 2 treatment arms:

	• **Nafithromycin** 800 mg (two 400-mg tablets) orally (PO) every 24 hours (q24h) for 3 days; subjects will receive matching placebo PO q24h on Day 4 through EOT (2 tablets) and matching moxifloxacin placebo PO q24h, on Day 1 through EOT (1 capsule), to maintain the blind (2 tablets and 1 capsule in total).<br>• **Moxifloxacin** 400 mg (1 capsule: Over-encapsulated tablet) PO q24h for 7 days; subjects will receive matching nafithromycin placebo PO q24h on Day 1 through EOT (2 tablets), to maintain the blind (2 tablets and 1 capsule in total).<br>Study drugs should be administered approximately at the same time every day through the treatment period.<br>Baseline assessments for study eligibility will occur during the Screening visit, within 24 h before the administration of the first dose of the study drug. Block randomisation using an interactive voice/web response system (IXRS), stratified by Pneumonia Outcomes Research Team (PORT) Risk Class (II vs. III/IV) will be used to assign subjects (1:1) to 1 of the 2 study treatment arms.<br>The enrolment of subjects with PORT Risk Class II will be capped at 60% and the enrolment of subjects with allowed prior systemic antibiotic use will be capped initially at 25% (subject to change during the study conduct). Study Day 1 will be defined as the day when the study drug is first administered, and subsequent study days will be defined by the number of consecutive calendar days thereafter.<br>Subjects may be treated in the study as inpatients or outpatients based on their clinical condition, at the discretion of the Investigator; however, only the oral study drug will be administered. Hospitalisation for convenience or social purposes may be permitted at the discretion of investigator. Such convenience or social admissions should be documented in electronic Case Report Form (eCRF), however, will not qualify as admission due to Serious Adverse Event (SAE) and will also not qualify for applicable secondary efficacy assessment. The investigator shall seek necessary approval for or notify ethics committee about convenience /social admissions, as per local requirements. Subjects will be assessed daily by the Investigator on Day 1 through Day 4, irrespective of the treatment setting (inpatient or outpatient). Investigators will assess for clinical outcome at EOT (+ 2 days) and TOC (Day 15 ± 4 days). A FU Visit will be conducted on Day 31 ± 4 days from the start of treatment. Best possible efforts should be made to conduct an in-person FU Visit on Day 31 ± 4 days. In the event that an in-person visit is not possible due to any reason, assessment may be conducted through a telephone contact for subjects who were considered to be Clinical Cures and had no adverse events (AEs) or clinically significant laboratory or ECG abnormalities noted at or after the TOC Visit. Such logistical reasons should be documented. For the subjects who had AEs or clinically significant laboratory or ECG abnormalities noted at or after the TOC Visit, an in-person FU Visit must be conducted.<br>**Rationale for moxifloxacin as comparator:**<br>Moxifloxacin is a member of the fluoroquinolone class of antibiotics. It is active against most CABP pathogens including macrolide- and penicillin-resistant *S. pneumoniae*, Gram-negative bacteria, and atypical pathogens. Empiric treatment of CABP with moxifloxacin, especially in subjects admitted to the hospital, is consistent with current US (Infectious Diseases Society of America [IDSA]/American Thoracic Society [ATS]) and European (The Task Force of the European Respiratory Society in collaboration with the European Society for Clinical Microbiology and Infectious Diseases [ESCMID]) therapeutic guidelines. The recommended dose (IDSA/ATS) is 400 mg once daily for 7-14 days, and in this study, a 7-day regimen will be used.<br>**Rationale for therapeutic treatment with 800 mg of nafithromycin once daily for 3 days:**<br>Preclinical PK conducted in rodent and non-rodent species and Phase I clinical studies have shown optimal PK profile commensurate to the once-a-day dosing potential of

	nafithromycin. Administration of multiple ascending oral doses (600 mg, 800 mg and 1000 mg) of nafithromycin to humans was well tolerated. Clinical doses of a novel respiratory antibiotic are justified based on the probability of attaining pharmacodynamic targets (PDTs) that have been identified in a murine neutropenic lung infection model. PDTs refer to the ratio of area under the concentration-time curve (AUC) to the MIC required for causing 1 log_{10} kill of the infecting baseline pathogen isolated from the lung. Nafithromycin clinical doses have been justified taking into account both serum and epithelial lining fluid (ELF) exposures. The magnitude of AUC/MIC was associated with 1 log_{10} kill employing mouse serum and ELF PK against all *S. pneumoniae* (n=6, including penicillin- and macrolide-resistant) strains for nafithromycin. Using Phase I data, a population PK model was built and a 2000 subject Monte Carlo Simulation was undertaken for the probability of target attainment (PTA) analysis. PTA analyses showed that a nafithromycin regimen of 800 mg once daily for 3 days would both provide > 90% PTA for contemporary *S. pneumoniae* strains taking into account both plasma- and ELF-based PDTs. Attainment of high PTA suggests that a nafithromycin dose of 800 mg once daily administered over 3 days would be clinically effective against key contemporary respiratory tract infection pathogens. Interestingly, the PTA was not adversely affected even in fed subjects, suggesting that nafithromycin efficacy would remain consistent for both fed and fasted subjects. Moreover, a nafithromycin dose of 800 mg once daily for 3 days would also provide coverage of penicillin-resistant, quinolone-resistant, azithromycin-resistant and telithromycin-non-susceptible *S. pneumoniae* isolates expressing a high level of MDR.<br><br>Nafithromycin attained a geometric mean half-life of approximately 11 h at steady state at 800 mg/day in healthy human subjects (steady state was achieved on average within 3-4 days over the 600 to 1000 mg/day dose range). In addition, significant accumulation of nafithromycin in polymorphonuclear cells was observed.<br><br>Furthermore, high tissue penetration observed in rodents, particularly in the target organ lung, is expected to drive favourable pharmacodynamic activity, enabling a shorter duration of therapy of 3 to 5 days.<br><br>A multiple dose study was conducted to compare plasma, ELF and alveolar macrophage (AM) concentrations of nafithromycin (800 mg administered once daily for 3 days) in healthy adult subjects. Each subject underwent 1 standardised bronchoscopy with bronchoalveolar lavage at 3, 6, 9, 12, 24 or 48 h after the third oral dose of nafithromycin. Concentrations of nafithromycin were significantly higher in ELF and AM than in simultaneous sampled plasma concentrations throughout the 48-h period after 3 days of once-daily dosing. The ratios of ELF to plasma concentrations and of AM to plasma concentrations based on the mean plasma concentration versus time curve over 24 h (AUC_{0-24}) values were 13.8 and 527, respectively.<br><br>In the intrapulmonary study, nafithromycin demonstrated therapeutically significant ELF concentrations even at 12, 24 and 48 h after the third (last) dose (9.7, 4.1 and 1.6 µg/mL, respectively). These concentrations were above the MICs of nafithromycin against global collection of *S. pneumoniae* strains (MIC_{90} of 0.06 µg/mL for all *S. pneumoniae* tested and 0.12 µg/mL for macrolide-and telithromycin-resistant *S. pneumoniae*). These sustained high concentrations observed in ELF even 48 h after the last dose provide a potential basis of shorter duration (3 days) of nafithromycin therapy in subjects with CABP.<br><br>Compared with oral moxifloxacin 400 mg once a day for 7 days, oral nafithromycin 800 mg once a day for 3 days was found to be safe, effective and well tolerated in a Phase II, randomised, double-blind, multinational study.<br><br>**Pharmacokinetic sampling**:<br><br>Blood samples for PK analysis will be collected from subjects on Day 1, Day 3 and Day 4 at sites where PK sampling is possible. Time points for PK sample collection will be as follows:

	• A pre-dose PK sample will be collected within 10 min before dosing on Day 3.<br>• Post-dose PK samples will be collected at 2-4 h (Day 1 and Day 3) and 24-28 h (On Day 4, i.e. 24-28 h after Day 3 dose). Subjects who have been hospitalised are also required to have a post-dose PK sample at 6-10 h on Day 1 and Day 3.
**Inclusion Criteria**	Subjects are required to meet all of the following inclusion criteria:<br>1. Male or female ≥ 18 years of age<br>2. Willing to participate in the study and provide written informed consent before any protocol-specific assessment is performed; consent from a legal guardian is not acceptable<br>3. Meet the following clinical criteria for CABP:<br>a. Have at least TWO of the following symptoms (new or worsening):<br>• Dyspnoea (shortness of breath)<br>• Cough<br>• Production of purulent sputum<br>• Pleuritic chest pain<br>b. Have at least TWO of the following vital sign abnormalities:<br>• Fever or hypothermia documented by the Investigator (oral, rectal or tympanic temperature > 38.0°C [100.4°F] or < 36.0°C [95.5°F])<br>• Hypotension, defined as systolic blood pressure < 90 mmHg<br>• Tachycardia, defined as heart rate > 90 beats per minute<br>• Tachypnoea, defined as respiratory rate > 20 breaths per minute<br>c. Have at least ONE of the following clinical signs or laboratory abnormalities:<br>• Hypoxaemia defined as arterial oxygen saturation < 90% by pulse oximetry or partial pressure of arterial oxygen < 60 mmHg by arterial blood gas<br>• Auscultatory findings on pulmonary examination consistent with bacterial pneumonia or pulmonary consolidation (e.g. rales, dullness on percussion, bronchial breath sounds or egophony)<br>• Elevated total white blood cell (WBC) count (> 10,000 cells/mm^3) or leucopenia (WBC < 4000 cells/mm^3)<br>• Elevated immature neutrophils (> 15% band forms), regardless of total peripheral WBC count<br>d. Radiographic evidence of CABP:<br>• Radiographically confirmed pneumonia, i.e., new or progressive pulmonary infiltrate(s) on chest X-ray or chest computed tomography scan consistent with acute bacterial pneumonia within 48 h before randomisation<br>e. PORT score of 51 to 105 (PORT Risk Class of II, III or IV)<br>4. All females must have a negative urine or serum pregnancy test (beta-human chorionic gonadotropin) at Screening AND agree to the use of 1 of the following acceptable methods of contraception from Screening through TOC: surgical sterilisation (defined as bilateral oophorectomy or bilateral salpingectomy, but excluding bilateral tubal occlusion), post-menopausal women(defined by amenorrhea for at least 12 months following cessation of all exogenous hormonal treatments), barrier contraception (e.g. condom, intrauterine device), levonorgestrel intrauterine system (e.g. Mirena®), regular medroxyprogesterone injections (e.g. Depo-Provera®), sexual intercourse with only vasectomised partners or abstinence. (Note: Sexual abstinence is considered a highly effective method only if defined as refraining from heterosexual intercourse during the entire period of risk associated with the study treatments. The reliability of sexual abstinence needs to be evaluated in

	relation to the duration of the clinical trial and the preferred and usual lifestyle of the subject.) Note that oral contraceptives should not be used as the sole method of birth control because the effect of nafithromycin on the efficacy of oral contraceptives has not yet been established; subjects who take oral contraceptives must also use 1 of the acceptable forms of birth control (listed above) from Screening through TOC.<br>5. All males must agree to use an acceptable barrier method of birth control (i.e., condom) with female partner(s) and must not donate sperm from Screening through TOC.<br>6. Ability to ingest the intact oral study drug (e.g. able to swallow large capsules intact and no significant nausea, vomiting, diarrhoea or any other condition that might impair ingestion or absorption of the oral study drug)
**Exclusion Criteria**	Subjects should meet none of the following exclusion criteria:<br>1. Subjects with any of the following confirmed or suspected types of pneumonia:<br>• Aspiration pneumonia<br>• Hospital-acquired bacterial pneumonia, defined as pneumonia with onset of clinical signs and symptoms after at least 48 h hospitalisation in an acute inpatient healthcare facility<br>• Healthcare-associated bacterial pneumonia, defined as pneumonia acquired in a long-term care or subacute healthcare facility (e.g. nursing home) or pneumonia with onset after recent hospital discharge (within 90 days of current admission and previously hospitalised for ≥ 48 h)<br>• Ventilator-associated bacterial pneumonia, defined as pneumonia with onset of clinical signs and symptoms after at least 48 h of endotracheal intubation<br>• Pneumonia that may be caused by pathogen(s) resistant to any study drug (nafithromycin, moxifloxacin), including viral, mycobacterial or fungal pneumonia (e.g. *Pneumocystis jiroveci* pneumonia, active pulmonary tuberculosis)<br>• Post-obstructive pneumonia<br>• Pneumonia associated with cystic fibrosis, bronchiectasis or any other chronic pulmonary disease<br>2. Suspected or confirmed pleural empyema (a parapneumonic pleural effusion is not an exclusion criterion) or lung abscess<br>3. Suspected or confirmed non-infectious causes of pulmonary infiltrates (e.g. pulmonary embolism, hypersensitivity pneumonia, congestive heart failure)<br>4. Receipt of 1 or more dose(s) of a potentially effective systemic antibacterial treatment for treatment of the current CABP within 72 h before randomisation except if the prior therapy is a single dose of a short-acting antibacterial agent (Appendix I in Section 23.1 for allowable prior antibiotics); subjects requiring concomitant adjunctive or additional potentially effective systemic antibacterial treatment for management of CABP<br>5. Evidence of significant immunologic disease determined by any of the following:<br>• Current or anticipated neutropenia defined as < 500 neutrophils/mm^3<br>• Known history or diagnosis of human immunodeficiency virus (HIV) infection by serology. In the event that the results of HIV serology are not available promptly, the total white blood cell count is ≥ 500 cells per cubic millimetre, and all the other eligibility criteria are met, the subject can be randomised and given the first dose of study treatment for management of CABP. If the HIV serology is positive, the subject can continue in the study if the Investigator deems it will not interfere with optimal study participation (e.g. evaluation of study drug efficacy, determination of safety, or completion of the expected course of

	treatment) and medical management of the underlying CABP. Investigators should discuss all positive cases with the Medical Monitor.<br>• History of heart, lung or kidney transplant<br>• The receipt of cancer chemotherapy, radiotherapy or potent, non-corticosteroid immunosuppressant drugs (e.g. cyclosporine, azathioprine, tacrolimus, immune-modulating monoclonal antibody therapy) within the past 3 months or the receipt of corticosteroids equivalent to or greater than 40 mg of prednisone per day for more than 14 days in the 30 days before randomisation<br>6. Known or suspected primary or metastatic neoplastic lung disease, bronchiectasis, cystic fibrosis, bronchial obstruction, chronic neurological disorder preventing clearance of pulmonary secretions or severe chronic obstructive pulmonary disease (severe chronic obstructive pulmonary disease is defined as known [before Screening] ratio of forced expiratory volume in 1 second (FEV_1) to forced vital capacity [FVC] < 0.70 and FEV_1 < 50% normal); note that pulmonary function tests are not required at Screening<br>7. Compromised hepatic or renal function, including but not limited to: clinical evidence of end-stage liver disease (e.g. ascites, hepatic encephalopathy), screening serum total bilirubin > 2 times the upper limit of normal (ULN) (unless associated with an elevated indirect bilirubin typical of Gilbert syndrome), aspartate aminotransferase or alanine aminotransferase ≥ 3 times ULN, serum creatinine > 2.0 mg/dL, creatinine clearance < 50 mL/min, or blood urea nitrogen > 30 mg/dL. Other clinically significant abnormal laboratory findings should be discussed with the Medical Monitor before the subject's entry<br>8. History of *Clostridium difficile*-associated disease within 6 months before enrolment<br>9. History of hypersensitivity, known contraindication (e.g. lactose intolerance, lactase deficiency or glucose-galactose malabsorption etc.) or allergic reaction (e.g. anaphylaxis, urticaria, other significant reaction) to any ketolide or fluoroquinolone antibiotic<br>10. Current second- or third-degree atrioventricular block or sick sinus syndrome, uncontrolled atrial fibrillation, severe or unstable angina, congestive heart failure, myocardial infarction within 3 months before the Screening visit, clinically significant ECG abnormalities including QT interval corrected for heart rate using Fridericia's formula (QTcF) > 450 ms (males) or > 470 ms (females) or requirement for medications known to cause QT prolongation<br>11. Prior (within 14 days before randomisation) or concomitant use of CYP liver enzyme inducers (e.g. phenobarbital, carbamazepine, griseofulvin, sulfonylureas, phenytoin or rifampin)<br>12. Current peripheral neuropathy or myasthenia gravis<br>13. Known or suspected seizure disorder or other central nervous system disorders that may predispose the subject to seizures or lower the seizure threshold<br>14. Nursing mother or pregnant female<br>15. Subjects who received any experimental drug within 30 days before enrolment<br>16. Require admission to an intensive care unit for any reason, life expectancy of less than 2 months or any concomitant condition that, in the opinion of the Investigator, is likely to interfere with evaluation of the response of the infection under study, determination of AEs or completion of the expected course of treatment<br>17. History of tendon disorders<br>18. A: Active or suspected pulmonary tuberculosis (TB).<br>B: At Indian sites, the subject not agreeing to diagnostic evaluation of

	tuberculosis by Xpert TB test (using GeneXpert).[#]<br><br>*[#] In the event that the results of Xpert TB test are not available promptly and all the other eligibility criteria are met, the subject can be randomized and given the first dose of study treatment for management of CABP. The subject will be discontinued from study therapy prior to the second dose if the results of Xpert TB test suggest "detection" of Mycobacterium tuberculosis complex (MTBC) and is indicative of active pulmonary tuberculosis.*
**Study Visits**	Screening Visit (within 24 h before randomisation)<br>Day 1: Randomisation and first dose of the study drug, includes PK sampling<br>Day 2<br>Day 3: Includes PK sampling<br>Day 4: Includes PK sampling<br>Day 5: Best possible efforts should be made to conduct an in-person visit. In the event that an in-person visit is not possible due to any reason, assessment may be conducted through a telephone contact.<br>Day 6: Best possible efforts should be made to conduct an in-person visit. In the event that an in-person visit is not possible due to any reason, assessment may be conducted through a telephone contact.<br>EOT: Clinical outcome assessment (on Day 7 or within 2 days after Day 7)<br>TOC: Day 15 ± 4 days (inclusive)<br>FU: Day 31 ± 4 days (Best possible efforts should be made to conduct an in-person FU Visit on Day 31 ± 4 days. In the event that an in-person visit is not possible due to any reason, assessment may be conducted through a telephone contact for subjects who were considered to be Clinical Cures and had no AEs or clinically significant laboratory or ECG abnormalities noted at or after the TOC Visit. Such logistical reasons should be documented. For the subjects who had AEs or clinically significant laboratory or ECG abnormalities noted at or after the TOC Visit, an in-person FU Visit must be conducted.)
**Investigational Medicinal Product: Test Product and Matching Placebo**	**Nafithromycin:**<br>Strength and pharmaceutical dosage form: 400 mg tablet<br>Dose: 800 mg once daily (2 tablets of 400 mg each) on Day 1 through Day 3<br>Route of Administration: Oral<br>**Placebo to match nafithromycin:**<br>Dosage form: tablet<br>Dose: 2 placebo tablets on Day 4 through EOT (for subjects on nafithromycin arm), 2 placebo tablets on Day 1 through EOT (for subjects on moxifloxacin arm)<br>Route of administration: Oral
**Reference Therapy and Matching Placebo**	**Moxifloxacin:**<br>Strength and pharmaceutical dosage form: 400 mg capsule (over-encapsulated tablet)<br>Dose: 400 mg once daily for 7 days<br>Route of Administration: Oral<br>**Placebo to match moxifloxacin:**<br>Dosage form: capsule<br>Dose: 1 capsule on Day 1 through EOT (for subjects on nafithromycin arm)<br>Route of administration: Oral
**Study Duration**	Each subject will remain in the study for approximately one month. This will include a Screening/Baseline visit (within 24 h of randomisation), a 7-day oral treatment period and post-treatment assessments at TOC (Day 15 ± 4 days) and FU (Day 31 ± 4 days).
**Sample Size**	Approximately 488 adult subjects diagnosed with CABP, will be enrolled in the study Using a 12.5% non-inferiority margin, 1-sided alpha of 0.025 and the Farrington-Manning approach for sample size determination, a total of 414 (207/arm) subjects per treatment group are required in the MITT analysis set so that the Miettinen and

	Nurminen test will have 90% power to compare oral nafithromycin to oral moxifloxacin, when the expected proportion of subjects with a favourable clinical response at Day 4 is 0.810. For the determination of this endpoint, subjects with missing data or who are lost to FU, will have an Indeterminate outcome at Day 4. These subjects will be included in the denominator for the calculation of proportion of subjects with favourable clinical response at Day 4. Given the number of subjects needed in the MITT analyses set (N=414) and assuming a TB rate of 15%, 488 subjects will need to be enrolled into the study.
**Statistical Methods**	**Efficacy:**<br>Efficacy will be analysed in the following analyses sets according to the randomised treatment assignment: nafithromycin or moxifloxacin<br>• ITT analysis set: All subjects who were randomised<br>• MITT analysis set: All subjects who are randomized and received at least one dose of study drug. Subjects with detection of MTBC (as per results of Xpert TB test) indicative of active pulmonary tuberculosis will be excluded from this analysis set.<br>• mMITT analysis set: All MITT subjects who have at least one baseline pathogen known to cause CABP against which the investigational drug has antibacterial activity, including bacterial pathogens identified by respiratory specimen culture, blood culture, and /or urinary antigen test (*S. pneumoniae*, *S. aureus*, *Haemophilus influenzae*, *Haemophilus parainfluenzae*, *Moraxella catarrhalis* and *L. pneumophila*) or atypical bacterial serologic response (*M. pneumoniae*, *C. pneumoniae*, *L. pneumophila*). Subjects with sole baseline Gram-negative pathogens from the *Enterobacteriaceae, Pseudomonadaceae or Yersiniaceae* families will be excluded from this analysis set<br>• CE analysis set: subjects in the MITT analysis set that followed important components of the trial as defined in section 19.1.5<br>The primary efficacy endpoint will be the clinical response at Day 4 in the MITT analysis set. For this endpoint, the number and percentage of subjects in each response category will be summarised by treatment group. For the primary efficacy analysis, the 2-sided 95% confidence intervals (CIs) for the observed difference between treatment groups (nafithromycin minus moxifloxacin) in the proportion of subjects with favourable clinical response at Day 4 in the MITT analysis set will be calculated using the Miettinen-Nurminen score test. If the lower bound of the 2-sided 95% CI is greater than -0.125, the non-inferiority of nafithromycin treatment as compared to moxifloxacin treatment will be concluded. In addition, if the lower limit of the 2-sided 95% CI is greater than zero, superiority of nafithromycin as compared to moxifloxacin will be concluded.<br>In addition, a sensitivity analysis will be conducted using the Miettinen-Nurminen statistic stratified by the randomisation factor: PORT (II vs III/IV) score.<br>The clinical response at Day 4 will also be compared between treatment groups in the mMITT and CE analyses sets.<br>For the by-subject secondary efficacy endpoints (e.g. clinical outcome at EOT and TOC), the number and percentage of subjects in each response category will be provided by treatment group in the MITT, mMITT and CE analyses sets (see Table 6). The 2-sided 95% CIs for the difference between treatment groups in the proportion of subjects with favourable response (e.g. proportion of subjects with clinical cure at EOT) will be provided in each analysis set.<br>Summaries of the proportions of subject with a favourable clinical response at Day 4 and the proportions of subjects with clinical cure at TOC in the subgroup of subjects with bacteraemia at baseline and the subgroup of subjects with at least one MDR pathogen will be provided using the mMITT analysis set.<br>The by-pathogen clinical response at Day 4 and by-pathogen clinical outcome at TOC will be summarised in the mMITT analysis set. Full details of all analyses will be provided in the Statistical Analysis Plan.

	**Safety:** Safety data will be summarised for the safety analysis set which includes all treated subjects according to the treatment actually received. The number and percentage of subjects in each treatment group reporting at least 1 occurrence of a TEAE for each unique Medical Dictionary for Regulatory Activities (MedDRA) System Organ Class (SOC) and Preferred Term (PT) will be tabulated. A TEAE is defined as an AE occurring or worsening on or after the administration of the first dose of the study drug (see Section 18.1.1). The incidence of subjects with TEAEs will also be tabulated by, severity and by relationship to the study drug as assessed by the Investigator for each treatment group and PT within each SOC. The number and percentage of subjects in each treatment group reporting at least 1 occurrence of a treatment-emergent SAE will be tabulated. The number and percentage of subjects (in each treatment group) prematurely discontinuing study drug treatment due to a TEAE will be tabulated by SOC and PT.<br>Safety laboratory data will be summarised using descriptive statistics of the baseline and post-baseline values and the changes from baseline, as well as the number and percentage of subjects with PCS changes in laboratory values. Descriptive statistics of vital signs and ECG parameters and the change from baseline will also be presented. Vital sign changes will be classified as PCS, and the number and percentage of subjects with a post-baseline PCS vital sign will be provided. PCS changes in ECG parameters will be tabulated.<br>**Pharmacokinetics:** PK parameters will be summarised in the PK analysis set. This includes subjects in the safety analysis set (from eligible sites where PK sampling is possible) who received at least 1 dose of oral nafithromycin and had at least 1 analysable plasma PK sample. Descriptive statistics for the blood concentrations at each timepoint and PK parameters (C_{max} and AUC) will be summarised in the PK analysis set. Plasma concentrations of nafithromycin will be listed for each subject in the PK analysis set.<br><br>The PK data acquisition and analysis strategy entails the use of a sparse PK sampling schedule. Efforts will be made to obtain PK samples from all subjects at sites where PK sampling is possible.

Table 1. Schedule of Assessments

Visits	Screening Visit (Day -1)[a]	Day 1[b]	Day 2	Day 3	Day 4	Day 5[w]	Day 6[w]	EOT Day 7 (+ 2 days)[c]	TOC Day 15 (± 4 days)[d]	FU Day 31 (± 4 days)[e]
Informed Consent[f]	√									
Medical history and demography	√									
Physical exam, including CABP symptom severity[g]	√	√	√	√	√			√	√	
Vital signs, including oximetry[h]	√	√	√	√	√			√	√	
12-Lead ECG[i]	√			√				√	√[i]	√[i]
CXR or chest CT scan[j]	√									
PORT score calculation[k]	√									
Inclusion/exclusion criteria	√	√								
Randomisation		√								
Laboratory assessments										
Haematology, coagulation, serum chemistry[l]	√			√				√	√	√
Serology for HIV[l]	√									
Atypical pathogen serology[m]	√								√	√
Urine tests[n]	√									
Urinary antigen testing[o]	√									
Pregnancy test[p]	√								√	
Respiratory specimen Gram stain/culture[q]	√	As clinically indicated								
Blood culture[r]	√	Repeat the blood cultures as necessary until negative blood cultures are obtained								
Blood for PK sampling[s]		√		√	√					
Study drug administration and accountability[t]		√	√	√	√	√	√	√		
Adverse Events[u]	√	√	√	√	√	√	√	√	√	√
Prior and concomitant medications[v]	√	√	√	√	√	√	√	√	√	√

**Clinical outcome assessment (by the investigator)**								√	√	

Abbreviations: AE = adverse event; ALT = alanine aminotransferase; AST = aspartate aminotransferase; CABP = community-acquired bacterial pneumonia; CT = computed tomography; CXR = chest X-ray; ECG = electrocardiogram; EOT = end-of-treatment; FU = follow-up; PK = pharmacokinetic; PORT = Pneumonia Outcomes Research Team; TOC = Test of Cure

[a]Following signing of the informed consent form, all Screening evaluations should be completed within 24 h before randomisation.
[b]Day 1 is the first day of study drug administration. Subsequent study days are consecutive calendar days. If feasible, Screening and randomisation procedures (Screening visit and Day 1) can be performed on the same day. Standard of care data from within 24 h (laboratory) and 48 h (radiology) before randomisation can be used as Screening visit procedures.
[c]EOT is to be conducted on or within 2 days after Day 7. EOT Visit procedures should also be conducted for any premature withdrawal from the study or the study drug.
[d]TOC is to be conducted on Day 15 ± 4 days.
[e]Best possible efforts should be made to conduct an in-person FU Visit on Day 31 ± 4 days. In the event that an in-person visit is not possible due to any reason, assessment may be conducted through a telephone contact for subjects who were considered to be Clinical Cures and had no AEs or clinically significant laboratory or ECG abnormalities noted at or after the TOC Visit. Such logistical reasons should be documented. For the subjects who had AEs or clinically significant laboratory or ECG abnormalities noted at or after the TOC Visit, an in-person follow up visit must be conducted.
[f]Written and signed informed consent must be obtained before any protocol assessment is performed.
[g]Complete physical exam—consisting of general appearance, the skin, eyes, ears, nose, throat, lungs, heart, abdomen, back, extremities, lymph nodes, vascular and neurological exams—will be conducted at Screening and daily between Day 1 and Day 4 (whether inpatient or outpatient), EOT and TOC. As part of the complete physical exam, the Investigator should assess the severity of the subject's CABP symptoms of dyspnoea, cough, production of purulent sputum and pleuritic chest pain, based on the CABP Symptom Severity Guidance for Investigator Assessment (Appendix III). At screening visit, assessment of premorbid symptoms (approximately 7 days prior to onset of current CABP symptoms) should be conducted.
[h]Vital signs—including body temperature (oral, rectal or tympanic), blood pressure, pulse rate, respiratory rate and pulse oximetry—will be collected at Screening and daily between Day 1 and Day 4 (whether inpatient or outpatient), EOT and TOC. Height, weight and creatinine clearance (CrCl) will also be collected at the Screening visit.
[i]A 12-lead ECG will be performed at Screening, Day 3 and EOT. A 12-lead ECG will be performed at TOC or FU if prior ECG(s) showed any clinically significant abnormality.
[j]Subjects must have a confirmatory CXR or chest CT scan consistent with acute bacterial pneumonia within 48 h before randomisation.
[k]Subjects with a PORT score of 51 to 105 (PORT Risk Class of II, III or IV) are eligible for enrolment.
[l]At Screening, local laboratory evaluations required for assessing subject eligibility include serum aminotransferase (ALT and AST) and total bilirubin levels, serum creatinine and blood urea nitrogen (or urea), peripheral white blood cell (WBC) count, absolute neutrophil count, coagulation and immature neutrophil percentage and serology for HIV. Blood will be collected for central laboratory testing at the Screening, Day 3, EOT and TOC visits. Local laboratory evaluations will be conducted at FU in subjects with clinically significant laboratory abnormalities noted at or after the TOC Visit.
[m]Collect blood for acute (Screening) and convalescent (TOC) atypical pathogen serology, including *M. pneumoniae*, *C. pneumoniae* and *L. pneumophila*, for central laboratory testing.
[n]At Screening, a urine dipstick will be performed locally; if results are abnormal and deemed clinically significant by the Investigator, a urinalysis will be sent to the central laboratory.
[o]At Screening, urine will be collected for central laboratory testing of *S. pneumoniae* and *L. pneumophila*. These tests will be done by the central laboratory using rapid antigen test kits.

[p]At Screening, a local laboratory urine or serum pregnancy test (females only) is required to confirm study eligibility. In addition, blood will be collected from all female subjects for serum β-human chorionic gonadotropin pregnancy test by the central laboratory at the Screening and TOC visits.

[q]At Screening, the collection of expectorated sputum or other deep respiratory sample should be attempted in all subjects. Gram staining will be performed on all sputum specimens and quality will be assessed. Culture will be performed on all sputum samples of adequate quality or deep respiratory specimens. For subjects being enrolled at Indian sites, an expectorated sputum sample will be collected for diagnostic evaluation of tuberculosis by Xpert TB test (using GeneXpert) in addition to collection of expectorated sputum or other deep respiratory sample for microbiological assessments as per protocol (section16.1). Collection of two sputum/respiratory samples, on the day of screening, could be scheduled at the discretion of investigator based on convenience of the subject. Post-baseline respiratory specimens should be collected as clinically indicated and from subjects who are clinical failures and require alternative antibacterial treatment for CABP.

[r]Two sets of blood cultures (each set consists of 1 aerobic and 1 anaerobic blood culture bottle) will also be collected at Screening. If baseline blood cultures are positive, repeated post-baseline blood cultures should be collected until a negative result is obtained. Sites may wait until confirmation of results of previous cultures to collect further samples.

[s]Blood samples for PK analysis will be collected from all subjects at sites where PK sampling is possible on Day 1, Day 3 and Day 4, including within 10 min before study drug administration on Day 3 and after dosing at 2-4 h (Day 1 and Day 3) and 24-28 h (On Day 4, i.e. 24-28 h after Day 3 dose). Subjects who have been hospitalised are also required to have a post-dose PK sample at 6-10 h (Day 1 and Day 3).

[t]Study drug should be administered q24h (± 4 h) between Day 1 and EOT, with the exception of Day 2 in which an additional window of 4 h may be utilised, depending on the randomisation time on Day 1.

[u]AEs and SAEs will be recorded and reported from signing of the informed consent to the FU Visit.

[v]Prior medications that have been administered within 14 days before the date of signing the informed consent or during the Screening Phase will be recorded in the electronic Case Report Form (eCRF). All medications administered after the first dose of the study drug must be recorded in the eCRF.

[w]Best possible efforts should be made to conduct an in-person visit. In the event that an in-person visit is not possible due to any reason, assessment may be conducted through a telephone contact.

7 OVERALL TABLE OF CONTENTS

1 TITLE PAGE....1
2 PROTOCOL APPROVAL PAGE....2
3 REVISION HISTORY....3
4 STATEMENT OF COMPLIANCE....14
5 SIGNATURE PAGE....15
6 SYNOPSIS....16
7 OVERALL TABLE OF CONTENTS....30
7.1 List of Tables....35
8 LIST OF ABBREVIATIONS....36
9 INTRODUCTION: BACKGROUND INFORMATION AND SCIENTIFIC RATIONALE....39
9.1 BACKGROUND INFORMATION....39
9.1.1 Nafithromycin....41
9.1.2 Phase I Studies of Nafithromycin....41
9.1.3 Phase II Studies of Nafithromycin....42
9.2 RATIONALE....43
9.2.1 Rationale for Therapeutic Treatment With 800 mg of Nafithromycin Once Daily for 3 Days....44
9.2.2 Rationale for Moxifloxacin as Comparator....45
9.3 POTENTIAL RISKS AND BENEFITS....46
9.3.1 Known Potential Risks....46
9.3.2 Known Potential Benefits....46
10 STUDY OBJECTIVES....48
10.1 PRIMARY OBJECTIVES....48
10.2 SECONDARY OBJECTIVES....48
11 STUDY DESIGN AND ENDPOINTS....49
11.1 DESCRIPTION OF THE STUDY DESIGN....49
11.1.1 Pharmacokinetic Sampling....50
11.2 STUDY ENDPOINTS....50
11.2.1 Efficacy Endpoints....50
11.2.2 Safety Endpoints....51
12 STUDY POPULATION....52
12.1 SUBJECT ELIGIBILITY, ENROLMENT, AND WITHDRAWAL....52
12.2 INCLUSION CRITERIA....52
12.3 EXCLUSION CRITERIA....53
12.4 SCREEN FAILURES....55
12.5 STRATEGIES FOR RECRUITMENT AND RETENTION....56
12.6 CRITERIA FOR PREMATURE DISCONTINUATION OF STUDY DRUG....56
12.6.1 Premature Discontinuation from Study Drug Administration....56

12.6.2 Guidance to Investigators on When to End Study Drug Therapy 57

13 STUDY TREATMENT 59
13.1 STUDY AGENTS AND CONTROL DESCRIPTION 59
13.1.1 Acquisition 59
13.1.2 Formulation, Appearance, Packaging and Labelling 59
13.1.3 Product Storage and Stability 59
13.1.4 Preparation 59
13.1.5 Dosing and Administration 59
13.1.6 Route of Administration 60
13.1.7 Starting Dose and Dose Escalation Schedule 60
13.1.8 Dose Adjustments/Modifications/Delays 60
13.1.9 Duration of Therapy 60
13.2 CONCOMITANT THERAPY 60
13.3 PROHIBITED MEDICATIONS 60
13.4 BLINDING AND UNBLINDING PROCEDURES 60

14 STUDY PROCEDURES AND CONDUCT 62
14.1 SCREENING VISIT 62
14.1.1 Clinical Assessments 62
14.1.2 Radiographic Evaluation 62
14.1.3 Local Laboratory Assessments 63
14.1.4 PORT Score 63
14.1.5 Central Laboratory Assessments 63
14.1.6 Microbiological Assessments 64
14.2 STUDY DAY 1 64
14.2.1 Clinical Assessments 64
14.2.2 Pharmacokinetic Assessments 65
14.3 STUDY DAY 2 65
14.3.1 Clinical Assessments 65
14.3.2 Microbiological Assessments 65
14.4 STUDY DAY 3 65
14.4.1 Clinical Assessments 65
14.4.2 Central/Local Laboratory Assessments 65
14.4.3 Pharmacokinetic Assessments 65
14.4.4 Microbiological Assessments 66
14.5 STUDY DAY 4 66
14.5.1 Clinical Assessments 66
14.5.2 Pharmacokinetic Assessments 66
14.5.3 Microbiological Assessments 66
14.6 STUDY DAY 5 66
14.6.1 Clinical Assessments 66
14.6.2 Microbiological Assessments 66
14.7 STUDY DAY 6 67
14.7.1 Clinical Assessments 67

14.7.2 Microbiological Assessments.....67
14.8 STUDY DAY 7 (+ 2 DAYS) (END OF TREATMENT).....67
14.8.1 Clinical Assessments.....67
14.8.2 Central/Local Laboratory Assessments.....67
14.8.3 Microbiological Assessments.....67
14.9 STUDY DAY 15 (± 4 DAYS) (TEST-OF-CURE).....68
14.9.1 Clinical Assessments.....68
14.9.2 Central Laboratory Assessments.....68
14.9.3 Microbiological Assessments.....68
14.10 STUDY DAY 31 (± 4 DAYS) (FOLLOW-UP VISIT).....68
14.10.1 Clinical Assessments.....68
14.10.2 Central/Local Laboratory Assessments.....69
14.10.3 Microbiological Assessments.....69
14.11 UNSCHEDULED VISIT.....69

15 PHARMACOKINETIC ASSESSMENTS.....70
15.1 PHARMACOKINETIC BLOOD SAMPLE COLLECTION.....70
15.2 PHARMACOKINETIC ANALYSES.....70

16 MICROBIOLOGICAL ASSESSMENTS.....71
16.1 SCREENING RESPIRATORY SPECIMENS.....71
16.2 SCREENING BLOOD CULTURES.....72
16.3 SCREENING URINARY ANTIGEN TESTS.....72
16.4 SEROLOGY FOR ATYPICAL BACTERIAL TITERS.....72
16.5 POST-BASELINE MICROBIOLOGICAL ASSESSMENTS.....72
16.6 CENTRAL MICROBIOLOGY LABORATORY PROCEDURES.....73

17 EFFICACY EVALUATION.....74
17.1 PRIMARY AND SECONDARY EFFICACY VARIABLES.....74
17.1.1 Primary Efficacy Variable.....74
17.1.2 Secondary Efficacy Variables.....74
17.2 CLINICAL OUTCOME ASSESSMENTS.....74
17.2.1 Clinical Response on Day 4.....74
17.2.2 Clinical Outcome at EOT and TOC.....74
17.2.3 Hospitalisation Before the Follow-Up Visit.....75
17.3 BY-PATHOGEN CLINICAL RESPONSE AT DAY 4.....76
17.4 BY-PATHOGEN CLINICAL OUTCOME AT TOC.....76
17.5 EMERGENT INFECTIONS.....76

18 SAFETY EVALUATION.....77
18.1 SPECIFICATION OF SAFETY PARAMETERS.....77
18.1.1 Definition of Adverse Event.....77
18.1.2 Definition of Serious Adverse Event.....78
18.1.3 Definition of Unexpected Adverse Event.....78
18.2 CLASSIFICATION OF AN ADVERSE EVENT.....79
18.2.1 Severity of Event.....79

18.2.2 Relationship to Study Agent 79
18.3 TIME PERIOD AND FREQUENCY FOR EVENT ASSESSMENT AND FOLLOW-UP 79
18.4 REPORTING PROCEDURES 80
18.4.1 Adverse Event Reporting 80
18.4.2 Serious Adverse Event Reporting 80
18.4.3 Reporting of Pregnancies Occurring During the Study 81
18.5 SAFETY OVERSIGHT 82

19 STATISTICAL METHODS 83
19.1 ANALYSES SETS 83
19.1.1 Intent-to-Treat Analysis Set 83
19.1.2 Modified Intent to Treat (MITT) 83
19.1.3 Safety Analysis Set 83
19.1.4 Microbiological Modified Intent-to-Treat Analysis Set 83
19.1.5 Clinically Evaluable Analysis Set 83
19.1.6 Pharmacokinetic Analysis Set 84
19.1.7 Determination of Sample Size 84
19.1.8 Analysis of Disposition and Subject Characteristics 84
19.1.9 Efficacy Analyses 85
19.1.10 Safety Analyses 86
19.1.11 Pharmacokinetic Analyses 87
19.1.12 Interim Analysis 87
19.2 HANDLING OF DROPOUTS AND MISSING DATA 87
19.3 MEASURES TO MINIMISE BIAS 88
19.3.1 Enrolment/Randomisation/Masking Procedures 88

20 ETHICS/PROTECTION OF HUMAN SUBJECTS 89
20.1 ETHICAL STANDARD 89
20.2 INSTITUTIONAL REVIEW BOARD 89
20.3 INFORMED CONSENT PROCESS 89
20.3.1 Consent and Other Informational Documents Provided to Participants 89
20.3.2 Consent Procedures and Documentation 89
20.4 PARTICIPANT AND DATA CONFIDENTIALITY 90
20.4.1 Research Use of Stored Human Samples, Specimens or Data 90
20.5 FUTURE USE OF STORED SPECIMENS 90

21 STUDY MANAGEMENT 91
21.1 STUDY MONITORING 91
21.2 SOURCE DOCUMENTS AND ACCESS TO SOURCE DATA/DOCUMENTS 91
21.3 QUALITY ASSURANCE AND QUALITY CONTROL 91
21.4 DATA HANDLING AND RECORD KEEPING 91
21.4.1 Data Collection and Management Responsibilities 91
21.4.2 Study Records Retention 92
21.4.3 Protocol Deviations 92
21.4.4 Publication and Data Sharing Policy 92
21.5 FINANCING AND INSURANCE 92

22 LITERATURE REFERENCES 93

23 APPENDICES 97

23.1 Appendix I: Allowed and Disallowed Prior Antibiotics 97

23.2 Appendix II: PORT Score Calculation 98

23.3 Appendix III: CABP Symptom Severity Guidance for Investigator Assessment 100

23.4 Appendix IV: Safety Laboratory Tests Conducted by the Central Laboratory 101

7.1 List of Tables

Table 1. Schedule of Assessments 27

Table 2. Clinical Response Assessment on Day 4. 74

Table 3. Clinical Outcome Assessments at EOT and TOC. 75

Table 4. Hospitalisation Before the Follow-Up Visit. 76

Table 5. Emergent Infections. 76

Table 6. Secondary By-Subject Analysis Variables by Visit and Analyses Set. 86

8 LIST OF ABBREVIATIONS

Abbreviation	Definition
ABG	arterial blood gas
AE	adverse event
AIDS	Acquired Immune Deficiency Syndrome
ALT	alanine aminotransferase
AM	alveolar macrophage
ANSORP	Asian Network for Surveillance of Resistant Pathogens
ATS	American Thoracic Society
AST	aspartate aminotransferase
$AUC_{0\text{-}24}$	plasma concentration versus time curve over 24 h
ß-HCG	ß-human chorionic gonadotropin
BAL	bronchoalveolar lavage
CABP	community-acquired bacterial pneumonia
CBC	complete blood count
CDC	Centers for Disease Control and Prevention (US)
CD4	cluster of differentiation
CE	clinically evaluable
CFR	Code of Federal Regulations (US)
CI	confidence interval
CLSI	Clinical Laboratory Standards Institute (US)
CXR	chest X-ray
CYP	cytochrome P450
DMC	Data Monitoring Committee
ECG	electrocardiogram
eCRF	electronic case report form
EOT	End-of-Treatment
ELF	epithelial lining fluid
ESCMID	European Society for Clinical Microbiology and Infectious Diseases
EUCAST	European Committee on Antimicrobial Susceptibility Testing
FDA	Food and Drug Administration (US)
FDAAA	Food and Drug Administration Amendments Act of 2007 (US)
FU	Follow-Up

Abbreviation	Definition
GCP	Good Clinical Practice
HIV	human immunodeficiency virus
IB	Investigator's Brochure
ICF	informed consent form
ICH	International Council for Harmonisation
ICH E6	International Council for Harmonisation Guidance for Industry, Good Clinical Practice: Consolidated Guidance
IEC	Independent Ethics Committee
IDSA	Infectious Diseases Society of America
IRB	Institutional Review Board
ITT	Intent-to-Treat
IV	Intravenous
IXRS	interactive voice/web response system
LPF	low-power field
LRTI	lower respiratory tract infection
MDR	multi-drug resistant
MDRSP	multi-drug resistant S. pneumoniae
MIC	minimum inhibitory concentration
MITT	Modified Intent-to-Treat
mMITT	Microbiological Modified Intent-to-Treat
MSSA	methicillin-susceptible S. aureus
MTBC	Mycobacterium tuberculosis complex
NIH	National Institutes of Health
PaO2	partial pressure of arterial oxygen
PCS	potentially clinically significant
PDT(s)	Pharmacodynamic Target(s)
PK	pharmacokinetic(s)
PI	Principal Investigator
PISP	penicillin-intermediate S. pneumoniae
PMN	polymorphonuclear cell
PO	orally, per os or by mouth
PORT	Pneumonia Outcomes Research Team
PRSP	penicillin-resistant S. pneumoniae
PSSP	penicillin-sensitive S. pneumoniae
PT	preferred term

Abbreviation	Definition
PTA	probability of target attainment
PV	Pharmacovigilance
q24h	every 24 h
RTI	respiratory tract infection
rRNA	ribosomal ribonucleic acid
SAE	serious adverse event
SEC	squamous epithelial cell
SmPC	Summary of medicinal Product Characteristics
SOAR	Survey of Antibiotic Resistance
SOC	Standard of Care
TB	Tuberculosis
TEAE	treatment-emergent adverse event
TOC	Test of Cure
ULN	upper limit of normal
US	United States
WBC	white blood cell

9 INTRODUCTION: BACKGROUND INFORMATION AND SCIENTIFIC RATIONALE

9.1 BACKGROUND INFORMATION

Community-acquired bacterial pneumonia (CABP) is associated with considerable morbidity and mortality in adult patients and those with significant comorbidities (Global Burden of Disease, 2017). The lower respiratory tract infections (LRTIs) including CABP is among the five leading causes of death and years of life lost worldwide; the age-standardised death rate associated with LRTIs was 36.8 per 100,000 population in 2016 worldwide (Global Burden of Disease, 2017). According to the Centers for Disease Control and Prevention (CDC) estimates, about 1 million patients in the United States (US) are hospitalised for pneumonia and about 50,000 of them die from the disease each year (CDC, 2018). In the Asian region, pneumonia was diagnosed in a large proportion of hospitalised patients in the Philippines (19.9%), in a moderate proportion in Malaysia (6.4%) and in a relatively low proportion in Indonesia (1.5%) (Azmi et al, 2016). The global incidence of pneumonia in the geriatric population is estimated to be between 1.5 and 14.0 cases per 1000 person-years, varying by region, season and patient characteristics (File and Marrie, 2010; Millett et al, 2013). Short-term mortality (in-hospital and 30-day mortality) for hospitalised patients with CABP ranges from 4.0% to 18.0%. Costs related to CABP are high, and few approaches (such as reducing the length of stay, adequate use of antibiotics and the introduction of vaccines) have reduced these costs to date. *Streptococcus pneumoniae* remains the predominant pathogen of CABP worldwide, independent of age. The overall estimate of the annual incidence of CABP in India varies between 2 and 12 cases per 1000 population, with the highest rates reported in infants and the elderly (Ghoshal, 2016). Studies in Asia reported the 30-day mortality rate to be 7.3–8.6% (Song et al, 2008). Worldwide mortality of CABP in hospitalised patients was 14%, but increased to 20-50% in patients admitted to the intensive care unit (Vigg, 2016). The reported mortality rate in India is comparable to that reported elsewhere, with higher rates in the elderly patients and in those with coexisting comorbidities: 14% reported by Shah et al (2010a), 11% reported by Bansal et al (2004), 10.7% reported by Ravindranath and Raju (2016), 8% reported by Para et al (2018), 7.3% overall and 45.3% in the intensive care unit reported by Khadanga et al (2014) and 12.5%, 68.7% and 18.8% per the CURB-65 III, CURB-65 IV and CURB-65 criteria, respectively, reported by Shah et al (2010b). CABP is associated with both clinical and economic burden, based on its high incidence, admission rate, increased treatment costs and mortality rate (File and Marrie, 2010; Rosenbaum et al, 2015; Welte et al, 2012). Various approaches are being evaluated to reduce this burden by decreasing duration of therapy and dosage of antibiotics, treatment setting (home rather than hospital), switching to oral treatment from intravenous treatment in a short period of time and decreasing the duration of hospitalisation (Mertz and Johnstone, 2011).

While the aetiology of CABP may vary by geographic region and seasonality, *Streptococcus pneumoniae* remains the most common bacterial pathogen responsible for CABP worldwide; other pathogens are *Haemophilus influenzae*, *Moraxella catarrhalis*, *Staphylococcus aureus*, Gram-negative bacilli and atypical bacterial pathogens such as *Mycoplasma pneumoniae*, *Chlamydophila pneumoniae* and *Legionella pneumophilia* (Cilloniz et al, 2016). Limited data are available from India and most of the studies are geographically restricted. Capoor et al (2006) cited that *S. pneumoniae* (35.3%) was the most common isolate, followed by *S. aureus* (23.5%), *Klebsiella pneumoniae* (20.5%) and *H. influenzae* (8.8%) (Capoor et al, 2006). In another study in 225 patients admitted with CABP in a

hospital in North India, *S. pneumoniae*, *L. pneumophila* and influenza viruses were reported in > 10% of patients (Para et al, 2018). Also, variation was noted regarding the most common causative organism isolated in Indian studies. *S. pneumoniae* was found to be the most common aetiological agent of CABP in Shimla and Delhi whereas, in Ludhiana and Kashmir, it was *Pseudomonas aeruginosa* (Bansal et al, 2004; Oberoi et al, 2006; Shah et al, 2010). The organisms isolated from sputum and blood culture and detected by urine antigen testing in a prospective study from Mumbai included *S. pneumoniae, C. pneumoniae, H. influenzae, M. catarrhalis, M. pneumoniae, L. pneumophila, P. aeruginosa, Staphylococcus* spp. *and Salmonella typhi* (Udwadia et al, 2003).

Streptococcus pneumoniae was identified as the most common pathogen, accounting for up to 35% of Community Acquired Pneumonia (CAP) cases in Latin America. The second most commonly identified organism was *Staphylococcus aureus* in 17% of culture-positive cases. *Haemophilus influenzae* was also an important cause of CAP, identified as the causative agent in 23.2% of adult pneumonia cases in the Regional System for Vaccines II (SIREVA II) study between 2000 and 2005. The most frequently occurring atypical pathogens were *Mycoplasma pneumoniae* (13%), *Chlamydia pneumoniae* (6%), and *Legionella pneumophila* (3%). The mean rate of CAP due to penicillin-resistant *S. pneumoniae* was 39%. The mortality in Latin America due to lower respiratory tract infections has been reported to be 6%, compared with 4% in developed regions, and CAP was the third most frequent cause of death in adults in 31 Latin American countries in 2001–2003 (Isturiz et al, 2010).

CABP remains a common and serious illness despite the availability of new anti-microbial agents and vaccines. A major therapeutic challenge affecting the treatment of CABP is the widespread resistance of *S. pneumoniae* to ß-lactams (especially penicillin and first- and second-generation cephalosporins) and macrolide antibiotics. For example, the prevalence of *S. pneumoniae* macrolide resistance on a global scale has been estimated to be 25% to 40%, varying by region (Farrell et al, 2008; Reinert, 2009). The prevalence of multi-drug-resistant (MDR) *S. pneumoniae,* defined by penicillin nonsusceptibility plus resistance to two additional non-ß-lactam antibiotic classes, is also worrisome. Available data from the United States show that pneumococci are resistant to one or more antibiotics in about 30% of cases of invasive pneumococcal disease (CDC, 2018). The Asian Network for Surveillance of Resistant Pathogens (ANSORP) Study Group performed a prospective, multinational, hospital-based surveillance study in patients with pneumococcal infections in 11 Asian countries including India during 2008-2009 to investigate the current status of anti-microbial resistance and serotype distribution of *S. pneumoniae*. Current data show a persistently high prevalence of penicillin nonsusceptibility in Asian countries if the previous penicillin susceptibility Clinical Laboratory Standards Institute (CLSI) breakpoints of intermediate (0.12 to 1 µg/mL) and resistant (≥ 2 µg/mL) are applied. However, when the revised CLSI breakpoints are applied, the prevalence rate of penicillin-nonsusceptible pneumococci in nonmeningeal isolates was 4.6% and fully resistant isolates were found only in China (2.2%) and South Korea (0.3%). The overall rate of MDR in pneumococcal isolates was 59.3% (59.4% and 57.5% in nonmeningeal and meningeal isolates, respectively), with the highest MDR rate being 83.3% in China, followed by 75.5% in Vietnam, 63.9% in South Korea, 62.2% in Hong Kong and 59.7% in Taiwan. The most common pattern of MDR was resistance to cefuroxime, erythromycin, clindamycin and co-trimoxazole (20.2%), followed by resistance to erythromycin, clindamycin and co-trimoxazole (7.1%). All strains with MDR were resistant to at least 1 of the macrolides tested (Kim et al, 2012). Growing resistance of *S. pneumoniae* to the

most commonly used oral penicillins, cephalosporins and/or macrolides poses a major challenge to the therapeutic management of CABP. Another study, the Survey of Antibiotic Resistance (SOAR), was conducted during 2012–14 in Thailand, India, South Korea and Singapore. Using the CLSI iv breakpoint, 95.4% of the isolates from India were susceptible to penicillin (penicillin-sensitive *S. pneumoniae*, PSSP), whereas only 49.3% were PSSP according to the CLSI oral and the European Committee on Antimicrobial Susceptibility Testing (EUCAST) criteria. With these two breakpoints, 33.8% and 46.1% were scored as penicillin-intermediate *S. pneumoniae* (PISP), respectively, and 16.9% and 4.6% of isolates were penicillin-resistant *S. pneumoniae* (PRSP), respectively (Torumkuney et al, 2016). Failure of therapy due to MDR pathogens may lead to increased inpatient burden and healthcare costs, which could be substantial, because of morbidity and mortality attributed to CABP. Thus, a clinically effective and safe therapeutic alternative is clearly needed to treat MDR pathogens to alleviate the effect of this serious clinical and public health problem.

9.1.1 Nafithromycin

Nafithromycin, a novel synthetic ketolide, which is being investigated in clinical trials, is expected to provide clinicians with a new treatment option for CABP caused by the most common aetiologies of CABP, including the MDR pathogen *S. pneumoniae*, due to the ability of nafithromycin to overcome the dual resistance mechanism such as efflux and target mutation (Farrell et al, 2005; Sutcliffe et al, 1996).

Nafithromycin (also known as WCK 4873) is an oral antibacterial agent of the ketolide class that is structurally related to the macrolide class. Diverse in vitro, in vivo, preclinical, clinical pharmacokinetic (PK) and safety studies have provided strong scientific evidence towards therapeutic potential of nafithromycin for difficult-to-treat respiratory tract infections (RTIs) caused by MDR pathogens. Preclinical studies have shown that nafithromycin has potent activity against macrolide- and ketolide-resistant strains of *S. pneumoniae*, in addition to other important CABP pathogens such as *S. aureus*, Group A streptococci, *H. influenzae* and *M. catarrhalis*. A surveillance study involving 4,739 clinical isolates collected worldwide during 2013-2014 established the nafithromycin minimum inhibitory concentrations (MICs) for 50% and 90% of isolates (MIC_{50} and MIC_{90}) of 0.015 µg/mL and 0.06 µg/mL, respectively, against *S. pneumoniae* (Flamm et al, 2017). Against erythromycin- and telithromycin non-susceptible pneumococci, the nafithromycin MIC_{90} was 0.12 µg/mL. Similarly, for methicillin-susceptible *S. aureus* (MSSA), *H. influenzae, M. catarrhalis* and *S. pyogenes*, nafithromycin MIC_{90} values were 0.06, 4, 0.25 and 0.015 µg/mL, respectively (Flamm et al, 2017).

9.1.2 Phase I Studies of Nafithromycin

Clinical PK and safety of nafithromycin was studied in 117 healthy volunteers in 3 Phase I studies. All doses of nafithromycin administered in the single ascending dose, food effect, multiple ascending dose and bronchoalveolar lavage (BAL) trials were well tolerated in all subjects. The most commonly observed treatment-emergent adverse events (TEAEs) were dysgeusia, dizziness, headache, diarrhoea, nausea and abdominal discomfort. The maximum (or peak) serum concentration levels at the end of 7 days ranged from 1340 to 2987 ng/mL for the doses tested (7 days of 600-, 800- or 1000-mg doses). The corresponding area under the plasma concentration versus time curve over 24 h (AUC_{0-24}) on Day 7 ranged from 13,478 to 43,464 h•ng/mL. At Day 7, the half-life ($T_{½}$) ranged from 9.16

to 14.4 h. On average, steady-state values were observed starting from Day 3 for the 600-mg and 800-mg doses and Day 4 for the 1000-mg dose. The median time at which drug is present at maximum concentration in serum of nafithromycin occurred between 2 and 3.50 h after dosing. High intracellular accumulation was observed in polymorphonuclear cells (PMNs), with a maximum accumulation ratio of 138.6 for single-dose and 366.6 for multiple-dose (7-day dosing) nafithromycin, providing maximal average at the target of action.

In the BAL study, oral administration of 800 mg nafithromycin produced concentrations that were significantly higher in epithelial lining fluid (ELF) and alveolar macrophages (AMs) than concurrently in plasma throughout the 48-h period after 3 days of once-daily dosing. The ratios of ELF to plasma concentrations and of AM to plasma concentrations based on the mean AUC_{0-24} values were 13.8 and 527, respectively.

9.1.3 Phase II Studies of Nafithromycin

A Phase II prospective, multicentre, multinational, randomised (1:1:1), double-blind, comparative study was conducted to determine the efficacy, safety, tolerability and PK of oral nafithromycin (800 mg per os [PO] every 24 h (q24h) for 3 days and for 5 days) versus oral moxifloxacin (moxifloxacin 400 mg PO q24h for 7 days) for the treatment of adults with CABP.

A total of 224 subjects were analysed in the intent-to-treat (ITT) population, including 74 subjects in the nafithromycin 3-day arm, 73 subjects in the nafithromycin 5-day arm and 77 subjects in the moxifloxacin arm.

Overall, approximately 95% of subjects who were analysed completed the study and approximately 5% terminated early. The primary reasons for early study termination were withdrawal of consent (7 [63.6% of early terminations] subjects), loss to follow-up (3 [27.3%] subjects) and death (1 [9.1%] subject). The subject who died was in the moxifloxacin treatment arm.

Overall, 3 days of treatment with nafithromycin appears to be as efficacious as either 5 days of nafithromycin or 7 days of moxifloxacin. The clinical cure rates at test of cure (TOC) were similar between all 3 treatment arms.

For all pathogens, even at the highest MIC to study drug received, regardless of species and regardless of baseline MIC, the clinical response rates at Day 4 (92.2%) and TOC (94.6%) in the microbiological modified intent-to-treat (mMITT) population were high and similar among all treatment arms.

PK parameters of nafithromycin were generally comparable in the treatment arms. After administration on Day 3, nafithromycin plasma concentrations increased with peak concentrations observed in the 2-4 h and 6-10 h post-dose samples. At the end of the dosing interval (24-28 h after dose), plasma concentrations were comparable to the Day 3 pre-dose concentrations, confirming the achievement of steady-state conditions.

The results in this study suggest a safety profile of 3 days of nafithromycin consistent with that of moxifloxacin.

- There were few serious adverse events (SAEs) and the incidences were similar among treatment arms.
- One subject (moxifloxacin treatment arm) experienced a TEAE leading to premature discontinuation of the study drug.

- The most common preferred term (PT) was nausea occurring in 4.1%, 6.9% and 2.6% of subjects in the nafithromycin 3-day, nafithromycin 5-day and moxifloxacin treatment arms, respectively.
- TEAEs were mostly mild or moderate in all the treatment arms, with 2 subjects in each treatment arm having severe adverse events (AEs).
- No clinically meaningful differences were seen among the treatment arms in haematology, serum chemistry (excluding liver chemistry), vital signs or electrocardiograms (ECGs).
- Incidence of subjects with alanine aminotransferase (ALT) elevated beyond 3 times the upper limit of normal (ULN) was comparatively less in the nafithromycin 3-day arm (4.1%) than in the moxifloxacin arm (10.8%). Incidence of subjects with ALT elevated beyond 5 times the ULN was similar in the nafithromycin 3-day and moxifloxacin arms (1.4% and 1.4%, respectively). Total bilirubin was infrequently elevated in post-baseline samples across the treatment arms.

Compared with oral moxifloxacin 400 mg, oral nafithromycin 800 mg once a day for 3 days was generally safe, effective and well tolerated in this Phase II, randomised, double-blind, multinational study. Given the encouraging results of the preclinical and clinical studies, this Phase III clinical trial is planned to be conducted to determine the efficacy and safety of oral nafithromycin compared with oral moxifloxacin in the treatment of CABP in adults (≥ 18 years of age). The results of this trial are expected to aid the marketing authorisation application for nafithromycin in India and emerging markets.

9.2 RATIONALE

Drug resistance among respiratory tract infection (RTI) pathogens poses a serious and complex challenge in the effective management of CABP. Increasing resistance to ß-lactam and macrolide antibiotics of key causative pathogens of LRTIs, including pneumonia, is a worrisome trend. It also results in treatment failures and otherwise avoidable hospitalisations. Because LRTIs are one of the common causes of morbidity and mortality worldwide, newer therapeutic options are unquestionably needed to help reduce the disease burden.

Innovative structural modifications among newer ketolides provide them with several distinguishing features such as high-affinity binding to domain V and domain II of the 23S ribosomal ribonucleic acid (rRNA) target, in contrast to macrolides that bind only to domain V. Such dual-target binding by ketolides overcomes multiple macrolide resistance mechanisms such as *erm* gene-encoded methylases (macrolide-lincosamide-streptogramin B-type resistance, involving the methylation of the 23S rRNA target), point mutations within rRNA domain V and diverse mutations in ribosomal proteins L-4 and L-22. Additionally, newer ketolides such as nafithromycin are not susceptible to *mef* efflux pump-mediated resistance that affects the activity of macrolides against *S. pneumoniae* and *S. pyogenes*. Thus, these newer ketolides possess several features that not only confer activity against various resistotypes, irrespective of ß-lactam and macrolide susceptibility, but also minimise the risk of resistance emergence or induction of cross-resistance to other agents.

Nafithromycin (molecular formula $C_{42}H_{62}N_6O_{11}S$), Wockhardt's proprietary novel antibacterial agent of the lactone-ketolide class, looks promising to fulfil the unmet need of an effective antibacterial agent for the treatment of LRTIs

caused by MDR pathogens. In vitro MIC studies have shown that nafithromycin has excellent potency not only against macrolide- and ketolide-resistant strains of *S. pneumoniae* and Group A and B streptococci, but RTI pathogens such as *Haemophilus* species and *M. catarrhalis* and other atypical RTI pathogens such as *L. pneumophilia* (MIC range ≤0.004-0.06 μg/mL) (Dubois et al, 2016), *M. pneumoniae* (MIC_{90} ≤0.000125 μg/mL) (Waites et al, 2016) and *C. pneumophilia* (MIC range 0.03-2 μg/mL) (Kohlhoff and Hammerschlag, 2016). In vivo lung eradication and proof-of-concept studies have established superior pharmacodynamics of nafithromycin as compared to telithromycin under neutropenic conditions, particularly against *ermB*-expressing strains of streptococci. In a study involving contemporary clinical isolates collected from medical centres worldwide, nafithromycin was up to 8-fold more potent than telithromycin (Flamm et al, 2017). In vivo studies involving a 3-day treatment protocol provided a high extent of bacterial kill (≥ 2 $\log_{10}$ kill) as compared to the bacterial load at the initiation of treatment (refer to nafithromycin IB).

9.2.1 Rationale for Therapeutic Treatment With 800 mg of Nafithromycin Once Daily for 3 Days

Preclinical PK conducted in rodent and non-rodent species and Phase I clinical studies have shown optimal PK profile commensurate to the once-a-day dosing potential of nafithromycin. Administration of multiple ascending oral doses (600 mg, 800 mg and 1000 mg) of nafithromycin to humans was well tolerated.

Clinical doses of a novel respiratory antibiotic are justified based on the probability of attaining pharmacodynamic targets (PDTs) that have been identified in a murine neutropenic lung infection model. PDTs refer to the AUC/MIC ratio associated with 1 $\log_{10}$ kill of the baseline infecting pathogen in the lung. For nafithromycin, clinical doses have been justified taking into account both serum as well as ELF exposures. The magnitude of AUC/MIC was associated with 1 $\log_{10}$ kill employing murine serum and ELF PK against six strains of all *S. pneumoniae* (including penicillin- and macrolide-resistant) strains for nafithromycin. It was seen that a dose of 800 mg once daily would provide longer-term effective therapy for the management of pneumococcal infections as current global MICs for pneumococci are several fold lower than its PK/PD driven ability to provide the coverage of much higher MIC strain (refer to nafithromycin IB).

Using Phase I data, a population PK model was built and 2000 patient Monte Carlo Simulation was undertaken for the analysis of probability of target attainment (PTA). PTA analyses showed that a nafithromycin regimen of 800 mg once daily for 3 days would both provide > 90% PTA for contemporary clinical *S. pneumoniae* isolates taking into account plasma- and ELF-based PDTs. Attainment of high PTA suggests that a nafithromycin dose of 800 mg once daily administered over 3 days would be clinically effective against key contemporary RTI pathogens. Interestingly, the PTA was not adversely affected even in fed subjects, suggesting that nafithromycin efficacy would remain consistent for both fed and fasted subjects. Moreover, a nafithromycin dose of 800 mg once daily for 3 days would also provide coverage of penicillin-resistant, quinolone-resistant, azithromycin-resistant and telithromycin-non-susceptible *S. pneumoniae* isolates expressing a high level of MDR (data on file, 2018).

Nonclinical PK conducted in rodent and non-rodent species and Phase I clinical studies have shown an optimal PK profile favouring once-a-day dosing of nafithromycin. Metabolic studies have demonstrated a low cytochrome P450

(CYP)3A4 inhibitory potential for nafithromycin and its metabolites, suggesting that it would have a lower drug-drug interaction potential than other ketolides.

Preclinical toxicological evaluation of nafithromycin has overall demonstrated a promising safety profile. In these studies, the No Observable Adverse Effect Level exposure in rats and dogs was 3-4 times higher than the targeted therapeutic exposure. Hepatic safety of nafithromycin was found to be much more reassuring than that of telithromycin. In particular, non-clinical PK studies in rat and dog demonstrated a lower liver: serum partition ratio as compared to telithromycin (the partition ratio for nafithromycin was 48 and 61 at 15 and 30 mg/kg oral doses, respectively; in case of telithromycin, the ratio was 86 and 73 at 15 and 30 mg/kg).

Nafithromycin attained a geometric mean half-life of approximately 11 h in steady state at 800 mg/day in healthy human subjects (steady state was achieved on average within 3-4 days over the 600 to 1000 mg/day dose range). In addition, significant accumulation of nafithromycin in PMNs was observed. Furthermore, superior tissue penetration observed in rodents, particularly in the target organ (lung), is expected to drive superior pharmacodynamic activity, enabling a shorter duration of therapy of 3 to 5 days. A multiple dose study was conducted to compare plasma, ELF and AM concentrations of nafithromycin (800 mg administered once daily for 3 days) in healthy adult subjects. Each subject underwent 1 standardised bronchoscopy with BAL at 3, 6, 9, 12, 24 or 48 h after the third oral dose of nafithromycin. Concentrations of nafithromycin remained significantly higher in ELF and AM compared to those in plasma throughout the 48-h period after 3 days of once-daily dosing. The ratios of ELF to plasma concentrations and of AM to plasma concentrations based on the mean AUC_{0-24} values were 13.8 and 527, respectively (Rodvold et al, 2017).

Superior tissue penetration, particularly in the lung, justifies the short 3-day duration of nafithromycin therapy. Additionally, nafithromycin 800 mg once a day for 3 days was effective and well tolerated in a Phase II study.

In the intrapulmonary study, nafithromycin demonstrated therapeutically significant ELF concentrations even at 12, 24 and 48 h post after the third (last) dose (9.7, 4.1 and 1.6 µg/mL, respectively). These concentrations were above the MICs of nafithromycin against global collection of *S. pneumoniae* strains (MIC_{90} of 0.06 µg/mL for all *S. pneumoniae* tested, and 0.12 µg/mL for macrolide-and telithromycin-resistant *S. pneumoniae*). The sustained concentration in ELF and AM after just 3 oral doses suggest that nafithromycin has the potential to be an effective antibacterial agent for the treatment of LRTIs. Comprehensive coverage of RTI pathogens, superior target tissue concentrations, low drug-drug interaction potential and promising hepatic safety suggest that nafithromycin could be an effective and safe therapeutic option for the treatment of MDR RTIs.

9.2.2 Rationale for Moxifloxacin as Comparator

Moxifloxacin is a member of the fluoroquinolone class of antibiotics. It is active against most CABP pathogens including macrolide- and penicillin-resistant *S. pneumoniae*, Gram-negative bacteria, and atypical pathogens.

Empiric treatment of CABP with moxifloxacin, especially in subjects admitted to the hospital, is consistent with current US (Infectious Diseases Society of America [IDSA]/American Thoracic Society [ATS]) and European (The Task Force of the European Respiratory Society in collaboration with the European Society for Clinical

Microbiology and Infectious Diseases [ESCMID]) therapeutic guidelines. The recommended dose (IDSA/ATS) is 400 mg once daily for 7-14 days, and in this study, a 7-day regimen will be used.

9.3 POTENTIAL RISKS AND BENEFITS

9.3.1 Known Potential Risks

Subjects enrolled in this clinical study will have met the criteria for diagnosis of CABP. It is possible that nafithromycin will not prove to be a sufficiently effective treatment for CABP (i.e. not as effective as the comparator treatment).

The risk considerations for this study should encompass the known and potential risks for the development product nafithromycin and its components as well as the risks associated with other treatments that might be administered as described in this protocol. Other possible treatments include the marketed product moxifloxacin. As the risks for the marketed products are widely available in their respective prescribing information, such risks will not be discussed in this section.

The risks for nafithromycin have not been fully elucidated; however, the current risk profile for nafithromycin is described in the Investigator's Brochure (IB). In the Phase I studies of nafithromycin, the most frequently reported AEs were dysgeusia reported in 18 of 37 (49%) subjects and headache in 4 of 37 (11%) subjects.

In the Phase II study, the most common TEAE was nausea occurring in 4.1%, 6.9% and 2.6% of subjects in the nafithromycin 3-day, nafithromycin 5-day and moxifloxacin treatment arms, respectively. In a rat phrenic nerve diaphragm study, nafithromycin exacerbated myasthenia gravis; therefore, subjects with myasthenia gravis were excluded from the Phase II clinical trial. In this Phase III study, we plan to exclude myasthenia gravis subjects.

Based on the established safety and efficacy profiles of nafithromycin, it is expected that nafithromycin could provide an effective therapeutic option for treatment of CABP.

9.3.2 Known Potential Benefits

Subjects enrolled in this clinical study will have CABP that is of sufficient severity to require treatment with antibiotics. The potential benefit to subjects participating in this study is that they will receive effective antibiotic therapy for their infection. The potential benefit of the study is identification of a novel antibiotic product, which provides an effective treatment for CABP in this era of changing antibiotic resistance patterns.

Nafithromycin is expected to have the following advantages over older, available macrolides based on data from in vivo, in vitro, preclinical and clinical studies conducted till date:

1. Comprehensive coverage of key CABP pathogens, including atypical pathogens
2. Mechanism-based activity potential against macrolide- and ketolide-resistant pneumococci and Group A streptococci
3. Excellent target-organ-tissue concentration leading to:
    a. Potent activity against MDR pathogens in vivo
    b. Once-a-day dosing convenience

c. Potential for shorter duration of therapy

4. Minimal CYP inhibition leading to ease of co-administration of other drugs
5. Favourable hepatic safety potential due to favourable drug disposition resulting from lower accumulation in the liver

10 STUDY OBJECTIVES

10.1 PRIMARY OBJECTIVES

- To demonstrate that oral nafithromycin is non-inferior to oral moxifloxacin in the clinical response at Day 4 in the MITT analysis set
- To assess the overall safety of oral nafithromycin in the safety analysis set

10.2 SECONDARY OBJECTIVES

- To assess the clinical response at Day 4 in the mMITT and clinically evaluable (CE) analyses sets
- To assess the clinical outcome at End of Treatment (EOT) in the MITT, mMITT and CE analyses sets
- To assess the clinical outcome in the MITT, mMITT and CE analyses sets at the TOC Visit
- To assess re-admission to the hospital (or admission to the hospital if not previously hospitalised) for any reason prior to Follow-Up (FU) (Day 31 ± 4 days) in the MITT analysis set
- To determine the PK of oral nafithromycin (in PK analysis set)
- To assess by-pathogen clinical response at Day 4 and by-pathogen clinical outcome at TOC in the mMITT analysis set

11 STUDY DESIGN AND ENDPOINTS

11.1 DESCRIPTION OF THE STUDY DESIGN

This is a Phase III, prospective, multicentre, randomised, double-blind, comparative efficacy and safety study of oral nafithromycin versus moxifloxacin for the treatment of male and female adults (≥ 18 years of age) with CABP. Subjects providing informed consent and meeting eligibility criteria will be enrolled in the study and randomised in a 1:1 ratio, to either of the following 2 treatment arms:

- Nafithromycin 800 mg (two 400-mg tablets) PO q24h for 3 days; subjects will receive matching placebo PO q24h on Day 4 through EOT (2 tablets) and matching moxifloxacin placebo PO q24h on Day 1 through EOT (1 capsule) to maintain the blind (2 tablets and 1 capsule in total).
- Moxifloxacin 400 mg (1 capsule: over-encapsulated tablet) PO q24h for 7 days; subjects will receive matching nafithromycin placebo PO q24h on Day 1 through EOT (2 tablets), to maintain the blind (2 tablets and 1 capsule in total).

Study drugs should be administered approximately at the same time every day through the treatment period.

Baseline assessments for study eligibility will occur during the Screening visit, within 24 h before the administration of the first dose of the study drug. Block randomisation using an interactive voice/web response system (IXRS), stratified by Pneumonia Outcomes Research Team (PORT) Risk Class (II vs. III/IV) (Fine et al, 1997), will be used to assign subjects (1:1) to 1 of the 2 study treatment arms. Enrolment of PORT Risk Class II will be capped at 60% and enrolment of subjects with allowed prior systemic antibiotic use will be capped initially at 25% (subject to change during study conduct). Study Day 1 will be defined as the day when the study drug is first administered, and subsequent study days will be defined by the number of consecutive calendar days thereafter. Subjects may be treated in the study as inpatients or outpatients based on their clinical condition, at the discretion of the Investigator; however, only the oral study drug will be administered. Hospitalisation for convenience or social purposes may be permitted at the discretion of investigator. Such convenience or social admissions should be documented in the eCRF, however, will not qualify as admission due to Serious Adverse Event (SAE) will also not qualify for applicable secondary efficacy assessment. The investigator shall seek necessary approval for or notify ethics committee about convenience /social admissions, as per local requirements. Subjects will be assessed daily by the Investigator on Day 1 through Day 4, irrespective of the treatment setting (inpatient or outpatient). Primary efficacy variables will be assessed at Day 4.

Investigators will assess for clinical outcome at EOT and TOC (Day 15 ± 4 days). Best possible efforts should be made to conduct an in-person FU Visit on Day 31 ± 4 days. In the event that an in-person visit is not possible due to any reason, assessment may be conducted through a telephone contact for subjects who were considered to be Clinical Cures and had no AEs or clinically significant laboratory or ECG abnormalities noted at or after the TOC Visit. Such logistical reasons should be documented. For the subjects who had AEs or clinically significant laboratory or ECG abnormalities noted at or after the TOC Visit, an in-person FU Visit must be conducted.

11.1.1 Pharmacokinetic Sampling

Blood samples for PK analysis will be collected from the subjects on Day 1, Day 3 and Day 4 at sites where PK sampling is possible. Time points for PK sample collection will be as follows:

- A pre-dose PK sample will be collected within 10 min before dosing on Day 3.
- Post-dose PK samples will be collected at 2-4 h (Day 1 and Day 3) and 24-28 h (On Day 4, i.e. 24-28 h after Day 3 dose). Subjects who have been hospitalised are also required to have a post-dose PK sample at 6-10 h on Day 1 and Day 3.

11.2 STUDY ENDPOINTS

11.2.1 Efficacy Endpoints

11.2.1.1 Primary Endpoint

Clinical Response on Day 4 (MITT Analysis Set)

- Favourable Clinical Response: Alive and improvement of at least 1 level (e.g. severe to moderate, moderate to mild, mild to absent) in at least 2 CABP symptoms (dyspnoea, cough, production of purulent sputum or pleuritic chest pain) compared with that at the Baseline Visit, without worsening in any other of the 4 CABP symptoms. Severity of symptoms is based on a 4-point scale (absent, mild, moderate or severe).
- Unfavourable Clinical Response: No improvement of at least 1 level in at least 2 CABP symptoms compared with that at the Baseline Visit; or worsening in any of the 4 CABP symptoms compared with that at the Baseline Visit; or death from any cause at or before Day 4.
- Indeterminate: Study data are missing for the evaluation of efficacy at Day 4 for any reason, including loss to FU.

11.2.1.2 Secondary Endpoints

1. **Clinical Response on Day 4 (mMITT and CE Analyses Sets)**

- Favourable Clinical Response, Unfavourable Clinical Response and Indeterminate: Definitions are mentioned in Section 11.2.1.1.

2. **Clinical Outcome at EOT (MITT, mMITT and CE Analyses Sets)**

- Clinical Cure: Alive and signs and symptoms of CABP (dyspnoea, production of purulent sputum or pleuritic chest pain) are resolved or return to premorbid conditions and cough is improved such that further antibacterial therapy is not needed and the subject otherwise cannot be declared a clinical failure.
- Clinical Failure: The signs and symptoms of CABP (dyspnoea, production of purulent sputum or pleuritic chest pain) did not resolve or return to premorbid and/or cough worsened, such that non-study antibacterial therapy must be initiated for the treatment of CABP or death occurred prior to assessment or subject received alternative anti-microbial therapy for the treatment of CABP prior to this visit.
- Indeterminate: Study data are missing for the evaluation of efficacy at the assessment visit for any reason, including loss to FU.

3. Clinical Outcome at TOC (MITT, mMITT and CE Analyses Sets)

- Clinical Cure, Clinical Failure or Indeterminate: Definitions are as mentioned earlier in this section; failures at EOT will be carried forward to TOC.

4. Hospitalisation Before FU (MITT Analysis Set)

- Hospital re-admission for any reason between the Day 1 and the FU visits, if previously hospitalised and discharged or initial hospital admission for any reason between the Day 2 and the FU visits (Day 31 ± 4 days), if not previously hospitalised on Day 1.

5. By-Pathogen Clinical Response at Day 4 and Clinical Outcome at TOC (mMITT Analysis Set)

- The by-subject clinical response at Day 4 and clinical outcome at TOC defined above will be applied to each pathogen to obtain the:
  - By-pathogen Clinical Response at Day 4: Favourable, Unfavourable or Indeterminate (Definitions are as mentioned earlier)
  - By-pathogen Clinical Outcome at TOC: Clinical Cure, Clinical Failure or Indeterminate (Definitions are as mentioned earlier)

11.2.2 Safety Endpoints

- Incidence of subjects with TEAEs, SAEs and discontinuations due to TEAEs.

Incidence of subjects with potentially clinically significant (PCS) changes in safety laboratory parameters, vital signs and ECGs.

12 STUDY POPULATION

Approximately 488 adult subjects diagnosed with CABP will be enrolled in the study. Each subject will remain in the study for approximately 1 month. This will include a Screening/Baseline visit (within 24 h of randomisation), a 7-day oral treatment period and post-treatment assessments at TOC (Day 15 ± 4 days) and FU (Day 31 ± 4 days).

12.1 SUBJECT ELIGIBILITY, ENROLMENT, AND WITHDRAWAL

12.2 INCLUSION CRITERIA

To be eligible to participate in this study, the subject must meet all of the following criteria:

1. Male or female ≥ 18 years of age
2. Willing to participate in the study and provide written informed consent before any protocol-specific assessment is performed; consent from a legal guardian is not acceptable
3. Meet the following clinical criteria for CABP:
   a. Have at least TWO of the following symptoms (new or worsening):
      - Dyspnoea (shortness of breath)
      - Cough
      - Production of purulent sputum
      - Pleuritic chest pain

   b. Have at least TWO of the following vital sign abnormalities:
      - Fever or hypothermia documented by the Investigator (oral, rectal or tympanic temperature > 38.0°C [100.4°F] or < 36.0°C [95.5°F])
      - Hypotension, defined as systolic blood pressure < 90 mmHg
      - Tachycardia, defined as heart rate > 90 beats per minute
      - Tachypnoea, defined as respiratory rate > 20 breaths per minute

   c. Have at least ONE of the following clinical signs or laboratory abnormalities:
      - Hypoxaemia, defined as arterial oxygen saturation < 90% by pulse oximetry or partial pressure of arterial oxygen (PaO_2) < 60 mmHg by arterial blood gas (ABG)
      - Auscultatory findings on pulmonary examination consistent with bacterial pneumonia or pulmonary consolidation (e.g. crepitations, dullness on percussion, bronchial breath sounds or egophony)
      - Elevated total white blood cell (WBC) count (> 10,000 cells/mm^3) or leucopenia (WBC < 4,000 cells/mm^3)
      - Elevated immature neutrophils (> 15% band forms), regardless of total peripheral WBC count

   d. Radiographic evidence of CABP:
      - Radiographically confirmed pneumonia, i.e. new or progressive pulmonary infiltrate(s) on chest X-ray (CXR) or chest computed tomography (CT) scan consistent with acute bacterial pneumonia within 48 h before randomisation

   e. PORT score of 51 to 105 (PORT Risk Class of II, III or IV)

4. All females must have a negative urine or serum pregnancy test (ß-human chorionic gonadotropin [ß-HCG]) at Screening AND agree to the use of 1 of the following acceptable methods of contraception from Screening through TOC: surgical sterilisation (defined as bilateral oophorectomy or bilateral salpingectomy, but excluding bilateral tubal occlusion), post-menopausal women (defined by amenorrhea for at least 12 months following cessation of all exogenous hormonal treatments), barrier contraception (e.g. condom, intrauterine device), levonorgestrel intrauterine system (e.g. Mirena®), regular medroxyprogesterone injections (e.g. Depo-Provera®), sexual intercourse with only vasectomised partners or abstinence. (Note: sexual abstinence is considered a highly effective method only if defined as refraining from heterosexual intercourse during the entire period of risk associated with the study treatments. The reliability of sexual abstinence needs to be evaluated in relation to the duration of the clinical trial and the preferred and usual lifestyle of the subject.) Note that oral contraceptives should not be used as the sole method of birth control because the effect of nafithromycin on the efficacy of oral contraceptives has not yet been established; subjects who take oral contraceptives must also use 1 of the acceptable forms of birth control (listed above) from Screening through TOC.
5. All males must agree to use an acceptable barrier method of birth control (i.e. condom) with female partner(s) and must not donate sperm from Screening through TOC
6. Ability to ingest the intact oral study drug (e.g. able to swallow large capsules intact and no significant nausea, vomiting, diarrhoea or any other condition that might impair ingestion or absorption of the oral study drug)

12.3 EXCLUSION CRITERIA

Subjects who meet any of the following criteria will be excluded from participation in this study:

1. Subjects with any of the following confirmed or suspected types of pneumonia:
    - Aspiration pneumonia
    - Hospital-acquired bacterial pneumonia, defined as pneumonia with onset of clinical signs and symptoms after at least 48-h hospitalisation in an acute inpatient healthcare facility
    - Healthcare-associated bacterial pneumonia, defined as pneumonia acquired in a long-term care or subacute healthcare facility (e.g. nursing home) or pneumonia with onset after recent hospital discharge (within 90 days of current admission and previously hospitalised for ≥ 48 h)
    - Ventilator-associated bacterial pneumonia, defined as pneumonia with onset of clinical signs and symptoms after at least 48 h of endotracheal intubation
    - Pneumonia that may be caused by pathogen(s) resistant to any study drug (nafithromycin or moxifloxacin), including viral, mycobacterial or fungal pneumonia (e.g., *Pneumocystis jiroveci* pneumonia, active pulmonary tuberculosis)
    - Post-obstructive pneumonia
    - Pneumonia associated with cystic fibrosis, bronchiectasis or any other chronic pulmonary disease

2. Suspected or confirmed pleural empyema (a parapneumonic pleural effusion is not an exclusion criterion) or lung abscess
3. Suspected or confirmed non-infectious causes of pulmonary infiltrates (e.g. pulmonary embolism, hypersensitivity pneumonia, congestive heart failure)
4. Receipt of 1 or more dose(s) of a potentially effective systemic antibacterial treatment for treatment of the current CABP within 72 h before randomisation except if the prior therapy is a single dose of a short-acting antibacterial agent (Appendix I, Section 23.1 for allowable prior antibiotics); subjects requiring concomitant adjunctive or additional potentially effective systemic antibacterial treatment for management of CABP
5. Evidence of significant immunologic disease determined by any of the following:
   - Current or anticipated neutropenia defined as < 500 neutrophils/mm^3
   - Known history or diagnosis of human immunodeficiency virus (HIV) infection by serology. In the event that the results of HIV serology are not available promptly, the total white blood cell count is ≥ 500 cells per cubic millimetre, and all the other eligibility criteria are met, the subject can be randomised and given the first dose of study treatment for management of CABP. If the HIV serology is positive, the subject can continue in the study if the Investigator deems it will not interfere with optimal study participation (e.g. evaluation of study drug efficacy, determination of safety, or completion of the expected course of treatment) and medical management of the underlying CABP. Investigator should discuss all positive cases with the Medical Monitor.
   - History of heart, lung or kidney transplant
   - Receipt of cancer chemotherapy, radiotherapy or potent, non-corticosteroid immunosuppressant drugs (e.g. cyclosporine, azathioprine, tacrolimus, immune-modulating monoclonal antibody therapy) within the past 3 months or receipt of corticosteroids equivalent to or greater than 40 mg of prednisone per day for more than 14 days in the 30 days before randomisation
6. Known or suspected primary or metastatic neoplastic lung disease, bronchiectasis, cystic fibrosis, bronchial obstruction, chronic neurological disorder preventing clearance of pulmonary secretions or severe chronic obstructive pulmonary disease (severe chronic obstructive pulmonary disease is defined as known [before Screening] ratio of forced expiratory volume in 1 second [FEV_1] to forced vital capacity [FVC] < 0.70 and FEV_1 < 50% normal); note that pulmonary function tests are not required at Screening
7. Compromised hepatic or renal function, including but not limited to the following: clinical evidence of end-stage liver disease (e.g. ascites, hepatic encephalopathy), screening serum total bilirubin > 2 times the ULN (unless associated with an elevated indirect bilirubin typical of Gilbert syndrome), aspartate aminotransferase (AST) or ALT ≥ 3 times the ULN, serum creatinine > 2.0 mg/dL, creatinine clearance < 50 mL/min or blood urea nitrogen > 30 mg/dL; other clinically significant abnormal laboratory findings should be discussed with the Medical Monitor before the subject's entry
8. History of *Clostridium difficile*-associated disease within 6 months before enrolment

9. History of hypersensitivity, known contraindication (e.g. lactose intolerance, lactase deficiency or glucose-galactose malabsorption) or allergic reaction (e.g. anaphylaxis, urticaria, other significant reaction) to any ketolide or fluoroquinolone antibiotic
10. Current second- or third-degree atrioventricular block or sick sinus syndrome, uncontrolled atrial fibrillation, severe or unstable angina, congestive heart failure, myocardial infarction within 3 months before the Screening visit, clinically significant ECG abnormalities including QT interval corrected for heart rate using Fridericia's formula (QTcF) > 450 ms (males) or > 470 ms (females) or requirement for medications known to cause QT prolongation
11. Prior (within 14 days before randomisation) or concomitant use of CYP liver enzyme inducers (e.g., phenobarbital, carbamazepine, griseofulvin, sulfonylureas, phenytoin or rifampin)
12. Current peripheral neuropathy or myasthenia gravis
13. Known or suspected seizure disorder or other central nervous system disorders that may predispose the subject to seizures or lower the seizure threshold
14. Nursing mothers or pregnant females
15. Subjects who received any experimental drug within 30 days before enrolment
16. Require admission to an intensive care unit for any reason, life expectancy of < 2 months or any concomitant condition that in the opinion of the Investigator is likely to interfere with evaluation of the response of the infection under study, determination of AEs or completion of the expected course of treatment
17. History of tendon disorders
18. A: Active or suspected pulmonary tuberculosis (TB).

    B: At Indian sites, the subject not agreeing to diagnostic evaluation of tuberculosis by Xpert TB test (using GeneXpert).[#]

[#]In the event that the results of Xpert TB test are not available promptly and all the other eligibility criteria are met, the subject can be randomized and given the first dose of study treatment for management of CABP. The subject will be discontinued from study therapy prior to the second dose if the results of Xpert TB test suggest "detection" of Mycobacterium tuberculosis complex (MTBC) and is indicative of active pulmonary tuberculosis. The investigator should consider standard of care at their discretion for further management of such subjects.

12.4 SCREEN FAILURES

Screen failures are defined as participants who consent to participate in the clinical trial but are not subsequently randomly assigned to the study intervention or enrolled in the study. A minimal set of screen failure information is required to ensure transparent reporting of screen failure participants, to meet the Consolidated Standards of Reporting Trials (CONSORT) publishing requirements and to respond to queries from regulatory authorities. Minimal information includes demography, reasons for screen failures and any SAE, which will maintained as part

of study logs. Potential subjects who do not meet entrance criteria may, as appropriate, be rescreened and undergo repeat baseline assessments within 72 h of initial screening for possible enrolment into the study.

12.5 STRATEGIES FOR RECRUITMENT AND RETENTION

Subjects diagnosed with CABP visiting the outpatient department or subjects hospitalised for CABP in secondary or tertiary care hospitals will be considered for enrolment in this study. All attempts will be made to retain the enrolled subjects.

12.6 CRITERIA FOR PREMATURE DISCONTINUATION OF STUDY DRUG

Subjects should be encouraged to complete all study assessments. However, subjects may discontinue study drug or withdraw consent to participate in this study at any time without penalty or loss of benefits to which the subject is otherwise entitled.

12.6.1 Premature Discontinuation from Study Drug Administration

12.6.1.1 Discontinuations Due to Safety

Possible reasons for premature discontinuation from study drug administration due to safety include, but are not limited to, the following:

- Occurrence of an AE that, in the opinion of the Investigator, warrants the subject's permanent discontinuation from study drug administration
- Meets Hy's law criteria, defined by at least 3-fold elevations of ALT or AST above the ULN, elevation of serum total bilirubin to > 2 times ULN without elevated serum alkaline phosphatase and no other disease or condition can be found to explain the liver test abnormalities
- Known pregnancy or breastfeeding during the study drug administration period. Female subjects whose pregnancy test is positive after baseline must be followed through the immediate post-natal period or until termination of the pregnancy; study centre personnel must report every pregnancy as soon as possible (within 24 h of learning of the pregnancy, as described in Section 18.4.3)

Assessments and Procedures: Subjects who are prematurely discontinued from study drug administration (i.e., before the anticipated full course of study drug required for effective treatment of the CABP) for safety reasons should continue to undergo study assessments at every study visit (Section14). If a subject is discontinued from study drug on Day 3 or Day 4, an attempt should be made to collect any remaining PK blood samples scheduled for that day.

Clinical Outcome Assessment: Subjects who are prematurely discontinued from study drug administration for safety reasons and for whom further antibacterial therapy is not required for treatment of the primary infection (i.e. the CABP has resolved completely or improved to the point where no further antibacterial therapy is necessary), may be assessed as a clinical cure at the EOT and TOC visits. If EOT occurs prior to Day 4, the subject must return for Day 4 CABP symptom severity assessment.

Subjects prematurely discontinued from any study drug for safety reasons and who require further antibacterial therapy for the CABP should be assessed as a clinical failure on the day of discontinuation and automatically assigned an outcome of clinical failure at the next outcome evaluation time point.

12.6.1.2 Discontinuations Due to Insufficient Therapeutic Effect

Possible reasons for discontinuation from study drug due to insufficient therapeutic effect include, but are not limited to, the following:

- Clinical worsening: Subjects who show systemic or local signs of clinical worsening may be prematurely discontinued from study drug administration at any time. If the Investigator deems the benefit-to-risk ratio of study drug continuance acceptable, study drug administration of at least 48 h is encouraged before discontinuation from study drug therapy.
- Lack of clinical progress: For subjects who are stable, yet do not show signs of improvement, the Investigator is encouraged to continue study drug therapy for at least 48 h before such subjects are considered clinical failures and prematurely discontinued from study drug therapy.

Assessments and Procedures: Subjects who are prematurely discontinued from study drug due to insufficient therapeutic effect should have EOT assessments conducted (Section 14.8) on the day of discontinuation and undergo safety assessments at the TOC Visit (Section 14.9). Prematurely discontinued subjects should also be encouraged to attend the FU Visit. If a subject is discontinued prior to Day 4, every attempt should be made to assess Day 4 CABP symptom severity. If a subject is discontinued from study drug on Day 3 or Day 4, an attempt should be made to collect any remaining PK blood samples scheduled for that day. If a subject is discontinued from study drug administration due to insufficient therapeutic effect and is switched to an alternative antibiotic, that therapy should be documented.

Clinical Outcome Assessment: Subjects who are prematurely discontinued from study drug administration due to insufficient therapeutic effect should be assessed as clinical failure on the day of discontinuation. Subjects prematurely discontinued from study drug administration due to insufficient therapeutic effect will be automatically assigned an outcome of clinical failure at the next outcome evaluation time point.

12.6.2 Guidance to Investigators on When to End Study Drug Therapy

If additional antibacterial therapy is required for the index CABP (e.g. progressively worsening CABP between Day 1 and EOT or gradually improving CABP that requires additional antibacterial therapy beyond 7 days), then study drug should be discontinued; the subject will be deemed a clinical failure on the day of study drug discontinuation, and other antibacterial therapy may be started at the discretion of the Investigator.

In cases of premature discontinuation of study drug, subjects should not be discontinued from the study itself; subjects should remain in the study and undergo all scheduled assessments at EOT, TOC and FU.

The Investigator may use culture and susceptibility results from the local microbiology laboratory to help guide therapy; however, decisions to continue or discontinue study drug should be based on clinical response rather than susceptibility results (as nafithromycin susceptibility testing is not available at the local site). If the index CABP is

caused by a microorganism that is not susceptible to fluoroquinolones, macrolides or ketolides in vitro, the decision to continue or discontinue study treatment should be based on the subject's clinical course and the Investigator's clinical judgement. These cases should be discussed with the Medical Monitor prior to prematurely discontinuing study drug and the rationale for this decision should be recorded in the source documents.

13 STUDY TREATMENT

Subjects providing informed consent and meeting all study eligibility criteria will be enrolled in the study.

- Nafithromycin 800 mg (two 400 mg tablets) PO q24h for 3 days; subjects will receive matching placebo PO q24h on Day 4 through EOT (2 tablets) and matching moxifloxacin placebo PO q24h on Day 1 through EOT (1 t capsule) to maintain the blind (2 tablets and 1 capsule in total)
- Moxifloxacin 400 mg (1 capsule: Over-encapsulated tablet) PO q24h for 7days; subjects will receive matching nafithromycin placebo PO q24h on Day 1 through EOT (2 tablets) to maintain the blind (2 tablets and 1 capsule in total)

13.1 STUDY AGENTS AND CONTROL DESCRIPTION

13.1.1 Acquisition

The study drugs will be supplied by the Sponsor across all study sites.

13.1.2 Formulation, Appearance, Packaging and Labelling

- Oral nafithromycin 400-mg and corresponding clinical placebo tablets white, oval shaped, biconvex film coated tablet, debossed with 'W755' on one side and plain on other side (refer to nafithromycin IB).
- Oral moxifloxacin 400 mg tablets are dull red film-coated tablets with an oblong, convex shape with facet. Moxifloxacin tablets will be over-encapsulated for maintaining the blind in this study. The over-encapsulated moxifloxacin and corresponding clinical placebo capsules are opaque orange capsules containing Moxifloxacin 400 mg tablets or inert ingredients respectively.

13.1.3 Product Storage and Stability

- Storage and dispensing of nafithromycin tablets and corresponding clinical placebo tablets can be undertaken at room temperature (below 25°C) in its packaging (while protected from moisture). Detailed instructions regarding storage are presented in the IB.
- Moxifloxacin tablets and corresponding clinical placebo capsules should be stored below 25°C in well-closed containers.

13.1.4 Preparation

No special preparation is required for administration of the study drug.

13.1.5 Dosing and Administration

13.1.5.1 Nafithromycin

- Strength and pharmaceutical dosage form: 400 mg tablet
- Dose: 800 mg once daily (2 tablets of 400 mg each) for 3 days; subjects will receive matching placebo PO q24h on Day 4 through EOT (2 tablets) and matching moxifloxacin placebo PO q24h on Day 1 through EOT (1 capsule) to maintain the blind (2 tablets and 1 capsule in total)
- Route of administration: Oral

13.1.5.2 Moxifloxacin

- Strength and pharmaceutical dosage form: 400 mg tablet
- Dose: 400 mg daily for 7 days; subjects will receive matching nafithromycin placebo PO q24h on Day 1 through EOT (2 tablets) to maintain the blind (2 tablets and 1 capsule in total)
- Route of administration: Oral

13.1.6 Route of Administration

The route of administration for all study drugs is oral.

13.1.7 Starting Dose and Dose Escalation Schedule

Dose escalation is not applicable in this study.

13.1.8 Dose Adjustments/Modifications/Delays

There will be no dose adjustments in this study.

13.1.9 Duration of Therapy

Duration of therapy is described in Section 11.1.

13.2 CONCOMITANT THERAPY

All concomitant medications taken during study participation will be recorded in the eCRFs. Medications to be reported in the eCRF are concomitant prescription medications, over-the-counter medications and non-prescription medications. For this protocol, a prescription medication is defined as a medication that can be prescribed only by authorised/licensed clinician.

13.3 PROHIBITED MEDICATIONS

Subjects with prior (within 14 days before randomisation) treatment with or concomitant use of CYP liver enzyme inducers (e.g. phenobarbital, carbamazepine, griseofulvin, sulfonylureas, phenytoin or rifampin), cancer chemotherapy, radiotherapy or potent, non-corticosteroid immunosuppressant drugs (e.g. cyclosporine, azathioprine, tacrolimus or immune-modulating monoclonal antibody therapy) within the past 3 months or have received corticosteroids equivalent to or greater than 40 mg of prednisone per day for more than 14 days in the 30 days before randomisation and subjects who received any experimental drug within 30 days before enrolment will be excluded from the study. Please refer to the moxifloxacin Summary of Product Characteristics (SmPC) for the list of prohibited medications.

13.4 BLINDING AND UNBLINDING PROCEDURES

This study will be double-blinded with regard to study drug treatment. After written informed consent has been obtained and eligibility established, the study centre's pharmacist/designee will obtain the randomisation code using IXRS. The IXRS system will also confirm the study drug assignment including the unique identification number(s) of the kits to be dispensed to the subject. The pharmacist/designee will be responsible for maintaining accountability and dispensing the oral study drug according to the handling instructions. Study centre personnel will remain blinded to the identity of study drug until the database has been locked and the study has been unblinded. In the case

of a medical emergency requiring the Investigator to know the identity of the oral study drug, the Investigator will follow the procedures outlined below. Individual treatment codes, indicating the treatment for each randomised subject, will be available to the Investigators or pharmacists through IXRS. IXRS procedures will be described in the IXRS user manual that will be provided to each centre. To maintain Investigator blinding, the treatment code should not be broken except in medical emergencies when the appropriate management of the subject requires knowledge of the treatment randomisation. In such a case, the subject should receive all appropriate medical care. Prior to any unblinding, the Investigator should contact the Sponsor Medical Monitor to discuss options. The unblinding procedure will be done through the IXRS system. As soon as possible and without revealing the subject's study drug assignment (unless important to the safety of subjects remaining in the study), the Investigator must notify the Sponsor if the blind is broken for any reason and the Investigator is unable to contact the Sponsor prior to unblinding. The Investigator will record in the source documentation the date and reason for revealing the blinded treatment assignment for that subject; the treatment assignment itself should not be entered into source documentation. The Sponsor may break the code for SAEs that are unexpected and are believed to be causally related to study drug and that potentially require expedited reporting to regulatory authorities. In such cases, the minimum number of Sponsor personnel will be unblinded. Treatment codes will not be broken for the planned analyses of data until all decisions on the evaluability of the data from each individual subject have been made and documented and databases have been locked.

14 STUDY PROCEDURES AND CONDUCT

The Schedule of Assessments is provided in Table 1.

14.1 SCREENING VISIT

Written and signed informed consent must be obtained before any protocol assessment is performed.

Screening visit procedures must be completed within 24 h before randomisation to determine study eligibility. Potential subjects who do not meet eligibility criteria may, as appropriate, be rescreened and undergo repeat baseline assessments within 72 h of initial screening for possible enrolment in the study.

Local laboratory results will be used to determine subject eligibility for study enrolment. Any protocol-required eligibility laboratory or radiological evaluations already performed as part of the subject's regular medical care or site's standard of care within 24 h (laboratory) and 48 h (radiology) before randomisation do not have to be repeated to determine subject eligibility. In addition, laboratory assessments must be sent to the central laboratory as part of baseline safety assessments.

If local laboratory results are not confirmed by central laboratory results, the subject should not be automatically withdrawn from the study or the study drug. In such cases, the subject should be assessed for safety and the Medical Monitor must be contacted to confirm subject eligibility to remain on study.

14.1.1 Clinical Assessments

- Assess eligibility criteria in detail
- Record significant medical and surgical history, including all active conditions and ongoing comorbidities (at least for 5 years before the Screening Visit)
- Record all prior medications that have been administered within 14 days before the date of signing the informed consent
- Record vital signs including body temperature (oral, rectal or tympanic), blood pressure (sitting position), pulse rate, respiratory rate and pulse oximetry; the highest recorded daily temperature should be recorded and attempts should be made to measure temperatures using the same methodology throughout the study
- Perform a complete physical examination, including general appearance, the skin, eyes, ears, nose, throat, lungs, heart, abdomen, back, extremities, lymph nodes, vascular and neurological examinations
- Assess present as well as premorbid (approximately 7 days prior to onset of current CABP symptoms) CABP symptom severity (Appendix III, Section 23.3)
- Obtain a 12-lead ECG
- Identify, assess and record any AEs (since the time of informed consent)

14.1.2 Radiographic Evaluation

Obtain a CXR or chest CT scan for all subjects at Screening. If a CXR is performed, posteroanterior and lateral views are required (unless it is medically not feasible) along with confirmation of new or progressive pulmonary infiltrates consistent with CABP. Anteroposterior (i.e. portable) views are also acceptable if performing

posteroanterior and lateral views is medically not feasible. Chest radiography may be obtained as part of routine, non-study evaluation of a subject presenting with signs and symptoms of CABP and therefore may be performed in some circumstances before informed consent is obtained for participation in this study; radiological evaluations already performed as part of the subject's regular medical care or site's standard of care within 48 h before randomisation do not have to be repeated to determine subject eligibility.

Radiologic evaluation(s) will be performed locally and interpreted by appropriately qualified personnel, who are certified or licensed to interpret chest radiographs according to applicable regional requirements, and the evaluations will be reviewed by the Investigator or qualified personnel during the Screening visit; the conclusions of the Investigator's review will be the basis for subject inclusion. The written radiography report (i.e. formal interpretation of the radiographic image or film) should be included in the source documents along with X-ray films and images.

14.1.3 Local Laboratory Assessments

- Obtain serum aminotransferase (ALT, AST) and total bilirubin levels, coagulation profile, serum creatinine and blood urea nitrogen (or urea), peripheral WBC count, absolute neutrophil count, immature neutrophil percentage and HIV serology to determine eligibility. ABG is recommended, but not required, to measure blood pH and PaO_2 for calculating the PORT score.
- Obtain serum or urine sample for pregnancy test (ß-HCG) in all females and ensure that the test is negative before randomisation.
- Obtain a urine dipstick test: a standard urine dipstick includes detection of specific gravity, pH, leucocytes, blood (haemoglobin), nitrite, ketones, bilirubin, urobilinogen, protein and glucose. If any result is abnormal (e.g. not negative) and deemed clinically significant by the Investigator, a urinalysis will be sent to the central laboratory.

14.1.4 PORT Score

Calculate the PORT score using local laboratory and radiographic results (Appendix II, Section 23.2). As part of the Inclusion Criteria of this study, subjects must have a PORT score between 51 and 105 (PORT Risk Class of II, III or IV) at randomisation. For subjects with an optional ABG at Screening, no points will be added for pH or PaO_2; however, oxygen saturation results can be used in place of PaO_2 (Appendix II, Section 23.2).

14.1.5 Central Laboratory Assessments

- Obtain complete blood count (CBC), chemistry panel, urinalysis (only if local urine dipstick results are abnormal and deemed clinically significant by the Investigator) and serum ß-HCG test (Appendix IV, Section 23.4)
- Collect blood for serology testing for *L. pneumophila*, *M. pneumoniae* and *C. pneumoniae* by the central laboratory

14.1.6 Microbiological Assessments

- Attempt to collect an adequate quality expectorated or induced sputum or other respiratory specimen reflecting fluid from the lower respiratory tract from every subject (e.g., respiratory fluid obtained by BAL or bronchoscopy; pleural fluid obtained by thoracentesis; or expectorated or induced sputum meeting adequacy criteria) and submit the specimen to the microbiology laboratory for Gram staining and culture (see Study Specific Microbiology Laboratory Procedures Manual)
- For the subjects being enrolled at Indian sites, an expectorated sputum sample will be collected for diagnostic evaluation of tuberculosis by Xpert TB test (using GeneXpert) in addition to collection of expectorated sputum or other deep respiratory sample for microbiological assessments as mentioned above. Collection of two sputum/respiratory samples, on the day of screening, could be scheduled at the discretion of investigator based on convenience of the subject.
- Obtain blood (1 aerobic bottle and 1 anaerobic bottle from 2 separate venipuncture sites, a total of 4 bottles collected) sample for culture
- Collect a urine specimen to test for the presence of *L. pneumophila* and *S. pneumoniae* antigens. The details of testing will be provided in the laboratory manual.
- Randomise the subject using IXRS after verifying that the subject meets all study inclusion criteria (Section 12.2) and no exclusion criteria (Section 12.3).

14.2 STUDY DAY 1

Day 1 is the first calendar day of study drug administration. Subsequent study days are consecutive calendar days. If feasible, screening and randomisation procedures (Screening visit and Day 1) can be performed on the same day. Standard of care laboratory and radiological data from within 24 h (laboratory) and 48 h (radiology) before randomisation, respectively, can be used as Screening visit procedures. Regardless of whether the Screening visit and Day 1 occur on different calendar days or on the same day, verify all Inclusion Criteria and Exclusion Criteria before randomisation. All Day 1 procedures described below and in Table 1 are to be conducted after the administration of first dose and before administration of the second dose of the study drug. Administer the study drug per the schedule in Section 11.1.

14.2.1 Clinical Assessments

- Record 1 set of vital signs, including body temperature (highest recorded daily oral, rectal or tympanic temperature), blood pressure, pulse rate, respiratory rate and pulse oximetry
- Perform a complete physical examination
- Assess CABP symptom severity (Appendix III, Section 23.3)
- Identify, assess and record any AEs
- Record any concomitant medications

14.2.2 Pharmacokinetic Assessments

- Blood samples for PK analyses will be taken from all subjects at sites where PK sampling is possible on Day 1 (as detailed in Section 15)

14.3 STUDY DAY 2

- Administer the study drug per the schedule in Section 11.1

14.3.1 Clinical Assessments

- Record 1 set of vital signs, including body temperature (highest recorded daily oral, rectal or tympanic temperature), blood pressure, pulse rate, respiratory rate and pulse oximetry
- Perform a complete physical examination
- Assess CABP symptom severity (Appendix III, Section 23.3)
- Identify, assess and record any AEs
- Record any concomitant medications

14.3.2 Microbiological Assessments

- If baseline blood cultures are positive, repeated post-baseline blood cultures should be collected until a negative result is obtained. Sites may wait until confirmation of results of previous cultures to collect further samples. Repeated respiratory specimens should be obtained only if clinically indicated

14.4 STUDY DAY 3

- Administer the study drug per the schedule in Section 11.1

14.4.1 Clinical Assessments

- Record 1 set of vital signs, including body temperature (highest recorded daily oral, rectal or tympanic temperature), blood pressure, pulse rate, respiratory rate and pulse oximetry
- Perform a complete physical examination
- Assess CABP symptom severity (Appendix III, Section 23.3)
- Obtain a 12-lead ECG
- Identify, assess and record any AEs
- Record any concomitant medications

14.4.2 Central/Local Laboratory Assessments

- Obtain haematology and chemistry panel (Appendix IV, Section 23.4) for central laboratory assessment
- Conduct coagulation profile at local laboratory

14.4.3 Pharmacokinetic Assessments

- Blood samples for PK analyses will be taken from all subjects at sites where PK sampling is possible on Day 3 (as detailed in Section 15)

14.4.4 Microbiological Assessments

- If baseline blood cultures are positive, repeated post-baseline blood cultures should be collected until a negative result is obtained. Sites may wait until confirmation of results of previous cultures to collect further samples. Repeated respiratory specimens should be obtained only if clinically indicated

14.5 STUDY DAY 4

- Administer the study drug per the schedule in Section 11.1

14.5.1 Clinical Assessments

- Record 1 set of vital signs, including body temperature (highest recorded daily oral, rectal or tympanic temperature), blood pressure, pulse rate, respiratory rate and pulse oximetry
- Perform a complete physical examination
- Identify, assess and record any AEs
- Record any concomitant medications
- Assess CABP symptom severity (Appendix III, Section 23.3). CABP symptom severity information entered into the eCRF will be used to programmatically determine clinical response at Day 4 (Section 14.5); note this is not an Investigator-determined clinical outcome

14.5.2 Pharmacokinetic Assessments

- Blood samples for PK analyses will be taken from all subjects at sites where PK sampling is possible on Day 4 (as detailed in Section 15)

14.5.3 Microbiological Assessments

- If baseline blood cultures are positive, repeated post-baseline blood cultures should be collected until a negative result is obtained. Sites may wait until confirmation of results of previous cultures to collect further samples. Repeated respiratory specimens should be obtained only if clinically indicated

14.6 STUDY DAY 5

Best possible efforts should be made to conduct an in-person visit. In the event that an in-person visit is not possible due to any reason, assessment may be conducted through a telephone contact.

- Administer the study drug per the schedule in Section 11.1

14.6.1 Clinical Assessments

- Identify, assess and record any AEs
- Record any concomitant medications

14.6.2 Microbiological Assessments

- If baseline blood cultures are positive, repeated post-baseline blood cultures should be collected until a negative result is obtained. Sites may wait until confirmation of results of previous cultures to collect further samples. Repeated respiratory specimens should be obtained only if clinically indicated

14.7 STUDY DAY 6

Best possible efforts should be made to conduct an in-person visit. In the event that an in-person visit is not possible due to any reason, assessment may be conducted through a telephone contact.

- Administer the study drug per the schedule in Section 11.1

14.7.1 Clinical Assessments

- Identify, assess and record any AEs
- Record any concomitant medications

14.7.2 Microbiological Assessments

- If baseline blood cultures are positive, repeated post-baseline blood cultures should be collected until a negative result is obtained. Sites may wait until confirmation of results of previous cultures to collect further samples. Repeated respiratory specimens should be obtained only if clinically indicated

14.8 STUDY DAY 7 (+ 2 DAYS) (END OF TREATMENT)

- Perform visit specific assessments at any time during Day 7 after the final dose of the study drug or within 2 days after Day 7
- Administer the study drug per the schedule in Section 11.1

14.8.1 Clinical Assessments

- Record 1 set of vital signs, including body temperature (highest recorded daily oral, rectal or tympanic temperature), blood pressure, pulse rate, respiratory rate and pulse oximetry
- Perform a complete physical examination
- Assess CABP symptom severity (Appendix III, Section 23.3)
- Obtain a 12-lead ECG
- Identify, assess and record any AEs
- Record any concomitant medications
- Conduct clinical outcome assessment

14.8.2 Central/Local Laboratory Assessments

- Obtain haematology, coagulation and chemistry panel (Appendix IV, Section 23.4) for central laboratory assessment
- Conduct coagulation profile at local laboratory

14.8.3 Microbiological Assessments

- If baseline blood cultures are positive, repeated post-baseline blood cultures should be collected until a negative result is obtained. Sites may wait until confirmation of results of previous cultures to collect further samples. Repeated respiratory specimens should be obtained only if clinically indicated

14.9 STUDY DAY 15 (± 4 DAYS) (TEST-OF-CURE)

- Perform TOC assessments on Day 15 ± 4 days

14.9.1 Clinical Assessments

- Record 1 set of vital signs, including body temperature (highest recorded daily oral, rectal or tympanic temperature), blood pressure, pulse rate, respiratory rate and pulse oximetry
- Perform a complete physical examination
- Assess CABP symptom severity (Appendix III, Section 23.3)
- Obtain a 12-lead ECG only if prior study ECG(s) showed any clinically significant abnormality
- Identify, assess and record any AEs
- Record any concomitant medications
- Conduct clinical outcome assessment

14.9.2 Central Laboratory Assessments

- Obtain CBC, chemistry panel and serum ß-HCG test (Appendix IV, Section 23.4) for central laboratory assessment
- Conduct coagulation profile at local laboratory
- Collect blood for serology testing for *L. pneumophila*, *M. pneumoniae* and *C. pneumoniae* by the central laboratory

14.9.3 Microbiological Assessments

- If baseline blood cultures are positive, repeated post-baseline blood cultures should be collected until a negative result is obtained. Sites may wait until confirmation of results of previous cultures to collect further samples. Repeated respiratory specimens should be obtained only if clinically indicated

14.10 STUDY DAY 31 (± 4 DAYS) (FOLLOW-UP VISIT)

Best possible efforts should be made to conduct an in-person FU Visit on Day 31 ± 4 days. In the event that an in-person visit is not possible due to any reason, assessment may be conducted through a telephone contact for subjects who were considered to be Clinical Cures and had no AEs or clinically significant laboratory or ECG abnormalities noted at or after the TOC Visit. Such logistical reasons should be documented. For the subjects who had AEs or clinically significant laboratory or ECG abnormalities noted at or after the TOC Visit, an in-person follow up visit must be conducted.

14.10.1 Clinical Assessments

- Assess for clinical relapse or recurrence of CABP, including new hospitalisation for any reason within 30 days from initiation of treatment (a secondary endpoint, see Section 17.1.2)
- Obtain a 12-lead ECG only if prior ECG(s) showed any clinically significant abnormality
- Identify, assess and record any AEs

- Record any concomitant medications

14.10.2 Central/Local Laboratory Assessments

- Obtain CBC, coagulation and/or chemistry panel for local laboratory assessment only if prior study laboratory results showed any clinically significant abnormality (Appendix IV, Section 23.4); do not repeat previously normal (or abnormal but not clinically significant) laboratory tests
- Collect blood for serology testing at central laboratory for *L. pneumophila, M. pneumoniae* and *C. pneumoniae*

14.10.3 Microbiological Assessments

- If baseline blood cultures are positive, repeated post-baseline blood cultures should be collected until a negative result is obtained. Sites may wait until confirmation of results of previous cultures to collect further samples. Obtain repeated respiratory specimens only if clinically indicated, e.g., in case of clinical relapse or recurrence of CABP

14.11 UNSCHEDULED VISIT

Interim visits may be conducted at participant request or study Investigator request, at any time during the study. Interim visits include any unscheduled visit taking place within a fixed amount of time from the previous visit or before the next scheduled visit. Clinical reasons may include FU care for particular infections or FU care for responses to study treatments. All visits will be documented in participants' study records and on eCRFs.

15 PHARMACOKINETIC ASSESSMENTS

The PK data acquisition and analysis strategy entails the use of a sparse PK sampling schedule. PK samples will be obtained from all subjects at sites where PK sampling is possible (for which a study centre has been identified by the Sponsor as eligible to perform PK assessments). PK sample handling and shipping procedures are described in the PK Sample Handling and Shipping Manual.

15.1 PHARMACOKINETIC BLOOD SAMPLE COLLECTION

Efforts will be made to obtain PK samples from all subjects on Day 1, 3 and Day 4 at sites where PK sampling is possible, as described below. Blood samples for PK analyses will be collected at the following times:

- Day 3: Pre-dose sample will be collected within 10 min before administration of the study drug
- Day 1 and Day 3: After dosing at 2-4 h; subjects who have been hospitalised are also required to have a post-dose PK sample at 6-10 h on Day 1 and Day 3
- Day 4: 24-28 h after the Day 3 dose

15.2 PHARMACOKINETIC ANALYSES

Plasma samples from nafithromycin-treated subjects will be analysed to determine nafithromycin concentrations using a validated assay. The PK population in this study will include all subjects in the safety population (from eligible sites where PK sampling is possible) who receive at least 1 dose of oral nafithromycin and have at least 1 analysable plasma PK sample.

16 MICROBIOLOGICAL ASSESSMENTS

Microbiology specimens will be collected by the sites. The details of handling and processing of microbiological specimens, all microbiological assessments, including Gram staining, organism identification, shipment of isolates and Gram stain slides to the central microbiology laboratory, etc. are provided in the Microbiology Laboratory Procedures Manual.

16.1 SCREENING RESPIRATORY SPECIMENS

An attempt should be made to collect an adequate quality expectorated or induced sputum or other deep respiratory specimen reflecting fluid from the lower respiratory tract from every subject (e.g., respiratory fluid obtained by BAL or bronchoscopy, pleural fluid obtained by thoracentesis or expectorated or induced sputum meeting adequacy criteria) at Screening. The details of handling and processing of microbiological specimens, all microbiological assessments, including Gram staining, organism identification, shipment of isolates and Gram stain slides to the central microbiology laboratory, etc. are provided in the Microbiology Laboratory Procedures Manual.

Attempts should be made to collect quality respiratory specimens before the first dose of the study drug. Respiratory specimens might be obtained as part of routine, non-study evaluation of a subject being evaluated for CABP and therefore could be obtained before obtaining informed consent. A good quality sputum specimen will be defined as meeting the following 2 criteria:

1. < 10 squamous epithelial cells (SECs) per low-power field (LPF) (magnification, x100)
2. > 25 PMNs/LPF

Processing: Sputum and tracheal samples will be rejected if they contain ≥ 10 SECs/LPF and these should NOT be processed. All sputum and tracheal samples with < 10 SECs will be processed, irrespective of the PMN count.

Resubmission: Additional sputum/tracheal aspirate samples should be resubmitted (if possible) when original sample is not of adequate quality (≥ 10 SECs/LPF).

In the event that a sputum specimen is determined to be inadequate, or cannot be obtained before the first dose of the study drug, repeated collection of a specimen should be attempted as early as possible and not later than 24 h after the first dose of the study drug. If pleural fluid is collected for culture, the pleural fluid sample should be collected in 1 aerobic blood culture bottle and 1 anaerobic blood culture bottle for a total of 2 bottles (if limited pleural fluid is available, collect the fluid in the aerobic bottle at a minimum). Pleural fluid can be collected in sterile caps for Gram staining and culture. Pleural fluid cultures must be obtained during thoracentesis or initial chest tube placement; cultures are not acceptable if obtained from an indwelling chest tube. Respiratory specimens should be processed as per details provided in the Microbiology Laboratory Procedures Manual. Culture results are to include identification of organisms to the species level. Susceptibility testing for nafithromycin will not be available to the local laboratory. Nafithromycin susceptibility testing will be performed at the central laboratory; however, these results will not be available to the Investigator during real-time management of the subjects. Therefore, decisions related to subject care (e.g. study drug discontinuation) will be based on the evaluation of the clinical signs and symptoms of CABP, rather than specific nafithromycin susceptibility data.

With the exception of respiratory 'contaminants' listed below, all isolates identified from expectorated or induced sputum specimens and/or those that are isolated from respiratory specimens or blood or pleural fluid are potential pathogens as defined in the Microbiology Laboratory Procedures Manual. The isolated pathogens will be tested by central laboratory to confirm identification and for standardised susceptibility testing of nafithromycin, moxifloxacin and additional antibiotics currently approved for CABP. For the purposes of this study, the following respiratory specimen isolates are considered 'contaminants' and should not to be sent to the central laboratory:

- Normal respiratory microflora, mixed respiratory microflora or equivalent (including, but not limited to viridans group streptococci, coagulase-negative staphylococci, *Corynebacterium* spp.)
- Fungal spp. (e.g. *Candida* spp. moulds)

For further detail, refer to the study-specific Microbiology Laboratory Procedures Manual including specific procedures pertaining to the collection, processing, storage and shipment of microbiological samples.

For the subjects being enrolled at Indian sites, an expectorated sputum sample will be collected for diagnostic evaluation of tuberculosis by Xpert TB test (using GeneXpert) in addition to collection of expectorated sputum or other deep respiratory sample for microbiological assessments. Collection of two sputum/respiratory samples, on the day of screening, could be scheduled at the discretion of investigator based on convenience of the subject.

16.2 SCREENING BLOOD CULTURES

At Screening, 1 aerobic blood culture bottle and 1 anaerobic blood culture bottle from 2 separate sites for a total of 4 bottles must be collected for culture (i.e. 1 aerobic and 1 anaerobic bottle from 2 separate venipuncture sites, for a total of 4 bottles). Either an automated or a manual system can be used for blood culture and identification methods according to the preference of the microbiological laboratory performing the testing. Refer to the Microbiology Laboratory Procedures Manual for details. Culture results are to include identification of pathogens to the species level. Susceptibility testing for nafithromycin will not be available to the local laboratory; susceptibility testing will be determined at the central laboratory. Therefore, decisions related to subject care (e.g. study drug discontinuation) will be based on the evolution of the clinical signs and symptoms of CABP.

16.3 SCREENING URINARY ANTIGEN TESTS

At the Screening visit, urine will be collected to test for *L. pneumophila* and *S. pneumoniae* antigens. The details of testing will be provided in the laboratory manual.

16.4 SEROLOGY FOR ATYPICAL BACTERIAL TITERS

At the Screening, TOC and FU visits, wherever possible, blood samples will be collected for serology testing for *L. pneumophila*, *M. pneumoniae* and *C. pneumoniae* by the central laboratory.

16.5 POST-BASELINE MICROBIOLOGICAL ASSESSMENTS

At any time after the Screening visit, respiratory specimen and blood cultures should be obtained as clinically indicated. As CABP responds to therapy, obtaining repeated specimens for culture or examination may not be clinically appropriate and/or there may be no material for culture. If study drug is prematurely discontinued because of insufficient effect of the study drug (e.g. failure at EOT or TOC or clinical relapse at any time), an appropriate

respiratory specimen and blood should be obtained for culture on the day of discontinuation; if new antibacterial therapy is administered to treat the current CABP after premature discontinuation of the study drug, it is preferred that respiratory specimens and blood be collected for culture after stopping the study drug but before new treatment is started. Blood cultures should be repeated upon knowledge of a positive result from any previous collection until sterilisation is confirmed (Table 1). Repeat, post-baseline urinary antigen tests for *L. pneumophila* or *S. pneumoniae* should not be performed.

16.6 CENTRAL MICROBIOLOGY LABORATORY PROCEDURES

With the exception of 'contaminants' defined above (Section 16.1), all cultured bacterial isolates collected at each visit will be shipped to the designated central laboratory for each study site. The central microbiology laboratory will perform the following procedures:

1. Confirm the identity of all bacterial isolates to the species level
2. Perform anti-microbial susceptibility testing of all bacterial isolates for susceptibility to nafithromycin, moxifloxacin and other antimicrobial agents using standardised methods
3. At the Screening, TOC and FU visits, wherever possible, blood samples should be tested for atypical acute and convalescent serology, for *L. pneumophila*, *M. pneumoniae* and *C. pneumoniae* (Section 16.1)

Any questions regarding microbiological procedures, interpretation of results or storage of isolates should be discussed with the Sponsor or designee. Additional details are available in the Microbiology Laboratory Procedures Manual.

17 EFFICACY EVALUATION

17.1 PRIMARY AND SECONDARY EFFICACY VARIABLES

17.1.1 Primary Efficacy Variable

- Clinical Response at Day 4 (MITT analysis set)

17.1.2 Secondary Efficacy Variables

- Clinical response at Day 4 (mMITT and CE analyses sets)
- Clinical outcome at EOT (MITT, mMITT and CE analyses sets)
- Clinical outcome at TOC (MITT, mMITT and CE analyses sets)
- Hospitalisation prior to FU (MITT analysis set)
- By-pathogen clinical response at Day 4 and by-pathogen clinical outcome at TOC (mMITT analyses sets)

17.2 CLINICAL OUTCOME ASSESSMENTS

17.2.1 Clinical Response on Day 4

Clinical response (Favourable, Non-favourable or Indeterminate) on Day 4 will be evaluated in the MITT analysis set as the primary outcome as well as in the mMITT and CE analyses sets as a secondary efficacy evaluation (Table 2). Clinical response is determined programmatically using the Investigator's determination of CABP symptoms entered into the eCRF. The Investigator is not responsible for categorising subjects as having clinical response, non-response or indeterminate on Day 4. The severity of the subject CABP symptoms of dyspnoea (shortness of breath), cough, production of purulent sputum or pleuritic chest pain will be evaluated on a 4-point scale (absent, mild, moderate or severe) based upon the CABP Symptom Severity Guidance in Appendix III, Section 23.3.

Table 2. Clinical Response Assessment on Day 4.

Outcome	Definition
**Favourable Clinical Response**	Alive and improvement of at least 1 level (e.g. severe to moderate, moderate to mild, mild to absent) in at least 2 CABP symptoms (dyspnoea, cough, production of purulent sputum or pleuritic chest pain) compared with that at the Baseline Visit, without worsening in any other of the 4 CABP symptoms. Severity of symptoms is based on a 4-point scale (absent, mild, moderate or severe).
**Unfavourable Clinical Response**	No improvement of at least 1 level in at least 2 CABP symptoms compared with that at the Baseline Visit; or worsening in any of the 4 CABP symptoms compared with that at the Baseline Visit; or death from any cause at or before Day 4.
**Indeterminate**	Study data are missing for the evaluation of efficacy at Day 4 for any reason, including loss to follow-up.

Abbreviation: CABP = community-acquired bacterial pneumonia

17.2.2 Clinical Outcome at EOT and TOC

Clinical outcome (Cure, Failure or Indeterminate) at EOT and TOC in the MITT, mMITT and CE analyses sets is the secondary efficacy endpoint in this study. The assessment of signs and symptoms will be done by the

Investigator using the CABP Symptom Severity Guidance in Appendix III, Section 23.3 at EOT (+ 2 days) and Day 15 (± 4 days). The definition of the clinical outcome at EOT and TOC is provided in Table 3. The Investigator will categorise the subject's outcome as Cure, Failure or Indeterminate. Clinical Failure at EOT will be carried forward to the TOC Visit.

Table 3. Clinical Outcome Assessments at EOT and TOC.

Outcome	Definition
**Clinical Cure**	Alive and signs and symptoms of CABP (dyspnoea, production of purulent sputum or pleuritic chest pain) are resolved or return to premorbid conditions and cough is improved such that further antibacterial therapy is not needed and the subject otherwise cannot be declared a clinical failure
**Clinical Failure**	The signs and symptoms of CABP (dyspnoea, production of purulent sputum or pleuritic chest pain) did not resolve or return to pre-morbid and/or cough worsened, such that non-study antibacterial therapy must be initiated for the treatment of CABP or death occurred prior to assessment or subject received alternative anti-microbial therapy for the treatment of CABP prior to this visit
**Indeterminate**	Study data are missing for the evaluation of efficacy at the assessment visit (EOT/TOC) for any reason, including loss to follow-up

Abbreviations: CABP = community-acquired bacterial pneumonia, EOT = end-of-treatment, TOC = Test-of-Cure

17.2.3 Hospitalisation Before the Follow-Up Visit

An assessment of hospitalisation (defined as re-admission to the hospital if previously hospitalised on or after study drug initiation [Day 1] or initial hospital admission [if not previously hospitalised]) for any reason between the initiation of the study drug (Day 1) and FU will be made based on information collected in the eCRF for subjects in MITT analyses set. For the purposes of this secondary efficacy assessment, 'hospital admission' is defined as inpatient admission for at least 24 h in an acute care facility (including inpatient hospital ward or emergency room); by contrast, admission to nursing homes, assisted living facilities, rehabilitation units or acute care facilities (hospital wards or emergency rooms) for less than 24 h is not considered a formal hospital admission. The assessment of hospitalisation within 30 days of study drug initiation is defined in Table 4.

Hospitalisation for convenience or social purposes, as mentioned in section 11.1, will not qualify for the secondary efficacy assessment.

Table 4. Hospitalisation Before the Follow-Up Visit.

Outcome	Definition
**Hospitalisation Before FU**	Meets **any** of the following criteria:<br>Hospital re-admission for any reason between the Day 1 and the FU visits, if previously hospitalised and discharged<br>Initial hospital admission for any reason between the Day 2 and the FU visits, if not previously hospitalised on Day 1
**No Hospitalisation Before FU**	Does not meet the criteria for hospitalisation within 30 days of study drug initiation as defined above or till FU whichever is earlier
**Indeterminate**	Study data are missing for the evaluation of efficacy at the FU Visit for any reason, including loss to follow-up

Abbreviation: FU = follow-up

17.3 BY-PATHOGEN CLINICAL RESPONSE AT DAY 4

For subjects in the mMITT analysis set, the by-pathogen clinical response at Day 4 will be the same as the corresponding subject's response at Day 4, i.e. Favourable, Unfavourable or Indeterminate

17.4 BY-PATHOGEN CLINICAL OUTCOME AT TOC

For subjects in the mMITT analysis set, the by-pathogen clinical outcome at TOC will be the same as the corresponding subject's outcome at TOC, i.e. Cure, Failure or Indeterminate.

17.5 EMERGENT INFECTIONS

CABP caused by pathogens first appearing after Screening (emergent infections) will be categorised as either super infections or new infections as defined in Table 5.

Table 5. Emergent Infections.

Category	Definition
**Super infection**	Isolation of a new pathogen(s) (other than the original CABP pathogen[s]) from an appropriate post-baseline respiratory specimen (e.g. respiratory fluid obtained by bronchoalveolar lavage or bronchoscopy; pleural fluid obtained by thoracentesis; or expectorated or induced sputum meeting adequacy criteria) culture or blood culture, which is accompanied by signs and symptoms of infection requiring alternative systemic anti-microbial therapy during the period *up to and including* EOT
**New infection**	Isolation of a new pathogen(s) (other than the original CABP pathogen[s]) from an appropriate post-baseline respiratory specimen culture or blood culture, which is accompanied by signs and symptoms of infection requiring alternative systemic anti-microbial therapy in the time period *after* EOT (e.g. TOC)

Abbreviations: CABP = community-acquired bacterial pneumonia; EOT = End of Treatment; TOC = Test of Cure

18 SAFETY EVALUATION

18.1 SPECIFICATION OF SAFETY PARAMETERS

Subjects must be evaluated by a physician or an appropriately trained healthcare professional at every study visit and the evaluation must be documented. The procedures discussed below will be completed at the designated visits as outlined in Section 14.

18.1.1 Definition of Adverse Event

An AE is any untoward medical occurrence in a patient or clinical trial subject administered a pharmaceutical product and which does not necessarily have to have a causal relationship with this treatment. An AE can, therefore, be any unfavourable and unintended sign (including an abnormal laboratory finding, for example), symptom or disease temporally associated with the use of a medicinal product, whether or not considered related to the medicinal product (International Council for Harmonisation [ICH]-E2A guideline).

AEs may also include post-treatment complications that occur as a result of protocol-mandated procedures (e.g. invasive procedures such as venipuncture and biopsy). Pre-existing events that increase in severity or change in nature during or, as a consequence of, use of a medicinal product in a human clinical study will also be considered AEs.

Any pre-existing medical condition or diagnosis associated with a clinically significant laboratory abnormality should be documented in the case report form.

An AE does not include the following

- Medical or surgical procedures (e.g. surgery, endoscopy, tooth extraction, transfusion); the condition that necessitates the procedure is an AE. Any pre-existing medical condition that necessitates a procedure during the study should be evaluated for AE reporting
- Any pre-existing disease or condition or laboratory abnormality present or detected before the start of the study treatment regimen that does not worsen
- Laboratory abnormalities without clinical manifestations, which do not require medical intervention, or that do not result in termination or delay of study drug administration
- Situations where an untoward medical occurrence has not occurred (e.g. hospitalisation for elective surgery, social and/or convenience admissions)
- Overdose of any study treatment or concomitant medication without any signs or symptoms, unless the subject is hospitalised for observation
- Progression of the index CABP or insufficient therapeutic effect of the study drug, which is captured as an efficacy outcome (i.e. clinical failure at EOT or TOC)
- Progression of disease or insufficient therapeutic effect which causes new hospitalisation (in subjects not previously hospitalised for the index CABP), rehospitalisation (in subjects who were previously

hospitalised for the index CABP and discharged); however, if this leads to death, it should be recorded as an SAE (Section 18.4.2)

A TEAE is defined as an AE or SAE that occurs during or after the first administration of the study drug and up through the FU Visit or a pre-existing AE that worsens in severity after drug administration.

A life-threatening AE is an AE that in the view of either the Investigator or Sponsor places the subject at immediate risk of death. It does not include an AE that had it occurred in a more severe form might have caused death.

18.1.2 Definition of Serious Adverse Event

An SAE is any adverse experience that occurs from the signing of the informed consent to the FU Visit and that results in any of the following outcomes:

- Death
- Life-threatening situation (subject is at immediate risk of death)
- Inpatient hospitalisation or prolongation of existing hospitalisation
- Persistent or significant disability/incapacity
- Congenital anomaly/birth defect in the offspring of a subject who received study treatment
- Events that jeopardise the subject sufficiently that medical or surgical intervention may be required to prevent one of the above outcomes

Progression of disease or insufficient therapeutic effect which causes new hospitalisation (in subjects not previously hospitalised for the index CABP) or re-hospitalisation (in subjects that were previously hospitalised for the index CABP and discharged) is not a SAE; however, if this leads to death, it should be recorded as an SAE. Hospitalisation for convenience or social purposes, as mentioned in section 11.1, should not be recorded as an SAE.

18.1.3 Definition of Unexpected Adverse Event

An AE is considered 'unexpected' if it is not listed in the IB or is not listed at the specificity or severity that has been observed. For example, under this definition, hepatic necrosis would be unexpected (by virtue of greater severity) if the IB referred only to elevated hepatic enzymes or hepatitis. Similarly, cerebral thromboembolism and cerebral vasculitis would be unexpected (by virtue of greater specificity) if the IB listed only cerebral vascular accidents. 'Unexpected', as used in this definition, also refers to AEs, that are mentioned in the IB as occurring with a class of drugs or as anticipated from the pharmacological properties of the drug, but are not specifically mentioned as occurring with the particular drug under investigation.

Progressive worsening of the index CABP or insufficient therapeutic effect of the study drug that leads to hospitalisation or death (i.e. SAE criteria, Section 18.4.2) is considered expected as part of potential disease progression and not unexpected.

Some AEs are listed in the IB as occurring with the same class of drugs or as anticipated from the pharmacological properties of the drug even though they have not been observed with the drug under investigation. Such events would be considered unexpected until they have been observed with the drug under investigation.

The list of AEs included in the current version of the IB will be used as reference safety information.

18.2 CLASSIFICATION OF AN ADVERSE EVENT

18.2.1 Severity of Event

The Investigator will be asked to provide an assessment of the severity of the AE using the following categories: mild, moderate or severe. This assessment is subjective and the Investigator should use medical judgement to compare the reported AE to similar types of events observed in clinical practice. *Severity*, which is a description of the intensity of manifestation of the AE, is distinct from *seriousness*, for which specific SAE criteria are met (Section 18.4.2).

- **Mild:** Symptom(s) barely noticeable to the subject or does not make the subject uncomfortable. The AE does not influence performance or functioning. Prescription drugs are not ordinarily needed for relief of symptom(s).
- **Moderate:** Symptom(s) of a sufficient severity to make the subject uncomfortable. Performance of daily activities is influenced. Treatment of symptom(s) may be needed.
- **Severe:** Symptom(s) of a sufficient severity to cause the subject severe discomfort. Severity may cause cessation of treatment with the drug. Treatment for symptom(s) needed.

18.2.2 Relationship to Study Agent

For each reported AE, the Investigator must make an assessment of the relationship of the event to the study drug using the following scale:

- **Unrelated:** The event is definitely not associated with administration of the study treatment, and is judged clearly due to causes other than the study treatment. Clinical failure of CABP due to insufficient therapeutic effect of the study drug is also considered 'unrelated' to the study drug.
- **Related:** The event is possibly or probably associated with administration of study treatment. Possibly related events follow a reasonable temporal sequence from administration of study treatment, but may be due to another cause and could also be reasonably explained by the subject's clinical state or other modes of therapy administered to the subject. Probably related events follow a reasonable temporal sequence from administration of the study treatment, but are not easily explained by another cause such as known characteristics of the subject's clinical state or other treatment, and are confirmed by improvement after stopping the study treatment.

These criteria, in addition to good clinical judgement, should be used as a guide for determining the causal assessment. If the event is believed to be unrelated to the study treatment, then an alternative explanation should be provided.

18.3 TIME PERIOD AND FREQUENCY FOR EVENT ASSESSMENT AND FOLLOW-UP

The occurrence of an AE or SAE may come to the attention of study personnel during study visits and interviews of a study participant presenting for medical care or upon review by a study monitor. All AEs including local and systemic reactions not meeting the criteria for SAEs will be captured on the appropriate eCRF. Information to be

collected includes event description, time of onset, clinician's assessment of severity, relationship to study product (assessed only by those with the training and authority to make a diagnosis) and time of resolution/stabilisation of the event. All AEs occurring while on study must be documented appropriately regardless of relationship. All AEs will be followed to adequate resolution.

Any medical condition that is present at the time that the participant is screened will be considered as baseline and not reported as an AE. However, if the study participant's condition deteriorates at any time during the study, it will be recorded as an AE.

Changes in the severity of an AE will be documented to allow an assessment of the duration of the event at each level of severity to be performed. AEs characterised as intermittent require documentation of onset and duration of each episode.

The Investigator/designee will record all reportable events with start dates occurring any time after informed consent is obtained until follow- up visit. Deaths occurring within 40 days of first dose should be reported, if Investigator becomes aware of the same. At each study visit, the Investigator will inquire about the occurrence of AE/SAEs since the last visit. Events will be followed for outcome information until resolution or stabilisation.

18.4 REPORTING PROCEDURES

18.4.1 Adverse Event Reporting

All AEs and SAEs will be recorded and reported from the signing of the informed consent form (ICF) to the time of the FU Visit. The Investigator must instruct the subject to report AEs during this time period. Reports of death occurring within 40 days of first dose will be reported to the Sponsor and additional information relative to the cause of death will be sought and documented.

All AEs and SAEs must be recorded on source documents. All AEs and SAEs for subjects who receive a treatment assignment will be recorded in the eCRF. The Investigator must follow up as medically necessary on all AEs and SAEs until the events have subsided, the condition has returned to baseline or in case of permanent impairment until the condition stabilises.

AEs should be based on the signs or symptoms detected during the physical examination and on clinical evaluation of the subject. In addition to the information obtained from those sources, the subject should be asked the following nonspecific question: 'How have you been feeling since your last visit'? Signs and symptoms should be recorded using standard medical terminology.

Any unanticipated risks to the subjects must be reported promptly to the relevant ethics committee(s) and regulatory agency(ies).

18.4.2 Serious Adverse Event Reporting

18.4.2.1 Collection and Reporting of SAEs

In case of an SAE, the Investigator (or designee) should immediately inform the Sponsor/designated CRO via a telephonic call, e-mail within 24 hours of occurrence of the event. Any delay to the specified timeline should be well explained and documented appropriately.

Initial SAE Form must be duly completed, signed and dated by the Investigator/ designee and sent to PV team of the Global Clinical Development Department, Wockhardt Ltd or designated CRO within 24 hours of occurrence of the event via email or fax. Relevant contact information will be provided to the clinical study sites.

Wockhardt undertakes to notify the Investigators of all SAEs that occur during the course of the study in any other location as per applicable regulations. Such events will also be reported in an expedited manner to Drug Controller General of India (DCGI) and other Regulatory Agencies, as per the applicable requirements. The Investigator will inform the local ethics committee of all SAEs and findings that could adversely affect the subjects' safety, could have an impact on the conduct of the study or could alter the IEC/IRB approval to continue the study as per the applicable country regulations and ethics committee requirements. The applicable sponsor SOP will be followed for receipt, processing and reporting of SAEs.

18.4.2.2 Follow-up of SAEs

If completed information is not available at the time of the initial report and becomes available at a later date, the Investigator should send a follow-up SAE form within 24 hours of receipt of additional information to the Sponsor.

All SAEs should be followed up until resolution of the event or until the Investigator and Sponsor judge that further follow-up is not necessary.

18.4.2.3 Regulatory Reporting Timeline

All SAEs and suspected unexpected serious adverse reactions (SUSARs) shall be reported to the respective regulatory authority as per the country-specific applicable regulatory requirements/guidelines.

18.4.3 Reporting of Pregnancies Occurring During the Study

The Investigator or designee must report every pregnancy from the time the subject signs the ICF through 30 days from last dose of study drug. Within 24 h of learning of the pregnancy, the Investigator or designee must report the event on the "Exposure in Pregnancy Form" and fax or e-mail it to the fax number or e-mail provided to the site, even if no AE has occurred. Pregnancies in female partners of male subjects occurring during the time frame described above must also be reported.

The pregnancy must be followed to term and the outcome reported by completing the Exposure in Pregnancy Form. If the pregnancy is associated with an SAE (e.g. if the gravida is hospitalised for haemorrhage), a separate SAE Form must be filed as described in Section 18.4.2 with the appropriate seriousness criterion (e.g. hospitalisation) indicated, in addition to the Exposure in Pregnancy Form.

An exposure during pregnancy (also referred to as exposure in-utero) occurs if:

- A female subject becomes or is found to be pregnant either while receiving the study drug during her participation in the clinical study, or the female subject becomes pregnant within 30 days of last dose of the study drug (maternal exposure)
- A male subject has been exposed to the study drug during his participation in the clinical study around the time of conception of the partner's pregnancy (paternal exposure)

During the conduct of the trial, if pregnancy is reported in a female subject, the Investigator should withdraw the subject immediately from the trial and inform the PV team within 24 h of knowledge of the event. The Investigator should complete and send the signed Exposure in Pregnancy Form to the PV team. Any other maternal SAE in the course of pregnancy will be reported on a separate SAE form. If the outcome of the pregnancy meets the criteria for an SAE (i.e. ectopic pregnancy, spontaneous abortion, intrauterine foetal demise, neonatal death or congenital anomaly [in a live born, a terminated foetus, an intrauterine foetal demise or a neonatal death]), the Investigator should follow the procedures for reporting SAEs.

Additional information about pregnancy outcomes that are reported as SAEs are as follows:

- Spontaneous abortion includes miscarriage and missed abortion; neonatal deaths that occur within 1 month of birth should be reported, without regard to causality, as SAEs. In addition, infant death after 1 month should be reported as an SAE when the Investigator assesses the neonatal death as related or possibly related to exposure to investigational product.
- In the case of paternal exposure, the study subject should be provided with the Pregnant Partner Release of Information Form to deliver to his partner. The Investigator must document on the Exposure in Pregnancy form that the subject was given this letter to provide to his partner. If the pregnancy outcome fulfils the criteria for an SAE, it will be reported to the respective Regulatory Authorities in expedited manner.
- A pregnant female will be followed up within 15 calendar days after Expected Date of Delivery for the pregnancy outcome. A second follow-up will be done after 30 calendar days of pregnancy outcome.

Additional information regarding the exposure during pregnancy may be requested by the Investigator. Further follow-up of birth outcomes will be handled on a case-by-case basis (e.g. follow-up on preterm infants to identify developmental delays).

18.5 SAFETY OVERSIGHT

An internal Data Monitoring Committee (DMC) will be utilised in this study. The DMC will perform periodic reviews of data from the study to evaluate subject safety, compliance with the study protocol and the quality of data. Safety of study drug will be monitored by reviewing AEs, SAEs and the safety laboratory assessments on a regular basis. If specific issues are identified that warrant more extensive review, the DMC will recommend that experts external to the company review the data and provide a formal opinion of their significance and recommend any necessary interventions.

19 STATISTICAL METHODS

19.1 ANALYSES SETS

19.1.1 Intent-to-Treat Analysis Set

The ITT analysis set will include all subjects who were randomised, regardless of whether the subject actually received the study drug.

19.1.2 Modified Intent to Treat (MITT)

All ITT subjects who are randomized and received at least one dose of study drug. Subjects with detection of MTBC (as per results of Xpert TB test) indicative of active pulmonary tuberculosis will be excluded from this analysis set.

19.1.3 Safety Analysis Set

The safety analysis set will include all randomised subjects who receive any amount of the study drug. Subjects will be analysed according to the treatment actually received.

19.1.4 Microbiological Modified Intent-to-Treat Analysis Set

All MITT subjects who have received at least 1 dose of study drug and have at least 1 baseline pathogen known to cause CABP against which the investigational drug has antibacterial activity, including bacterial pathogens identified by respiratory specimen culture, blood culture, and/or urinary antigen test (*S. pneumoniae*, *S. aureus, H. influenzae, H. parainfluenzae, M. catarrhalis, L. pneumophila*) or atypical bacterial serologic response (*M. pneumoniae*, *C. pneumoniae*, *L. pneumophila*) will be included in this analysis set. Subjects with sole baseline Gram-negative bacterial infection with bacteria from the *Enterobacteriaceae*, *Pseudomonadaceae* or *Yersiniaceae* will be excluded from this analysis set.

19.1.5 Clinically Evaluable Analysis Set

The CE analysis set will include all MITT subjects who follow important components of the trial. To be included in the CE analysis set, subjects must meet all of the following criteria:

- Meet key Inclusion Criteria, including the clinical disease criteria for CABP (Inclusion Criteria #3a, #3b, #3c, #3d and #3e)
- Do not meet key Exclusion Criteria (#1 through # 6, #16 and #18)
- The TOC Visit occurred within a window of 15 ± 4 days from the date of randomisation unless the subject was deemed a clinical failure before this visit
- Do not receive non-study, potentially effective against the baseline pathogen(s), systemic antibacterial therapy between Day 1 and the assessment TOC
- Do not have a clinical outcome of Indeterminate at the TOC Visit
- Receive at least 80% of the intended doses of randomised study drug therapy (based on number of tablets dispensed/received)

- Receive at least 48 h of study drug therapy to be considered an evaluable clinical failure and at least 72 h of study drug therapy to be considered an evaluable clinical success
- Do not have any other major protocol violations that may confound efficacy assessments at TOC

19.1.6 Pharmacokinetic Analysis Set

The PK analysis set includes all subjects in the safety analysis set who received at least 1 dose of nafithromycin and had at least 1 analysable plasma PK sample.

19.1.7 Determination of Sample Size

This study is designed to demonstrate non-inferiority of oral nafithromycin (800 mg once daily) compared with oral moxifloxacin (400 mg once daily).

Nafithromycin will be declared non-inferior to moxifloxacin if the lower limit of the 2-sided 95% confidence interval (CI) for the difference between treatment groups (nafithromycin minus moxifloxacin) in the proportion of subjects with a favourable clinical response at Day 4 in the MITT analysis set is greater than -0.125.

In these comparisons, the margin of 12.5% has been determined based on historic data regarding the treatment effect of antibiotics.

For the determination of the study sample size, favourable clinical response rates at Day 4, obtained from studies of subjects with CABP, of 85% to 90% are projected, with an anticipated dropout rate by Day 4 of at most 5%. However, since subjects who dropout from the study prior to Day 4 will be included in the denominator for the calculation of the proportion of subjects with favourable clinical response at Day 4, an attained favourable response rate at Day 4 of 81% to 86% is anticipated in each treatment group. Based on these attained favourable clinical response rates at Day 4, a non-inferiority margin of 12.5%, and using the Farrington-Manning sample size approach for the Miettinen and Nurminen method, it has been determined that approximately 414 adult subjects (207/arm) will need to be included in the MITT analyses set in order for the statistical test to have a-priori at least 90% power at the 1-sided 2.5% significance level when the attained favourable response rate is at least 81%. Given the number of subjects needed in the MITT (N=414) analyses set and assuming a TB rate of 15%, 488 subjects will need to be enrolled into the study.

19.1.7.1 Methods of Analysis

All data will be summarised separately by each treatment group (i.e., nafithromycin or moxifloxacin). Descriptive statistics (mean, standard deviation, median, minimum and maximum) will be presented for continuous variables for each study drug. Frequency distributions (counts and percentages) will be presented for categorical variables.

19.1.8 Analysis of Disposition and Subject Characteristics

Subject disposition (enrolment, premature discontinuations from study medication and from the study) and major protocol deviations will be summarised by treatment group in the ITT and MITT analyses sets. Reasons for exclusions from analyses sets will be tabulated. Demographics and baseline characteristics such as PORT scores, age, gender, race, weight, relevant medical history, geographic region and clinical signs and symptoms will be

summarised by treatment group in the MITT analyses set. Tallies of baseline pathogens obtained from respiratory cultures or blood, serology and urinary antigen samples will be generated for the subjects in the mMITT analysis set.

19.1.9 Efficacy Analyses

For all efficacy analyses, subject data will be analysed in the group to which the subject was randomised.

19.1.9.1 Primary Efficacy Analyses

The primary efficacy endpoint for this study is the proportion of subjects with favourable clinical response at Day 4 in the MITT analyses set. Subjects will be categorised as having Favourable, Unfavourable or Indeterminate response. Subjects with missing data or who are lost to FU are defined as Indeterminate and are included in the denominator for the calculation of the proportion of subjects with Favourable clinical response at Day 4 in the MITT analyses set. The number and percentage of subjects in each treatment group and in each response category will be reported.

The null (H_0) and alternative (H_1) hypotheses are the following:

$$H_0: \pi_1 - \pi_2 \leq -0.125 \text{ vs. } H_1: \pi_1 - \pi_2 > -0.125$$

Where:

π_1 = the proportion of subjects with favourable clinical response at Day 4 in the nafithromycin treatment group

π_2 = the proportion of subjects with favourable clinical response at Day 4 in the moxifloxacin treatment group

The non-inferiority hypothesis test is a 1-sided hypothesis test performed at the 2.5% level of significance. The statistical test is based on the lower limit of the 2-sided 95% CIs for the observed difference in the proportion of subjects with favourable clinical response at Day 4 (nafithromycin minus moxifloxacin). The CI is obtained using the method of Miettinen and Nurminen.

If the lower limit of the 2-sided 95% CI for the difference between treatment groups (nafithromycin minus moxifloxacin) in the proportion of subjects with favourable clinical response rates at Day 4 in the MITT analyses set is greater than –0.125, the null hypothesis H_0 will be rejected in favour of the alternative hypothesis H_1 and the non-inferiority of nafithromycin monotherapy to moxifloxacin will be concluded. In addition, if the lower limit of the 95.0% CI is greater than zero, the superiority of nafithromycin monotherapy to moxifloxacin monotherapy will also be concluded.

19.1.9.1.1.1 Additional Analyses of the Primary Efficacy Outcomes

For the primary analysis variable, a sensitivity analysis will be conducted using the Miettinen-Nurminen statistic stratified by the randomisation factor: PORT score. Other sensitivity analyses of the primary efficacy outcome described in the Statistical Analysis Plan will be conducted. In addition, the clinical response at Day 4 will be compared between treatment groups in the mMITT and CE analyses sets.

19.1.9.2 Secondary Efficacy Analyses

The secondary analysis variables are listed in Section 17.1.2. For each by-subject secondary efficacy outcome, the number and percentage of subjects with each response category (e.g., for the clinical outcome at EOT response is either Cure, Failure or Indeterminate) will be summarised by treatment group. The 2-sided 95.0% CI for the difference between treatment groups in the proportion of subjects with favourable response (e.g., for the clinical outcome at EOT: the difference between treatment groups in proportions of subjects with clinical cure at EOT) will be presented using the same approach as for the primary analyses. These summaries will be done for the outcomes/visits/analyses set combinations shown below (Table 6).

Table 6. Secondary By-Subject Analysis Variables by Visit and Analyses Set.

Analysis Variable	Visit	Analysis Set		
		**MITT**	**mMITT**	**CE (at TOC)**
**Clinical Response**	Day 4	NA (primary)	X	X
**Clinical Outcome**	EOT	X	X	X
	TOC	X	X	X
**Hospital re-admission rates**	FU	X		

Abbreviations: CE = Clinically Evaluable; M ITT = Modified Intent-to-Treat; mMITT = Microbiological Modified Intent-to-Treat; TOC = Test of Cure

Similar tabulations will be done for the by-subject clinical response at Day 4 and the by-subject clinical outcome at TOC in subjects with bacteraemia and in subjects with at least one MDR pathogen (identified from an appropriately collected respiratory specimen or blood) in the mMITT analysis set, except that CIs will not be included if less than 20 subjects per treatment group are obtained.

The by-pathogen clinical response at Day 4 and also the by-pathogen clinical outcome at TOC will be summarised in the mMITT analyses set. For each by-pathogen outcome, the number and percentage of subjects with each response category (e.g., for the TOC Visit: Cure, Failure or Indeterminate) will be tabulated by treatment group and baseline pathogen (obtained from respiratory or blood cultures, antigen or serology tests).

The incidence of subjects with super infections and new infections will be tabulated and listings by pathogen will be provided for each treatment group. In addition, for subjects in the mMITT analyses set with a pathogen identified from a respiratory culture or blood, a listing of pathogens with decreased susceptibility (defined as a 4-fold increase in the baseline MIC) to the study treatment will be provided.

Subgroup analyses of the primary efficacy variable in the MITT analyses set will be presented. These include summaries by key demographic and baseline factors as describe in the SAP.

Other analyses as described in the Statistical Analysis Plan will also be conducted.

19.1.10 Safety Analyses

Safety will be analysed in the safety analysis set (Section 19.1.3) according to the treatment actually received.

All AEs will be tabulated by system organ class (SOC), PT, corresponding severity and relationship to the study drug (per Common Terminology Criteria for Adverse Events (version 4.03, 2010).

Safety will be evaluated by presenting summaries of AEs, vital signs, laboratory evaluations (haematology , chemistry panel, urine analysis) and ECG parameters. For each safety parameter, unless otherwise stated, the last assessment made before the first administration of the study drug will be used as the baseline value for all analyses.

The incidence of subjects with TEAEs (defined in Section 18.4.1) will be presented by SOC and PT according to the Medical Dictionary for Regulatory Activities®, by relationship to the administration of the study drug and by severity. In addition, the incidence of subjects with SAEs and TEAEs leading to discontinuation of the study drug will be presented by SOC, PT and relationship to the study drug. If the incidence of SAEs and AEs leading to discontinuation of the study drug is low, only a listing will be provided.

Descriptive statistics, including mean and mean changes from baseline, for clinical laboratory tests (haematology and chemistry) will be presented by study visit. The number and percentage of subjects with PCS changes in clinical laboratory results will be determined based on the combination of value outside normal limits and magnitude of changes/percent changes from baseline and will be summarised for the worst post-baseline values.

The number and percentage of subjects with a PCS change in vital signs will be presented for the minimum and maximum post-baseline values. Descriptive statistics, including mean and mean changes from baseline, for ECG parameters will be presented for each time point measured. The number and percentage of subjects with a PCS change will be tabulated for the minimum and maximum post-baseline values. PCS changes in ECG parameters will be tabulated. PCS changes in laboratory parameters, vital signs and ECG values will be defined in the Statistical Analysis Plan.

All safety data, including physical examination and urinalysis results, will be provided in by-subject listings.

19.1.11 Pharmacokinetic Analyses

The PK analyses set in this study will include all subjects in the safety population (from eligible sites where PK sampling is possible) who receive at least 1 dose of oral nafithromycin and have at least 1 analysable plasma PK sample. Descriptive statistics for the blood concentrations at each timepoint and PK parameters (Cmax and AUC) will be summarised in the PK analysis set. Plasma concentrations of nafithromycin will be listed for each subject in the PK analysis set.

19.1.12 Interim Analysis

No formal interim analysis of efficacy is planned. A blinded (aggregated across treatment groups) review of the percentage of subjects in the ITT population who tested positive for MTBC (as per the results of the Xpert TB test) will be conducted when approximately 50% of subjects have been enrolled. If the percentage subjects who tested positive for MTBC is higher than anticipated, the target number of enrolled subjects may be increased to ensure the study is sufficiently powered for the MITT analyses set.

19.2 HANDLING OF DROPOUTS AND MISSING DATA

Every effort will be made to collect all data at specified times.

Missing data will be handled as follows:

- All missing and partial dates for events occurring after randomisation or for medications received after randomisation will be queried for a value. If no value can be obtained, imputations as detailed in the Statistical Analysis Plan will be applied.
- Missing times, severity and causality for AEs will be queried for a value. No imputations will be made for missing times. AEs with a missing time will be considered treatment emergent if the date is on or after the first dose of the study drug, AEs with missing severity will be considered severe and AEs with a missing relationship to the study drug will be considered related to the study drug.
- For the primary efficacy outcome at Day 4, subjects with missing data will be considered Indeterminate response. By definition, subjects with Indeterminate response are included in the denominator for analyses in the MITT analyses set. Sensitivity analyses as described in the Statistical Analysis Plan will assess the effect of missing data on the analysis of the primary efficacy outcome. Missing values for other efficacy outcomes will be handled in a similar manner as the primary efficacy outcome.
- For evaluations at the TOC Visit, subjects with Indeterminate response are included in the denominator for analyses in the MITT analyses set and are excluded from the CE analysis set at TOC.
- Individual data points will remain as missing, and missing data will not be imputed except as detailed in the Statistical Analysis Plan.

19.3 MEASURES TO MINIMISE BIAS

This is a double-blind, multicentre, randomised study. The following measures will be taken to minimise bias during randomisation.

19.3.1 Enrolment/Randomisation/Masking Procedures

All the subjects signing the informed consent and meeting the eligibility criteria will be enrolled in the study. Subjects will be randomised using block randomisation method to avoid bias while assigning the treatment arm. Subjects will be randomly assigned in blinded manner to 1 of the 2 treatment arms using an IXRS in the ratio of 1:1. Enrolment of PORT Risk Class II will be capped at 60% and enrolment of subjects with allowed prior systemic antibiotic use will be capped initially at 25% (subject to change during study conduct).

20 ETHICS/PROTECTION OF HUMAN SUBJECTS

20.1 ETHICAL STANDARD

The Investigator will ensure that this study is conducted in full conformity with Regulations for the Protection of Human Subjects of Research codified in 45 Code of Federal Regulations (CFR) Part 46, 21 CFR Part 50, 21 CFR Part 56, the ICH E6, Declaration of Helsinki and Schedule Y (India).

20.2 INSTITUTIONAL REVIEW BOARD

The protocol and protocol amendments, ICF(s), case report forms, recruitment materials and all participant materials will be submitted to the IRB/IEC for review and approval. Approval of the protocol and informed consent forms will be obtained before any participant is enrolled. Any amendment to the protocol will require review and approval by the IRB/IEC before the changes are implemented to the study. All changes to the consent form will be approved by the IRB/IEC; a determination will be made regarding whether previously consented participants need to be re-consented.

20.3 INFORMED CONSENT PROCESS

20.3.1 Consent and Other Informational Documents Provided to Participants

Consent forms describing in detail the study agent, study procedures and risks are given to the participant and written documentation of informed consent is required before starting intervention/administering the study product. The ICFs will be translated into local languages, based on the templates provided by the Sponsor. The accuracy of the translations will be ensured by performing an independent back-translation. The translated versions will also be submitted for review and approval by study site IRBs/IECs.

20.3.2 Consent Procedures and Documentation

This study will be conducted in compliance with the current ICH E6 Good Clinical Practice (GCP) guideline pertaining to informed consent, the current US CFR (Title 21, Parts 50 Subparts B and 56) as well as relevant Indian guidelines (Schedule Y). Subjects will give written consent to participate in the study at the first visit, before initiation of any study-related procedures, after having been informed about the nature and purpose of the study, participation and termination conditions, risks and benefits. Informed consent is a process that is initiated before the individual's agreeing to participate in the study and continues throughout the individual's study participation. Extensive discussion of risks and possible benefits of participation will be provided to the participants. Consent forms will be approved by the IRB/IEC. The participant will be asked to read and review the document. The Investigator will explain the research study to the participant and answer any questions that may arise. All participants will receive a verbal explanation in terms suited to their comprehension of the purposes, procedures and potential risks of the study and of their rights as research participants. Participants will have the opportunity to carefully review the written consent form and ask questions before signing. The participants should have the opportunity to discuss the study with their surrogates or think about it before agreeing to participate. The participant will sign the informed consent document (or a mark for those who are illiterate, which will be witnessed by a third party) before any procedures being done specifically for the study. Participants may withdraw consent at any time throughout the course of the trial. A copy of the informed consent document will be given to the participants for

their records. The rights and welfare of the participants will be protected by emphasising to them that the quality of their medical care will not be adversely affected if they decline to participate in this study.

A copy of the signed ICF must be provided to the subject. Signed ICFs must remain in the subjects' study files and be available for verification by the Sponsor or Sponsor representative at any time. The ICF must be signed and dated by the subject before study participation.

20.4 PARTICIPANT AND DATA CONFIDENTIALITY

Confidentiality of each subject is strictly held in trust by the participating Investigators, their staff and the Sponsor(s) and their agents. All records, documents and samples of the subject will be assigned a unique identification number; subjects will not be identified by their names to maintain their confidentiality. This confidentiality is extended to cover testing of biological samples in addition to the clinical information relating to participants. Therefore, the study protocol, documentation, data and all other information generated will be held in strict confidence. No information concerning the study or the data will be released to any unauthorised third party without prior written approval of the Sponsor.

The study monitor, other authorised representatives of the Sponsor, representatives of the IRB/IEC may inspect all documents and records required to be maintained by the Investigator, including but not limited to, medical records (office, clinic or hospital) and pharmacy records for the participants in this study. The clinical study site will permit access to such records.

The study participant's contact information will be securely stored at each clinical site for internal use during the study. At the end of the study, all records will continue to be kept in a secure location for as long a period as dictated by the IRB/IEC and local regulations. Study participant research data, which is for purposes of statistical analysis and scientific reporting, will be transmitted to and stored in the study database. This will not include the participant's contact or identifying information. Individual participants and their research data will be identified by a unique study identification number. The study data entry and study management systems used by clinical sites and by Sponsor/CRO research staff will be secured and password protected. At the end of the study, all de-identified study databases will be archived.

20.4.1 Research Use of Stored Human Samples, Specimens or Data

Microbiology specimens/samples and blood samples will be primarily tested at the local /central laboratory. As part of study protocol, handling and processing of samples (microbiological specimen, including Gram stain slide and unstained slide, isolate(s) with organism identification [to species level], urine for urine antigen testing for detection of *S. pneumoniae* and *L. pneumophilia* and serologic testing for atypicals) at the local laboratory and central laboratory will be provided in the Microbiology Laboratory Procedures Manual.

20.5 FUTURE USE OF STORED SPECIMENS

The samples collected as part of this study may be stored for any future use.

21 STUDY MANAGEMENT

21.1 STUDY MONITORING

Clinical site monitoring is conducted to ensure that the rights and well-being of human subjects are protected, that the reported trial data are accurate, complete and verifiable and that the conduct of the trial is in compliance with the currently approved protocol/amendment(s), with GCP and with applicable regulatory requirement(s).The Sponsor or Sponsor representative will conduct centre visits to inspect study data, subjects' medical records and eCRFs in accordance with current ICH E6 GCP guideline and the respective US, European Union and local regulations and guidelines, as applicable. The Sponsor or Sponsor representative will also be able to review query status remotely, which may warrant additional communication with the Investigator and the study centre's personnel. The Investigator will make available to the Sponsor, or Sponsor representative, source documents, signed ICFs and all other study-related documents. The Investigator will allow the Sponsor or Sponsor representative and applicable regulatory authorities to inspect facilities and records relevant to this study.

21.2 SOURCE DOCUMENTS AND ACCESS TO SOURCE DATA/DOCUMENTS

Source documents may include, but are not limited to, study progress notes, study- or subject-specific e-mail correspondence, computer printouts, laboratory data and recorded data from automated instruments, study drug accountability records. The original signed ICF for each participating subject shall be filed with records kept by the Investigator. All documents produced in this study will be maintained by the Investigator and made available for audits by the Sponsor or Sponsor representative and study site IRBs/IECs and inspections by applicable regulatory authorities.

21.3 QUALITY ASSURANCE AND QUALITY CONTROL

Regular monitoring and an independent audit, if conducted, must be performed according to ICH-GCP. Quality control procedures will be implemented on the data entry system and data quality control checks will be run on the database. Any missing data or data anomalies will be communicated to the site(s) for clarification/resolution. Following written standard operating procedures, the monitors will verify that the clinical trial is conducted and data are generated, documented (recorded) and reported in compliance with the protocol, GCP and the applicable regulatory requirements.

The investigational site will provide direct access to all trial related source data/documents and reports for the purpose of monitoring and auditing by the Sponsor or Sponsor representative and study site IRBs/IECs and inspections by applicable regulatory authorities.

21.4 DATA HANDLING AND RECORD KEEPING

21.4.1 Data Collection and Management Responsibilities

Data collection is the responsibility of the clinical trial staff at the site under the supervision of the site PI. The Investigator is responsible for ensuring the accuracy, completeness, legibility and timeliness of the data reported.

All source documents should be completed in a neat, legible manner to ensure accurate interpretation of data. When making changes or corrections, cross out the original entry with a single line and initial and date the change. DO NOT ERASE, OVERWRITE OR USE CORRECTION FLUID OR TAPE ON THE ORIGINAL.

Data reported in the eCRF derived from source documents should be consistent with the source documents or the discrepancies should be explained and captured.

Clinical data (including AEs, concomitant medications and expected adverse reactions data) and clinical laboratory data will be entered into remote data capture system, a 21 CFR Part 11-compliant data capture system provided by the Sponsor. The data system includes password protection and internal quality checks, such as automatic range checks, to identify data that appear inconsistent, incomplete or inaccurate.

21.4.2 Study Records Retention

Study documents should be retained for a minimum of 2 years after the last approval of a marketing application in an ICH region and until there are no pending or contemplated marketing applications in an ICH region or until at least 2 years have elapsed since the formal discontinuation of clinical development of the investigational product. These documents should be retained for a longer period, however, if required by local regulations. No records will be destroyed without the written consent of the Sponsor, if applicable. It is the responsibility of the Sponsor to inform the Investigator when these documents no longer need to be retained.

21.4.3 Protocol Deviations

A protocol deviation is any noncompliance with the clinical trial protocol or GCP. The noncompliance may be either on the part of the participant, the Investigator or the study site staff. As a result of deviations, corrective actions will be taken by the site and implemented promptly.

It is the responsibility of the site to use continuous vigilance to identify and report deviations as soon as possible. All deviations must be addressed in study source documents. Protocol deviations must be sent to the IRB/IEC as per local guidelines. The site Investigator/study staff is responsible for knowing and adhering to their IRB/IEC requirements.

21.4.4 Publication and Data Sharing Policy

The data generated in this clinical study are the exclusive property of the Sponsor and are confidential. The Sponsor will make all reasonable efforts to publish the results of the study in an appropriate peer-reviewed journal. Authorship on the primary publication of the results from this study will be based on contributions to study design, enrolment, data analysis, and interpretation of results.

21.5 FINANCING AND INSURANCE

The financing and insurance for this study are outlined in the Clinical Trial Agreement.

22 LITERATURE REFERENCES

1. Azmi S, Aljunid SM, Maimaiti N, Ali AA, Muhammad Nur A, De Rosas-Valera M, Encluna J, Mohamed R, Wibowo B, Komaryani K, Roberts C. Assessing the burden of pneumonia using administrative data from Malaysia, Indonesia, and the Philippines. Int J Infect Dis. 2016;49:87-93.
2. Bansal S, Kashyap S, Pal LS, Goel S. Clinical and bacteriological profile of community-acquired pneumonia in Shimla, Himachal Pradesh. Indian J Chest Dis Allied Sci. 2004;46:17–22.
3. Capoor MR, Nair D, Aggarwal P, Gupta B. Rapid diagnosis of community-acquired pneumonia using the Bac T/ Alert 3D System. Braz J Infect Dis. 2006;10:352-356.
4. Centers for Disease Control and Prevention. Available at https://www.cdc.gov/Features/Pneumonia/. Accessed online on 19th March 2018.
5. Centers for Disease Control and Prevention. Available at https://www.cdc.gov/pneumococcal/drug-resistance.html# Accessed online on 05th April 2018.
6. Cilloniz C, Martin-Loeches I, Garcia-Vidal C, San Jose A, Torres A. Microbial etiology of pneumonia: epidemiology, diagnosis and resistance patterns. Int J Mol Sci. 2016;17(12). pii: E2120.
7. Doern GV, Heilmann KP, Huynh HK, Rhomberg PR, Coffman SL, Brueggemann AB. Antimicrobial resistance among clinical isolates of *Streptococcus pneumoniae* in the United States during 1999-2000, including a comparison of resistance rates since 1994-1995. Antimicrob Agents Chemother. 2001;45:1721-1729.
8. Dubois J, Dubois M, Martel J-F. In vitro intracellular activity of a novel lactone ketolide WCK 4873 against resistant Legionella pneumophila. Abstr Sunday-479. Abstr ASM Microbe, Boston, MA. Washington, DC: American Society for Microbiology; 2016.
9. Farrell DJ, Couturier C, Hryniewicz W. Distribution and antibacterial susceptibility of macrolide resistance genotypes in Streptococcus pneumoniae: PROTEKT Year 5 (2003-2004). Int J Antimicrob Agents. 2008;31(3):245-249.
10. Farrell DJ, Jenkins SG, Brown SD, Patel M, Lavin BS, Klugman KP. Emergence and spread of *Streptococcus pneumoniae* with *erm*(B) and *mef*(A) resistance. Emerg Infect Dis. 2005;11:851-858.
11. File TM Jr, Marrie TJ. Burden of community-acquired pneumonia in North American adults. Postgrad Med. 2010;122:130-141.
12. Fine MJ, Auble TE, Yealy DM, Hanusa BH, Weissfeld LA, Singer DE, Coley CM, Marrie TJ, Kapoor WN. A prediction rule to identify low-risk patients with community-acquired pneumonia. N Engl J Med. 1997;336:243-250.
13. Flamm RK, Rhomberg PR, Sader HS. In vitro activity of the novel lactone ketolide nafithromycin (WCK 4873) against contemporary clinical bacteria from a global surveillance program. Antimicrob Agents Chemother. 2017;61(12). pii: e01230-17.
14. Ghoshal AG. Burden of pneumonia in the community. J Assoc Phys India Supplement. 2016;64:1-6.

15. Global Burden of Disease Causes of Death Collaborators. Global, regional, and national age-sex specific mortality for 264 causes of death, 1980–2016: a systematic analysis for the Global Burden of Disease Study 2016. Lancet. 2017;390:1151-1210.
16. Gupta D, Agarwal R, Aggarwal AN, Singh N, Mishra N, Khilnani GC, Samaria JK, Gaur SN, Jindal SK; Pneumonia Guidelines Working Group. Guidelines for diagnosis and management of community- and hospital-acquired pneumonia in adults: Joint ICS/NCCP(I) recommendations. Lung India. 2012;29(Suppl 2):S27-S62.
17. Hung IF, Tantawichien T, Tsai YH, Patil S, Zotomayor R. Regional epidemiology of invasive pneumococcal disease in Asian adults: epidemiology, disease burden, serotype distribution, and antimicrobial resistance patterns and prevention. Int J Infect Dis. 2013;17:e364-e373.
18. Khadanga S, Karuna T, Thatoi PK, Behera SK. Changing bacteriological profile and mortality trends in community acquired pneumonia. J Global Infect Dis. 2014;6:186-188.
19. Kim SH, Song JH, Chung DR, Thamlikitkul V, Yang Y, Wang H, Lu M, So TM, Hsueh PR, Yasin RM, Carlos CC, Pham HV, Lalitha MK, Shimono N, Perera J, Shibl AM, Baek JY, Kang CI, Ko KS, Peck KR; ANSORP Study Group. Changing trends in antimicrobial resistance and serotypes of *Streptococcus pneumoniae* isolates in Asian countries: an Asian Network for Surveillance of Resistant Pathogens (ANSORP) study. Antimicrob Agents Chemother. 2012;56:1418-1426.
20. Kohlhoff SA, Hammerschlag MR. In vitro activities of WCK 4873, a second generation ketolide, against Chlamydia pneumoniae, Abstr Monday-012. ASM Microbe, 16 to 20 June 2016, Boston, MA, USA. Washington, DC: American Society for Microbiology; 2016.
21. Levaquin US Prescribing Information. Janssen Pharmaceutical Companies; February 2017.
22. Millett ER, Quint JK, Smeeth L, Daniel RM, Thomas SL. Incidence of community-acquired lower respiratory tract infections and pneumonia among older adults in the United Kingdom: a population-based study. PLoS One. 2013;8:e75131.
23. Mertz D, Johnstone J. Modern management of community-acquired pneumonia: Is it cost-effective and are outcomes acceptable? Curr Infect Dis Rep. 2011;13:269-277.
24. Oberoi A, Aggarwal A. Bacteriological profile, serology and antibiotic sensitivity pattern of micro-organisms from community acquired pneumonia. JK Science. 2006;8:79-82.
25. Para RA, Fomda BA, Jan RA, Shah S, Koul PA. Microbial etiology in hospitalized North Indian adults with community-acquired pneumonia. Lung India 2018;35:108-115.
26. Parikh PM, Prabhash K, Govind KB, Digumarti R, Pandit S, Banerjee I, Biyani R, Deshmukh A, Doval D, Bhattacharyya GS, Gupta S. Standard operating procedure for audio visual recording of informed consent: an initiative to facilitate regulatory compliance. Indian J Cancer. 2014;51:113-116.

27. Ravindranath M, Raju CH. Validity of pneumonia severity index/pneumonia outcome research trial and Curb-65 severity scoring systems in community acquired pneumonia in Indian setting. Int J Adv Med. 2016;3(2):338-344.
28. Reinert RR. The antimicrobial resistance profile of *Streptococcus pneumoniae*. Clin Microbiol Infect. 2009;15(Suppl 3):7-11.
29. Rodvold KA, Gotfried MH, Chugh R, Gupta M, Friedland FD, Bhatia A. Comparison of plasma and intrapulmonary concentrations of nafithromycin (WCK 4873) in healthy adult subjects. Antimicrob Agents Chemother. 2017;61(9):e01096-17.
30. Rozenbaum MH, Mangen MJ, Huijts SM, van der Werf TS, Postma MJ. Incidence, direct costs and duration of hospitalization of patients hospitalized with community-acquired pneumonia: A nationwide retrospective claims database analysis. Vaccine. 2015;33:3193-3199.
31. Schedule Y (Rules 122A, 122B, 122D, 122DA, 122DAA and 122E). Requirements and guidelines for permission to import and/or manufacture new drugs for sale or to undertake clinical trials. Drugs and Cosmetics Rules, 1945. http://cdsco.nic.in/html/D&C_Rules_Schedule_Y.pdf. Accessed online on 22 June 2018.
32. Shah BA, Ahmed W, Dhobi GN, Shah NN, Khursheed SQ, Haq I. Validity of pneumonia severity index and CURB-65 severity scoring systems in community acquired pneumonia in an Indian setting. Indian J Chest Dis Allied Sci. 2010a;52(1):9-17.
33. Shah BA, Singh G, Naik MA, Dhobi GN. Bacteriological and clinical profile of Community acquired pneumonia in hospitalized patients. Lung India. 2010b;27(2):54-57.
34. Song JH, Lee NY, Ichiyama S, Yoshida R, Hirakata Y, Fu W, Chongthaleong A, Aswapokee N, Chiu CH, Lalitha MK, Thomas K, Perera J, Yee TT, Jamal F, Warsa UC, Vinh BX, Jacobs MR, Appelbaum PC, Pai CH. Spread of drug-resistant *Streptococcus pneumoniae* in Asian countries: Asian Network for Surveillance of Resistant Pathogens (ANSORP) Study. Clin Infect Dis. 1999;28:1206-1211.
35. Song JH, Oh WS, Kang CI, Chung DR, Peck KR, Ko KS, Yeom JS, Kim CK, Kim SW, Chang HH, Kim YS, Jung SI, Tong Z, Wang Q, Huang SG, Liu JW, Lalitha MK, Tan BH, Van PH, Carlos CC, So T; Asian Network for Surveillance of Resistant Pathogens Study Group. Epidemiology and clinical outcomes of community-acquired pneumonia in adult patients in Asian countries: A prospective study by the Asian network for surveillance of resistant pathogens. Int J Antimicrob Agents. 2008;31:107-114.
36. Sutcliffe J, Tait-Kamradt A, Wondrack L. *Streptococcus pneumoniae* and *Streptococcus pyogenes* resistant to macrolides but sensitive to clindamycin: a common resistance pattern mediated by an efflux system. Antimicrob Agents Chemother. 1996;40:1817-1824.
37. Torumkuney D, Chaiwarith R, Reechaipichitkul W, Malatham K, Chareonphaibul V, Rodrigues C, Chitins DS, Dias M, Anandan S, Kanakapura S, Park YJ, Lee K, Lee H, Kim JY, Lee Y, Lee HK, Kim JH, Tan TY, Heng YX, Mukherjee P, Morrissey I. Results from the Survey of Antibiotic Resistance (SOAR)

2012–14 in Thailand, India, South Korea and Singapore. J Antimicrob Chemother. 2016;71(Suppl 1):i3–i19.

38. Udwadia ZF, Doshi AV, Joshi JM. Etiology of community acquired pneumonia in India. Eur Respir J. 2003;22:5445.
39. US Department of Health and Human Services, National Institutes of Health, National Cancer Institute. Common Terminology Criteria for AEs (CTCAE) Version 4.03. Available at: http://www.oncology.tv/SymptomManagement/NationalCancerInstituteUpdatesCTCAEtov403.aspx Accessed online on 09th April 2018.
40. Venditti M, Falcone M, Corrao S, Licata G, Serra P; Study Group of the Italian Society of Internal Medicine. Outcomes of patients hospitalized with community-acquired, health care-associated, and hospital-acquired pneumonia. Ann Intern Med. 2009;150:19-26.
41. Vigg A. Severe community acquired pneumonia (SCAP). Apollo Medicine. 2016;13(1):17-19.
42. Waites KB, Crabb DM, Duffy LB. In vitro activities of investigational ketolide WCK 4873 (nafithromycin) and other antimicrobial agents against human mycoplasmas and ureaplasmas, Abstr Sunday-480. Abstr ASM Microbe, Boston, MA. Washington, DC: American Society for Microbiology; 2016.
43. Welte T, Torres A, Nathwani D. Clinical and economic burden of community-acquired pneumonia among adults in Europe. Thorax. 2012;67:71-79.
44. Whitney CG, Farley MM, Hadler J, Harrison LH, Lexau C, Reingold A, Lefkowitz L, Cieslak PR, Cetron M, Zell ER, Jorgensen JH, Schuchat A; Active Bacterial Core Surveillance Program of the Emerging Infections Program Network. Increasing prevalence of multidrug-resistant *Streptococcus pneumoniae* in the United States. N Engl J Med. 2000;343:1917-1924.
45. Raul E. Isturiz a*, Carlos M. Luna b, Julio Ramirez c; Clinical and economic burden of pneumonia among adults in Latin America. International Journal of Infectious Diseases 14 (2010): e852–e856.

23 APPENDICES

23.1 Appendix I: Allowed and Disallowed Prior Antibiotics

**Allowed Antibiotics (One dose within 72 h prior to randomisation*)**	**Disallowed Antibiotics**
Penicillins	
Amoxicillin, Nafcillin,<br>Amoxicillin-Clavulanate, Oxacillin<br>Amoxicillin-Sulbactam, Penicillin-G or -V<br>Ampicillin, Piperacillin,<br>Ampicillin-Sulbactam, Piperacillin-Tazobactam<br>Dicloxacillin, Ticarcillin-Clavulanate	Benzathine/Penicillin-G Procaine
Cephalosporins	
Cefaclor, Cefpodoxime,<br>Cefadroxil, Cefprozil,<br>Cefazolin, Ceftaroline,<br>Cefdinir, Ceftazidime,<br>Cefepime, Ceftibuten,<br>Cefixime (200 mg), Cefuroxime,<br>Cefditoren, Cephalexin,<br>Cefotaxime, Loracarbef	Cefixime (400 mg) Ceftriaxone
Carbapenems	
Doripenem,<br>Imipenem,<br>Meropenem	Ertapenem
Glycopeptides	
Televancin,<br>Vancomycin	Dalbavancin,<br>Oritavancin
Fluoroquinolones	
Ciprofloxacin	Levofloxacin,<br>Moxifloxacin
Macrolides	
Clarithromycin,<br>Erythromycin	Azithromycin,<br>Clarithromycin XL**
Tetracyclines	
Doxycycline (100 mg),<br>Minocycline	Doxycycline (200 mg),<br>Minocycline Extended Release,<br>Tigecycline,<br>Omadacycline
Oxazolidinones	
Linezolid	Tedizolid
Miscellaneous	
Clindamycin,<br>Metronidazole,<br>Trimethoprim-sulfamethoxazole/Co-trimoxazole	

*Prior (within 72 h prior to randomisation) administration of potentially effective systemic antibacterial therapy is an exclusion criterion (Section 12.3); however, subjects may be eligible for the study despite prior anti-microbial therapy if they received a single dose of a single short-acting systemic antibiotic within 72 h prior to randomisation. For the purposes of this protocol, short-acting is defined as having a dosage frequency of more than once a day. If a subject received a prior short-acting systemic antibiotic that is not listed here, the Investigator must contact the Medical Monitor to ensure subject eligibility.
** Extended release

23.2 Appendix II: PORT Score Calculation

**Subject Characteristic**	**Point Assignment**
Age	
Male	Age (years)
Female	Age (years) -10
Nursing home resident[1]	+10
Coexisting illnesses	
Neoplastic disease[2]	+30
Liver disease[3]	+20
Congestive heart failure[4]	+10
Cerebrovascular disease[5]	+10
Renal disease[6]	+10
Physical-examination findings	
Altered mental status[7]	+20
Respiratory rate ≥ 30/minute	+20
Systolic blood pressure < 90 mm Hg	+20
Temperature < 35°C (95°F) or ≥ 40°C (104°F)	+15
Pulse ≥ 125/minute	+10
Laboratory and radiographic findings	
Arterial pH < 7.35[8]	+30
Blood urea nitrogen ≥ 30 mg/dL (11 mmol/L)[6]	+20
Sodium < 130 mmol/L	+20
Glucose ≥ 250 mg/dL (14 mmol/L)	+10
Haematocrit < 30%	+10
Partial pressure of arterial oxygen < 60 mm Hg or oxygen saturation < 90% (by pulse oximetry)[8]	+10
Pleural effusion	+10
PORT Score	Sum of numbers above

**PORT Risk Class**	**PORT Score**
I (ineligible for study)	0-50
II (eligible)	51-70

III (eligible)	71-90
IV (score of 91-105 eligible and 106-130 ineligible)	91-130
V (ineligible for study)	≥ 131

1. Subjects that reside in a nursing home are excluded from the study and should not be enrolled per Exclusion Criterion #1.
2. Neoplastic disease is defined as any cancer, except basal or squamous cell cancer of the skin that was active at the time of presentation or diagnosed within one year of presentation. Subjects with neoplastic lung disease are excluded from the study and should not be enrolled per Exclusion Criterion #7.
3. Liver disease is defined as a clinical or histologic diagnosis of cirrhosis or another form of chronic liver disease, such as chronic active hepatitis. Subjects with liver test abnormalities or evidence of end-stage liver disease as defined in Exclusion Criterion #8 should not be enrolled.
4. Congestive heart failure is defined as systolic or diastolic ventricular dysfunction documented by history, physical examination, chest radiograph, echocardiogram, multiple gated acquisition scan or left ventriculogram. Subjects with acute congestive heart failure are excluded from the study and should not be enrolled per Exclusion Criterion #11.
5. Cerebrovascular disease is defined as a clinical diagnosis of stroke or transient ischaemic attack or stroke documented by magnetic resonance imaging or computed tomography.
6. Renal disease is defined as a history of chronic renal disease or abnormal blood urea nitrogen and creatinine concentrations documented in the medical record. Subjects with compromised renal function are excluded from the study and should not be enrolled per Exclusion Criterion #8.
7. Altered mental status is defined as disorientation with respect to person, place or time that is not known to be chronic, stupor or coma.
8. For subjects without an optional arterial blood gas at Screening, no points will be added for pH or partial pressure of arterial oxygen; however, oxygen saturation results should be used in place of partial pressure of arterial oxygen.

Reference: Fine et al, 1997.

23.3 Appendix III: CABP Symptom Severity Guidance for Investigator Assessment

CABP Symptom	Absent	Mild	Moderate	Severe
**Dyspnoea (SOB)**	No CABP-associated SOB; or return to pre-CABP baseline SOB	SOB with only strenuous activity but it does not interfere with subject's usual daily activities; or mild SOB above pre-CABP baseline	SOB with usual activities and it does interfere with some of the subject's usual daily activities; or moderate SOB above pre-CABP baseline	SOB with minimal exertion or at rest and it limits most of the subject's usual daily activities; or severe SOB above pre-CABP baseline
**Cough**	Resolution of cough; or return to pre-CABP baseline cough	Cough present, infrequent, and it does not interfere with subject's usual daily activities; or mild cough above pre-CABP baseline	Cough present, frequent, and it does interfere with some of the subject's usual daily activities; or mild cough above pre-CABP baseline	Cough is present throughout the day and night; it limits most of the subjects' usual daily activities and sleep patterns; or mild cough above pre-CABP baseline
**Production of Purulent Sputum**	No production of purulent sputum; or return to pre-CABP baseline productive cough	Production of small amount of purulent sputum; or minimal amount of sputum production above pre-CABP baseline	Production of moderate amount of purulent sputum; or moderate amount of sputum production above pre-CABP baseline	Production of large amount of purulent sputum; or large amount of sputum production above pre-CABP baseline
**Pleuritic Chest Pain**	No CABP-associated pleuritic chest pain	CABP-associated pleuritic chest pain present occasionally with deep breathing but it does not interfere with subject's usual daily activities	CABP-associated pleuritic chest pain is present with normal breaths, and it does interfere with the subject's usual daily activities	CABP-associated pleuritic chest pain is present at rest and/or with shallow breathing, and it limits most of the subject's usual daily activities

CABP = community-acquired bacterial pneumonia; SOB = shortness of breath

23.4 Appendix IV: Safety Laboratory Tests Conducted by the Central Laboratory

**Haematology:**	**Chemistry (Serum Concentrations):**
• Haemoglobin<br>• Haematocrit<br>• Erythrocyte count<br>• Mean red blood cell volume<br>• Mean red blood cell haemoglobin<br>• Mean red blood cell haemoglobin concentration<br>• Leukocyte count<br>• Neutrophils (including immature neutrophils [bands] and absolute neutrophil count)<br>• Lymphocytes<br>• Monocytes<br>• Eosinophils<br>• Basophils<br>• Platelets<br>• Reticulocytes<br><br>**Urinalysis** (only if local urine dipstick test abnormal and deemed clinically significant by the Investigator; see Section 11.12):<br>• Specific gravity<br>• pH<br>• Protein<br>• Glucose<br>• Ketones<br>• Bilirubin<br>• Occult blood<br>• Nitrites<br>• Urobilinogen<br>• Leucocyte esterase	• Glucose<br>• Calcium<br>• Albumin<br>• Total protein<br>• Sodium<br>• Potassium<br>• Carbon dioxide<br>• Chloride<br>• Blood urea nitrogen<br>• Creatinine<br>• Alkaline phosphatase<br>• Alanine aminotransferase<br>• Aspartate aminotransferase<br>• Total and direct bilirubin<br>• Magnesium<br>• Lactate dehydrogenase<br>• Phosphorus<br>• Uric acid<br>• Creatine kinase<br>• Gamma-glutamyl transferase<br>• β-Human chorionic gonadotropin for females

Protocol Amendment Administrative Letter

Date: 11-Nov-2020

Protocol Number: W-4873-301

Protocol Title: A Phase III, Randomised, Multicentre, Double-Blind, Comparative Study to Determine the Efficacy and Safety of Oral Nafithromycin Versus Oral Moxifloxacin in the Treatment of Community-Acquired Bacterial Pneumonia (CABP) in Adults

Protocol Date: Protocol Amendment 03 dated 5 February 2019

Dear Investigator:

This letter is being sent to sites to provide following clarifications to Protocol W-4873-301. This letter should be submitted to your Institutional Review Board/Ethics Committee as appropriate. Please retain a copy in your site Trial Master File.

Section No.	Page No.	Clarification to Text	Rationale for Change
Section 4, Section 20.1, Section 20.3.2 and Section 22	14, 89, 95	In the referred sections of the protocol, the study is stated to be conducted in accordance with Good Clinical Practice (GCP) as required by the following:<br>Schedule Y guidelines (rules 122A, 122B, 122D, 122DA, 122DAA and 122E). Requirements And Guidelines For Permission To Import And / Or Manufacture Of New Drugs For Sale Or To Undertake Clinical Trials, Drugs and Cosmetics Rules, 1945, India<br>Given the revision of local regulations/guidance, the conduct of the study should now be considered in conformity with the New Drugs and Clinical Trials, Rules, 2019 of India.	Revision in local regulations
Section 6 and Section 12.3	23, 28 and 54	The referred sections of the protocol mention Collection of creatinine clearance (CrCl). It is being clarified that the creatinine clearance (CrCl) should be calculated using the Cockcroft-Gault equation.	Comparability of data

Protocol Amendment Administrative Letter

Section 16.1	71	The referred section of the protocol states Sputum Resubmission Criteria. It is being clarified that the collection of additional sputum/tracheal aspirate samples (as early as possible and not later than 24 h after the first dose of the study drug) could be attempted only up on timely (prior to Day 2 dose administration) confirmation of protocol specified sputum quality by the central laboratory, as applicable.	Clarification relevant to timing of confirmation of sputum quality criteria
Section 6 and Section 14.10.1	28 and 68	The referred sections of the protocol define complete physical exam and assessment of severity of the subject's CABP symptoms of dyspnoea, cough, production of purulent sputum and pleuritic chest pain, based on the CABP Symptom Severity Guidance for Investigator Assessment (Appendix III).<br>It is being clarified that the assessment of severity of the subject's CABP symptoms (as defined in the protocol) could also be conducted (in-person or telephonic) at the follow-up (FU-Day 31) visit in order to assess for clinical relapse or recurrence of CABP.	Clarification to allow evaluation of clinical relapse or recurrence of CABP at FU visit
Section 23.4	101	It is being clarified that the Safety Laboratory Tests Conducted by the Central Laboratory will include Total and direct bilirubin.	Administrative clarification
Section 23.2	99	In the referred section, at the footnotes (# 2, 3, 4 and 6) of the table for PORT score calculation, the exclusion criteria number have be inaccurately referred as 7, 8, 11 and 8. This is to clarify accurate exclusion criteria numbers as 6, 7, 10 and 7.	Rectification of typographical error
Section 6 and 13.1.5.1	24, 59 and 60	It is being clarified that the dose of study drugs as stated in the referred section of the protocol should preferably be administered with food.	Clarification for dosing

The above mentioned clarifications are provided for logistic/administrative purposes. These clarifications do not significantly impact the safety, scope, or scientific quality of the protocol If there is any future protocol amendment then these points will be incorporated in the protocol amendment document.

Dr Mushtaque Mastim 11 November 2020

Name **Signature & Date**

WOCKHARDT

Protocol Amendment Administrative Letter

Date: 24-Nov-2022

Protocol Number: W-4873-301

Protocol Title: A Phase III, Randomised, Multicentre, Double-Blind, Comparative Study to Determine the Efficacy and Safety of Oral Nafithromycin versus Oral Moxifloxacin in the Treatment of Community-Acquired Bacterial Pneumonia (CABP) in Adults

Protocol Date: Protocol Amendment 03 dated 5 February 2019

Dear Investigator:

This letter is being sent to sites to provide following update to Protocol W-4873-301. This letter should be submitted to your Institutional Review Board/Ethics Committee (IRB / EC) as appropriate. With respect to this communication, please retain the following in your site Trial Master File:

1. A copy of this letter
2. Submission letter to IRB / EC
3. Acknowledgment received from IRB / EC

Please share scan copy of IRB / EC acknowledgement with your site monitor.

**Section No.**	**Page No.**	**Clarification to Text**	**Rationale for Change**
1. Title Page and 2. Protocol Signature page	1, 2	Change of Sponsor Medical Monitor from Dr. Mushtaque Mastim<br>to<br>Dr. Ashoka Singh	Dr. Mastim is no longer associated with Wockhardt.

The above mentioned update is provided for administrative purposes. This update does not significantly impact the safety, scope, or scientific quality of the protocol. If there is any future protocol amendment then these points will be incorporated in the protocol amendment document.

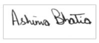

Signed by Ashima Bhatia, Date: 24-Nov-2022 03:29 PM +05:30, DocID : 8c0229f1-7516-4771-9664-f6f31a93c348.

Dr. Ashima Bhatia
Head - Global Clinical Development
Wockhardt

Image

Image

W-4873-301- Protocol Amendment 03 Administrative Letter dated 16Nov2022 _

Document information

Document ID :
8c0229f1-7516-4771-9664-f6f31a93c348

Status: Signed

Signing Process Details

Signing Flow:	Parallel
Uploaded On:	24-Nov-2022 03:10 PM
Total Pages:	1
Total Signers:	1
Total Observers:	0
Total Approvers:	0
Signatures Provided:	E-Signature
Time Zone:	(UTC+05:30) Chennai, Kolkata, Mumbai, New Delhi

Document Owner Information

Name:	Glenith Silvalobo
Email Address:	

GSilvalobo@wockhardt.com

IP Address:	192.168.230.113
Browser:	Chrome 107.0.0.0
Lat/Long:	Details not shared.
Device/OS:	Windows 8.1

AB Ashima Bhatia (Signer)

Email Address:	ABhatia@wockhardt.com
IP Address:	49.36.181.234
Browser:	Firefox 107.0
Device/OS:	Windows 10
Lat/Long:	28 34 54 N, 77 3 37 E

Signatures Provided
E-Signature(1)

Signature Timestamp
24-Nov-2022 03:30 PM

There are no Observers

Blockchain Timestamping

BlockID:	6747
Timestamped on:	24-Nov-2022 03:30 PM
Signed Document hash:	cb478e5beb22931a72778c682ac4b04be7b4020a84c601c26206c470e3268934
Completed on:	24-Nov-2022 03:30 PM
Blockchain hash:	0xfd6c6c7c5338b3526eaea52232c60faba000f46417e8a63d1ff298ff6ee47f93
