# Supplementary material for: Efficacy and safety of a 3-day once-daily regimen of oral nafithromycin in comparison to oral moxifloxacin for the treatment of community-acquired bacterial pneumonia in adults: a phase III, randomized, double-blind controlled trial
Source: Lancet Reg Health Southeast Asia. 2025 Sep 23;41:100666. doi: 10.1016/j.lansea.2025.100666 (PMC12494923; doi:10.1016/j.lansea.2025.100666)
Supplement: Statistical analysis plan [file mmc2.pdf]

STATISTICAL ANALYSIS PLAN
CLINICAL STUDY PROTOCOL No.: W-4873-301

A Phase III, Randomised, Multicentre, Double-Blind, Comparative Study to Determine the Efficacy and Safety of Oral Nafithromycin versus Oral Moxifloxacin in the Treatment of Community-Acquired Bacterial Pneumonia (CABP) in Adults

SAP Version: 1.0
Date: 22 MAY 2023

SIGNATURE PAGE

We, the undersigned, declare that we have thoroughly reviewed this guideline for completeness and accuracy with the Protocol Requirements, CRF details, Database, SOPs and ICH-GCP.

Prepared by Sign and Date: Swapnil Patil — Digitally signed by Swapnil Patil Date: 2023.05.30 11:25:32 +05'30'

Swapnil Patil
(Associate Biostatistician)

Reviewed by Sign and Date: Vidya Thombare — Digitally signed by Vidya Thombare Date: 2023.05.30 12:02:11 +05'30'

Vidya Thombare
(Biostatistician-I)

Reviewed and Approved by Sign and Date: [signature] 31 May 2023

Sponsor Representative
Wockhardt Limited

1. TABLE OF CONTENTS

1. TABLE OF CONTENTS 3
2. REVISION HISTORY 5
3. LIST OF ABBREVIATIONS 6
4. INTRODUCTION 9
5. OBJECTIVES 10
5.1 PRIMARY OBJECTIVES 10
5.2 SECONDARY OBJECTIVES 10
6. SUMMARY OF THE STUDY DESIGN 11
7. RANDOMISATION 12
8. STUDY SCHEDULE OF EVENTS 13
9. SAMPLE SIZE 17
10. SAMPLE SIZE RE-ESTIMATION 18
11. GENERAL STATISTICAL METHODOLOGY 19
12. ANALYSES SETS 20
12.1 ALL ENROLLED SUBJECTS 20
12.2 INTENT-TO-TREAT (ITT) ANALYSES SET 20
12.3 MODIFIED INTENT TO TREAT (MITT) ANALYSES SET 20
12.4 SAFETY ANALYSES SET 20
12.5 EXPANDED MICROBIOLOGICAL MODIFIED INTENT-TO-TREAT (Expanded mMITT Analyses set) ANALYSES SET 20
12.6 MICROBIOLOGICAL MODIFIED INTENT-TO-TREAT (mMITT) ANALYSES SET 20
12.7 CLINICALLY EVALUABLE (CE) ANALYSES SET 20
12.8 PHARMACOKINETIC (PK) ANALYSIS SET 21
13. DISPOSITION 22
14. PROTOCOL DEVIATION 23
15. BASELINE CHARACTERISTICS 25
15.1 Demographics and Baseline Characteristics 25
15.2 Medical History, Co-Morbid Conditions and Markers of CABP 25
15.3 Gram Stain and Microbiological Results at Baseline 25
16. PRIOR AND CONCOMITANT MEDICATIONS, NON-DRUG THERAPIES AND PROCEDURES 26
17. TREATMENT EXPOSURE 27
18. STUDY EFFICACY VARIABLES / ENDPOINTS 28
18.1 Primary Efficacy Endpoints 28
18.2 Secondary Efficacy Endpoints 28

18.3 Safety Endpoints 29
19. EFFICACY ANALYSES 30
19.1 Primary Efficacy Analysis 30
19.2 Sensitivity Analysis 30
19.3 Secondary Efficacy Analysis 31
19.4 Subgroup Analysis 33
20. SAFETY ANALYSES 35
20.1 Adverse Events 35
20.2 Clinical Laboratory Evaluations 35
20.3 Vital Signs 39
20.4 Electrocardiogram (ECG) 39
21. PK ANALYSIS 41
22. MULTIPLE COMPARISONS 42
23. METHODS FOR HANDLING MISSING DATA 43
24. PLANNED ANALYSIS 44
24.1 Interim Analysis 44
24.2 Final Analysis 44
25. SOFTWARE USED FOR ANALYSIS 45
26. CHANGES FROM STUDY PROTOCOL 46
27. APPENDIX 46

2. REVISION HISTORY

SAP Number	Version Number	Description
W-4873-301-SAP-22-MAY-2023	1.0	Initial Document

3. LIST OF ABBREVIATIONS

AE	Adverse Event
ALP	Alkaline Phosphatase
ALT	Alanine Aminotransferase
AST	Aspartate Aminotransferase
ATC	Anatomical Therapeutical Chemical
BAL	Bronchoalveolar Lavage
BMI	Body Mass Index
BUN	Blood Urea Nitrogen
CABP	Community Acquired Bacterial Pneumonia
CE	Clinically Evaluable
CH PCS	Clinically High PCS
CI	Confidence Interval
CL PCS	Clinically Low PCS
CRF	Case Report Form
CRO	Contract Research Organization
CT	Computed Tomography
CV	Coefficient of Variation
CXR	Chest X Ray
ECG	Electrocardiogram
Ecrf	Electronic Case Report Form
E-mMITT	Expanded Microbiologically Modified Intent-to-Treat
EOT	End-of-Treatment
EUCAST	European Committee on Antimicrobial Susceptibility Testing
FDA-STIC	US Food and Drug Administration Susceptibility Test Interpretive Criteria
FU	Follow-up
GCP	Good Clinical Practice
HIV	Human Immunodeficiency Virus
ICF	Informed Consent Form
ICH	International Council for Harmonisation
INR	International Normalized Ratio

IP	Investigational Product
ITT	Intent-to-Treat
IXRS	Interactive Voice/Web Response System
LDH	Lactate Dehydrogenase
LLN	Lower Limit of Normal
MCH	Mean Corpuscular Hemoglobin
MCHC	Mean Corpuscular Hemoglobin Concentration
MCV	Mean Corpuscular Volume
MDR	Multi-Drug Resistant
MedDRA	Medical Dictionary for Regulatory Activities
MIC	Minimum Inhibitory Concentration
MIC_{50}	50th Percentile of the MIC Distribution
MIC_{90}	90th Percentile of the MIC Distribution
MITT	Modified Intent-to-Treat
mMITT	Microbiological Modified Intent-to-Treat
MTBC	Mycobacterium Tuberculosis Complex
N/A	Not Applicable
NS	Non-Significant
PCS	Potentially Clinically Significant
PN	Preferred Name
PDs	Protocol Deviations
PH	Potential of Hydrogen
PK	Pharmacokinetic(s)
PO	Orally, Per os or by Mouth
PORT	Pneumonia Outcomes Research Team
PP	Per-Protocol
PT	Preferred Term
PT	Prothrombin Time
aPTT	Activated partial thromboplastin time
QTcF	QT Interval Corrected Using Fridericia's Formula
q24h	Every 24 Hours

RR	Respiratory Rate
S	Significant
SAE	Serious Adverse Event
SAP	Statistical Analysis Plan
SAS	Statistical Analysis System
SD	Standard Deviation
SIRS	Systemic Inflammatory Response Syndrome
SOC	System Organ Class
SOP	Standard Operating Procedure
TB	Tuberculosis
TEAE	Treatment Emergent Adverse Event
TLF	Table listing and Figure
TOC	Test of Cure
ULN	Upper Limit of Normal
USA	United States of America
USFDA	United States Food and Drug Administration
WBC	White Blood Cell
WHODD	World Health Organization Drug Dictionary

4. INTRODUCTION

This Statistical Analysis Plan (SAP) is based on the final Clinical Study Protocol W-4873-301 dated 05-Feb-2019. The SAP provides details on the planned statistical methodology for the analysis of the study data.

This SAP describes the study endpoints, derived variables, anticipated data transformations and other details of the analyses not provided in the study protocol.

The following documents were reviewed in preparation of this SAP:

- Final Clinical study Protocol W-4873-301 dated 05-Feb-2019.
- Final eCRF W-4873-301 Version 4.0 dated 09 Nov 2022.

5. OBJECTIVES

5.1 PRIMARY OBJECTIVES

- To demonstrate that oral nafithromycin is non-inferior to oral moxifloxacin in the clinical response at Day 4 in the Modified Intent-to-Treat (MITT) analyses set
- To assess overall safety of oral nafithromycin in the safety analyses set

5.2 SECONDARY OBJECTIVES

- To assess the clinical response at Day 4 in the Microbiological Modified Intent-to-Treat (mMITT) and Clinically Evaluable (CE) analyses sets
- To assess the clinical outcome at End of Treatment (EOT) in the MITT, mMITT and CE analyses sets
- To assess the clinical outcome at Test of Cure (TOC) in the MITT, mMITT and CE analyses sets
- To assess re-admission to the hospital (or admission to the hospital if not previously hospitalised) for any reason before Follow-Up (FU) (Day 31 ± 4 days) in the MITT analyses set
- To determine the PK of oral nafithromycin (in PK analysis set)
- To assess by-pathogen clinical response at Day 4 and by-pathogen clinical outcome at TOC in the mMITT analyses set

6. SUMMARY OF THE STUDY DESIGN

This is a Phase III, prospective, multicentre, randomised, double-blind, comparative efficacy and safety study of oral nafithromycin versus oral moxifloxacin for the treatment of male and female adults with CABP. Subjects providing informed consent and meeting all study eligibility criteria will be enrolled in the study and randomised in a 1:1 ratio to either of the following 2 treatment arms:

- Nafithromycin 800 mg (two 400-mg tablets) orally (PO) every 24 hours (q24h) for 3 days; subjects will receive matching placebo PO q24h on Day 4 through EOT (2 tablets) and matching moxifloxacin placebo PO q24h, on Day 1 through EOT (1 capsule), to maintain the blind (2 tablets and 1 capsule in total).
- Moxifloxacin 400 mg (1 capsule: Over-encapsulated tablet) PO q24h for 7 days; subjects will receive matching nafithromycin placebo PO q24h on Day 1 through EOT (2 tablets), to maintain the blind (2 tablets and 1 capsule in total).

Pharmacokinetic sampling:

Blood samples for PK analysis will be collected from subjects on Day 1, Day 3 and Day 4 at sites where PK sampling is possible. Time points for PK sample collection will be as follows:

- A pre-dose PK sample will be collected within 10 min before dosing on Day 3.
- Post-dose PK samples will be collected at 2-4 h (Day 1 and Day 3) and 24-28 h (On Day 4, i.e. 24-28 h after Day 3 dose). Subjects who have been hospitalized are also required to have a post-dose PK sample at 6-10 h on Day 1 and Day 3.

Subjects shall attend the following scheduled clinic visits:

- **VISIT1:** Screening (Within 24 hrs prior to randomisation)
- **VISIT2:** Randomisation includes PK sampling
- **VISIT3:** Day 2
- **VISIT4:** Day 3 includes PK sampling
- **VISIT5:** Day 4 includes PK sampling
- **VISIT6:** Day 5
- **VISIT7:** Day 6
- **VISIT8:** Day 7+2 (EOT)
- **VISIT9:** Day 15±4 (TOC)
- **VISIT10:** Day 31±4 (FU)

Subjects may attend unscheduled clinic visits.

7. RANDOMISATION

Subjects will be randomised using block randomisation, stratified by Pneumonia Outcomes Research Team (PORT) Risk Class (II vs. III/IV) to avoid bias while assigning the treatment arm. Subjects will be randomly assigned in blinded manner to 1 of the 2 treatment arms using an Interactive web recognition system (IWRS) in the ratio of 1:1. Enrolment of Pneumonia Outcome Research Team (PORT) Risk Class II will be capped at 60% and enrolment of subjects with allowed prior systemic antibiotic use will be capped initially at 25% (subject to change during study conduct).

8. STUDY SCHEDULE OF EVENTS

Visits	Screening Visit (Day -1)[a]	Day 1[b]	Day 2	Day 3	Day 4	Day 5[w]	Day 6[w]	EOT Day 7 (+ 2 days)[c]	TOC Day 15 (± 4 days)[d]	FU Day 31 (± 4 days)[e]
Informed Consent[f]	√									
Medical history and demography	√									
Physical exam, including CABP symptom severity[g]	√	√	√	√	√			√	√	
Vital signs, including oximetry[h]	√	√	√	√	√			√	√	
12-Lead ECG[i]	√			√				√	√i	√i
CXR or chest CT scan[j]	√									
PORT score calculation[k]	√									
Inclusion/exclusion criteria	√	√								
Randomisation		√								
Laboratory assessments										
Haematology, coagulation, serum chemistry[l]	√			√				√	√	√
Serology for HIV[l]	√									
Atypical pathogen serology[m]	√								√	√
Urine tests[n]	√									
Urinary antigen testing[o]	√									
Pregnancy test[p]	√								√	
Respiratory specimen Gram stain/culture[q]	√	As clinically indicated								
Blood culture[r]	√	Repeat the blood cultures as necessary until negative blood cultures are obtained								
Blood for PK sampling[s]		√		√	√					
Study drug administration and accountability[t]		√	√	√	√	√	√	√		
Adverse Events[u]	√	√	√	√	√	√	√	√	√	√

**Prior and concomitant medications**[v]	√	√	√	√	√	√	√	√	√	√
**Clinical outcome assessment (by the investigator)**								√	√	

Abbreviations: AE = adverse event; ALT = alanine aminotransferase; AST = aspartate aminotransferase; CABP = community-acquired bacterial pneumonia; CT= computed tomography; CXR = chest X-ray; ECG = electrocardiogram; EOT = end-of-treatment; FU = follow-up; PK = pharmacokinetic; PORT = Pneumonia Outcomes Research Team; TOC = Test of Cure

[a] Following signing of the informed consent form, all Screening evaluations should be completed within 24 h before randomisation.

[b]Day 1 is the first day of study drug administration. Subsequent study days are consecutive calendar days. If feasible, Screening and randomisation procedures (Screening visit and Day 1) can be performed on the same day. Standard of care data from within 24 h (laboratory) and 48 h (radiology) before randomisation can be used as Screening visit procedures.

[c]EOT is to be conducted on or within 2 days after Day 7. EOT Visit procedures should also be conducted for any premature withdrawal from the study or the study drug.

[d]TOC is to be conducted on Day 15 ± 4 days.

[e]Best possible efforts should be made to conduct an in-person FU Visit on Day 31 ± 4 days. In the event that an in-person visit is not possible due to any reason, assessment may be conducted through a telephone contact for subjects who were considered to be Clinical Cures and had no AEs or clinically significant laboratory or ECG abnormalities noted at or after the TOC Visit. Such logistical reasons should be documented. For the subjects who had AEs or clinically significant laboratory or ECG abnormalities noted at or after the TOC Visit, an in-person follow-up visit must be conducted.

[f] Written and signed informed consent must be obtained before any protocol assessment is performed.

[g] Complete physical exam—consisting of general appearance, the skin, eyes, ears, nose, throat, lungs, heart, abdomen, back, extremities, lymph nodes, vascular and neurological exams—will be conducted at Screening and daily between Day 1 and Day 4 (whether inpatient or outpatient), EOT and TOC. As part of the complete physical exam, the Investigator should assess the severity of the subject's CABP symptoms of dyspnoea, cough, production of purulent sputum and pleuritic chest pain, based on the CABP Symptom Severity Guidance for Investigator Assessment (Appendix III). At screening visit, assessment of premorbid symptoms (approximately 7 days prior to onset of current CABP symptoms) should be conducted.

[h]Vital signs—including body temperature (oral, rectal or tympanic), blood pressure, pulse rate, respiratory rate and pulse oximetry—will be collected at Screening and daily between Day 1 and Day 4 (whether inpatient or outpatient), EOT and TOC. Height, weight and creatinine clearance (CrCl) will also be collected at the Screening visit.

[i]A 12-lead ECG will be performed at Screening, Day 3 and EOT. A 12-lead ECG will be performed at TOC or FU if prior ECG(s) showed any clinically significant abnormality.

[j]Subjects must have a confirmatory CXR or chest CT scan consistent with acute bacterial pneumonia within 48 h before randomisation.

[k]Subjects with a PORT score of 51 to 105 (PORT Risk Class of II, III or IV) are eligible for enrolment.
[l]At Screening, local laboratory evaluations required for assessing subject eligibility include serum aminotransferase (ALT and AST) and total bilirubin levels, serum creatinine and blood urea nitrogen (or urea), peripheral white blood cell (WBC) count, absolute neutrophil count, coagulation and immature neutrophil percentage and serology for HIV. Blood will be collected for central laboratory testing at the Screening, Day 3, EOT and TOC visits. Local laboratory evaluations will be conducted at FU in subjects with clinically significant laboratory abnormalities noted at or after the TOC Visit.
[m]Collect blood for acute (Screening) and convalescent (TOC) atypical pathogen serology, including *M. pneumoniae*, *C. pneumoniae* and *L. pneumophilia*, for central laboratory testing.
[n]At Screening, a urine dipstick will be performed locally; if results are abnormal and deemed clinically significant by the Investigator, a urinalysis will be sent to the central laboratory.
[o]At Screening, urine will be collected for central laboratory testing of *S. pneumoniae* and *L. pneumophilia*. These tests will be done by the central laboratory using rapid antigen test kits
[p]At Screening, a local laboratory urine or serum pregnancy test (females only) is required to confirm study eligibility. In addition, blood will be collected from all female subjects for serum β-human chorionic gonadotropin pregnancy test by the central laboratory at the Screening and TOC visits.
[q]At Screening, the collection of expectorated sputum or other deep respiratory sample should be attempted in all subjects. Gram staining will be performed on all sputum specimens and quality will be assessed. Culture will be performed on all sputum samples of adequate quality or deep respiratory specimens. For subjects being enrolled at Indian sites, an expectorated sputum sample will be collected for diagnostic evaluation of tuberculosis by Xpert TB test (using GeneXpert) in addition to collection of expectorated sputum or other deep respiratory sample for microbiological assessments as per protocol (section 16.1). Collection of two sputum/respiratory samples, on the day of screening, could be scheduled at the discretion of investigator based on convenience of the subject. Post-baseline respiratory specimens should be collected as clinically indicated and from subjects who are clinical failures and require alternative antibacterial treatment for CABP.
[r]Two sets of blood cultures (each set consists of 1 aerobic and 1 anaerobic blood culture bottle) will also be collected at Screening. If baseline blood cultures are positive, repeated post-baseline blood cultures should be collected until a negative result is obtained. Sites may wait until confirmation of results of previous cultures to collect further samples.
[s]Blood samples for PK analysis will be collected from all subjects at sites where PK sampling is possible on Day 1, Day 3 and Day 4, including within 10 min before study drug administration on Day 3 and after dosing at 2-4 h (Day 1 and Day 3) and 24-28 h (On Day 4, i.e. 24-28 h after Day 3 dose). Subjects who have been hospitalised are also required to have a post-dose PK sample at 6-10 h (Day 1 and Day 3).
[t]Study drug should be administered q24h (± 4 h) between Day 1 and EOT, with the exception of Day 2 in which an additional window of 4 h may be utilised, depending on the randomisation time on Day 1.
[u]AEs and SAEs will be recorded and reported from signing of the informed consent to the FU Visit.

[v]Prior medications that have been administered within 14 days before the date of signing the informed consent or during the Screening Phase will be recorded in the electronic Case Report Form (eCRF). All medications administered after the first dose of the study drug must be recorded in the eCRF.
[w]Best possible efforts should be made to conduct an in-person visit. In the event that an in-person visit is not possible due to any reason, assessment may be conducted through a telephone contact.

9. SAMPLE SIZE

This study is designed to demonstrate non-inferiority of oral nafithromycin (800 mg once daily) compared with oral moxifloxacin (400 mg once daily).

Nafithromycin will be declared non-inferior to moxifloxacin if the lower limit of the 2-sided 95% confidence interval (CI) for the difference between treatment groups (nafithromycin minus moxifloxacin) in the proportion of subjects with a favourable clinical response at Day 4 in the MITT analyses set is greater than -0.125.

In these comparisons, the margin of 12.5% has been determined based on historic data regarding the treatment effect of antibiotics.

For the determination of the study sample size, favourable clinical response rates at Day 4, obtained from studies of subjects with CABP, of 85% to 90% are projected, with an anticipated dropout rate by Day 4 of at most 5%. However, since subjects who dropout from the study prior to Day 4 will be included in the denominator for the calculation of the proportion of subjects with favourable clinical response at Day 4, an attained favourable response rate at Day 4 of 81% to 86% is anticipated in each treatment group. Based on these attained favourable clinical response rates at Day 4, a non-inferiority margin of 12.5%, and using the Farrington-Manning sample size approach for the Miettinen and Nurminen method, it has been determined that approximately 414 adult subjects (207/arm) will need to be included in the MITT analyses set in order for the statistical test to have a-priori at least 90% power at the 1-sided 2.5% significance level when the attained favourable response rate is at least 81%. Given the number of subjects needed in the MITT (N=414) analyses set and assuming a TB rate of 15%, 488 subjects will need to be enrolled into the study.

10. SAMPLE SIZE RE-ESTIMATION

Sample size re-estimation shall be done if the percentage of subjects who tested positive for MTBC is higher than anticipated, the target number of enrolled subjects may be increased to ensure the study is sufficiently powered for the MITT analyses set.

11. GENERAL STATISTICAL METHODOLOGY

Baseline for efficacy or safety analysis is defined as the last non-missing efficacy or safety assessment before the first dose of study drug in the Double-blind Treatment Period.

In general, descriptive statistics: n, mean, standard deviation, median, minimum, and maximum, for continuous variables and number and percentage of subjects in each category for categorical variables will be provided by treatment group for all the variables.

All source data will be presented as subject data listings.

12. ANALYSES SETS

12.1 ALL ENROLLED SUBJECTS

For the purpose of this SAP, subjects who signed informed consent and meet eligibility criteria (except detection of mycobacterium tuberculosis complex (MTBC)), irrespective of whether they were randomized or not. Subjects for whom MTBC is detected are included in this analyses set.

12.2 INTENT-TO-TREAT (ITT) ANALYSES SET

The ITT analysis set will include all subjects who were randomised, regardless of whether the subject actually received the study drug.

12.3 MODIFIED INTENT TO TREAT (MITT) ANALYSES SET

All ITT subjects who received at least one dose of study drug and these subjects will be analysed as per the randomized treatment groups. Subjects with detection of MTBC (as per results of GeneXpert TB test) indicative of active pulmonary tuberculosis will be excluded from this analyses set.

12.4 SAFETY ANALYSES SET

The safety analyses set will include all subjects who receive any amount of the study drug. Subjects will be analysed according to the treatment actually received.

12.5 EXPANDED MICROBIOLOGICAL MODIFIED INTENT-TO-TREAT (Expanded mMITT Analyses set) ANALYSES SET

This will be identical to the mMITT (defined in the next section) except that Subjects with sole baseline Gram-negative bacterial infection with bacteria from the *Enterobacteriaceae, Pseudomonadaceae* or *Yersiniaceae* are not excluded.

12.6 MICROBIOLOGICAL MODIFIED INTENT-TO-TREAT (mMITT) ANALYSES SET

All MITT subjects who have received at least 1 dose of study drug and have at least 1 baseline pathogen known to cause CABP against which the investigational drug has antibacterial activity, including bacterial pathogens identified by respiratory specimen culture, blood culture, and/or urinary antigen test (e.g. *S. pneumoniae*, *S. aureus, H. influenzae, H. parainfluenzae, M. catarrhalis, L. pneumophilia*) or atypical bacterial serologic response (*M. pneumoniae*, *C. pneumoniae*, *L. pneumophilia*) will be included in this analyses set. Subjects with sole baseline Gram-negative bacterial infection with bacteria from the *Enterobacteriaceae*, *Pseudomonadaceae* or *Yersiniaceae* will be excluded from this analyses set.

12.7 CLINICALLY EVALUABLE (CE) ANALYSES SET

The CE analyses set will include all MITT subjects who follow important components of the trial. To be included in the CE analyses set, subjects must meet all of the following criteria:

- Meet key Inclusion Criteria, including the clinical disease criteria for CABP (Inclusion Criteria #3a, #3b, #3c, #3d and #3e in the protocol)
- Do not meet key Exclusion Criteria (#1 through # 6, #16 and #18 in the protocol)
- The TOC Visit occurred within a window of 11 to 21 days. Days from the date of randomisation unless the subject was deemed a clinical failure before this visit. Note that for the purpose of this SAP, the window has been widened from 11 to 19 days to 11 to 21 days in order to allow inclusion of subjects whose visit to the clinic was delayed for social reasons
- Do not receive non-study, potentially effective against the baseline pathogen(s), systemic antibacterial therapy between Day 1 and the assessment TOC
- Do not have a clinical outcome of Indeterminate at the TOC Visit
- Receive at least 80% of the intended doses of randomised study drug therapy (based on number of tablets dispensed/received)
- Receive at least 48 h of study drug therapy to be considered an evaluable clinical failure and at least 72 h of study drug therapy to be considered an evaluable clinical success
- Do not have any other significant protocol violation(s) that may confound efficacy assessments at TOC

12.8 PHARMACOKINETIC (PK) ANALYSIS SET

The PK analysis set includes all subjects in the safety who received at least 1 dose of nafithromycin and had at least 1 analyzable plasma PK sample.

13. DISPOSITION

A clear accounting of the disposition of all subjects who enter the study will be provided, from screening to study completion.

Number and percentage of subjects who were screened, screen failed (including reasons for screen failures) will be summarized. For Enrolled subjects, the number randomised, completed study drug, completed through Day 4, EOT and TOC, completed the study and reasons for discontinuations of study drug as well as discontinuation from study will be summarized by treatment group and overall. This will be repeated using the MITT analyses set.

The number and percentage of subjects enrolled at each investigational site will also be presented by treatment and overall for all enrolled subjects.

Number and percentage of subjects in each analyses set, including reasons for exclusions, will also be presented by treatment and overall for all enrolled subjects.

The number of subjects with MTBC detected will be summarized by treatment group in the ITT analyses set.

14. PROTOCOL DEVIATION

Protocol deviations (PDs) will be classified as significant (S) or non-significant (NS). All deviations will be listed. Significant PDs will be summarized by treatment group in the ITT and MITT analyses set.

The list of categories for the PD is shown below along with the abbreviated description to be used in summaries and listings.

Table 1. Definitions of Significant and Non-Significant Protocol Deviations

No.	Deviation Description	Abbreviated Description
Significant Deviations		
1	Subject was included in the study who did not meet eligibility criteria	Eligibility Criteria not met
2	Any randomization to the wrong stratum	Randomization within incorrect PORT risk class
3	Subject who received study medication other than the one to which she/he was assigned	Treatment opposite randomized assignment
4	Subject who was dosed incorrectly, had large departures from dosing schedule or was otherwise non-compliant with study drug	Incorrect dosing or non-compliance with study drug administration
5	Subject who was dosed more than 7 days	Received > 7 days of study drug
6	Subject who received a non-study systemic antibacterial agent with potential activity against CABP pathogens	Received prohibited systemic antimicrobial
7	Subject who received another prohibited medication as defined in the protocol	Received other prohibited medications (non-antimicrobial)
8	PDs that impact efficacy assessments and are directly related to the study endpoints such as delayed or missed or assessments done outside of the protocol-defined windows at Day 4, EOT and TOC visits, deviations on the IP management and administration.	Missed, incomplete or non-adherence to efficacy assessments
9	Deviations relating to informed consent document administration. (e.g. Study specific procedure performed prior to obtaining Informed Consent, Subject signed incorrect ICF version, and subject did not sign the most recent version of the ICF at	Informed consent deviation

	the next available visit.)	
Non-Significant Deviations		
1	AE reporting (specifically PDs that do not compromise the safety of the subjects)	Missed AE report where safety was not compromised
2	Study procedures not impacting the evaluation of efficacy or safety of the study participants (e.g. Any assessment/laboratory procedures not conducted as per protocol, or not required for reasons relevant to patient safety such as samples sent for central laboratory analysis for FU Day 31 although there was no clinically significant laboratory abnormalities noted at or after the TOC Visit- as mentioned in the time).	Procedure not impacting efficacy or safety assessment not done
3	Investigational product (includes minor occasional deviations from the scheduled times of administration that is q24h (± 4 h) between Day 1 and EOT, with the exception of Day 2 in which an additional window of 4 h may be utilised, depending on the randomisation time on Day 1).	Minor deviations from dosing schedule

Note that criteria for the CE analyses set are a subset of the protocol deviations listed above. In the protocol these were referred to as ‘violations’.

15. BASELINE CHARACTERISTICS

15.1 Demographics and Baseline Characteristics

Demographics and baseline characteristics such as gender, age, race, ethnicity, weight, and body mass index (BMI), PORT scores, relevant medical history, and clinical signs and symptoms will be summarized by treatment group for the MITT and Safety analyses set.

Vital signs at baseline will be summarized in the MITT analyses set.

15.2 Medical History, Co-Morbid Conditions and Markers of CABP

Medical/Surgical history will be summarized for the Safety analyses set by treatment group and overall. Descriptive summaries of Co-morbid conditions, Markers of CABP and vital signs will be generated by treatment group in the MITT analyses set. See mock shells for the specific list of parameters included.

15.3 Gram Stain and Microbiological Results at Baseline

Gram stain results of (induced and/or expectorated) sputum samples as well as BAL and thoracentesis (if present) at baseline will be summarized using frequency and percentage by treatment group for the MITT analyses set.

Identification of typical pathogenic organisms at baseline from respiratory or blood specimens or urinary antigen tests will be summarized by the treatment group for the expanded mMITT and mMITT analyses set.

Pathogenic organisms identified at baseline from serology testing and urinary antigen tests will be summarized by treatment group for the expanded mMITT and the mMITT analysis set.

Infections by Gram type and number of pathogens isolated at baseline from respiratory or blood specimens, serology or urinary antigen tests will be summarized by treatment group for expanded mMITT and mMITT analyses set. The number of subjects with infections due to: monomicrobial Gram-positive, monomicrobial Gram-negative (including subjects with fastidious, or non-fastidious organisms), monomicrobial any Gram-positive or negative, monomicrobial atypical, all monomicrobial, polymicrobial all, polymicrobial Gram-positive, polymicrobial Gram-negative, polymicrobial typical pathogens only, (with the same Gram type), polymicrobial typical and atypical, where the subject has a single typical organism or the same Gram type, if more than one typical), mixed Gram-negative and Gram-positive: overall as well without atypical pathogens will be tabulated. A tabulation of the number of pathogens (1, 2, ≥3) identified per subject will be included.

Descriptive statistics of minimum inhibitory concentration (MIC) results at baseline of nafithromycin and also of moxifloxacin in the mMITT analyses set will be provided including the number isolated and number tested, minimum, maximum, MIC_{50} and MIC_{90}. Here MIC_{50} and MIC_{90} are the 50th and the 90th percentile values of MIC obtained rounded to the nearest whole value.

16. PRIOR AND CONCOMITANT MEDICATIONS, NON-DRUG THERAPIES AND PROCEDURES

Prior medications are defined as medications taken prior to the first dose of study drug. Concomitant medications are defined as medications taken at the time of first dose or after the first dose of study drug is administered. All medications as documented by the investigator will be coded using the Anatomical Therapeutical Chemical (ATC) code making use of the World Health Organization drug dictionary (WHODD) version 01 SEP 2021 and the Preferred name (PN).

Prior and concomitant medications (other than systemic antibiotics) will be summarized separately by treatment group, ATC and PN as well as overall by ATC and PN class in the Safety analyses set. If medications are taken continuously before and after the randomization visit then that medications will be included in both prior and concomitant medication summaries.

Prior and concomitant systemic antibiotics will be summarized separately by treatment, ATC and PN as well as overall by ATC and PN in the MITT analyses set. Also, a summary of systemic antibiotics within 72 hours prior to randomization will be provided.

For the summary tables, if a subject has taken a prior or concomitant medication more than once, the subject will be counted only once for the medication.

All reported medications will be included in the listing.

Similarly, Non-drug Therapies and Concomitant procedures will be summarized by Treatment Group in the Safety analyses set. Non-drug Therapies are coded using WHO Drug dictionary version 01 SEP 2021 and Concomitant procedures are coded using the MedDRA dictionary (Version 25.1).

17. TREATMENT EXPOSURE

Duration of therapy will be calculated as the last dose date/time of therapy minus the first dose date/time of therapy +1. Summary tables will include descriptive statistics as well as the number and percentage of subjects in each treatment group with duration of therapy in the following categories: ≤ 2, 2 < to 3, 3< to 4, 4< to 5, 5< to 6, 6 < to 7 and > 7 days.

The compliance rate to therapy will be calculated as the total number of doses received divided by the total number of doses expected then multiplied by 100. The total number of expected doses is the number of medication days multiplied by the number of expected doses per day. Number of medication days is the total number of days from the date/time of the first dose of study drug to the date/time of the last dose of study drug. If a subject receives ≤ 48 hours of therapy, the expected doses will be calculated assuming the subject received full 48 hours therapy. Although receipt of >7 days of therapy would be a protocol deviation, if a subject receives >7 days therapy, the expected doses will be calculated assuming the subject received 7 days therapy.

Percent compliance to therapy will be calculated using the following formula:

$$\%\text{compliance} = \frac{\text{no. of doses received} * 100}{\text{expected doses per day} * \text{total number of medication days}}$$

The number and percentage of subjects with % compliance: <80%, 80-120% and > 120% will be tabulated in each treatment group.

All summaries will be provided for the Safety and mMITT population.

18. STUDY EFFICACY VARIABLES / ENDPOINTS

18.1 Primary Efficacy Endpoints

Clinical Response at Day 4 (MITT Analyses set)

- Favourable Clinical Response: Alive and improvement of at least 1 level (e.g., severe to moderate, moderate to mild, mild to absent) in at least 2 CABP symptoms (dyspnoea, cough, production of purulent sputum or pleuritic chest pain) compared with that at the Baseline Visit, without worsening in any other of the 4 CABP symptoms. Severity of symptoms is based on a 4-point scale (absent, mild, moderate or severe).
- Unfavourable Clinical Response: No improvement of at least 1 level in at least 2 CABP symptoms compared with that at baseline; or worsening in any of the 4 CABP symptoms compared with that at baseline; or death from any cause at or before Day 4.
- Indeterminate: Study data are missing for evaluation of efficacy at Day 4 for any reason, including loss to FU.

18.2 Secondary Efficacy Endpoints

The secondary analysis variables are listed below

- Clinical response at Day 4 (mMITT and CE analyses sets)
- Investigator determined Clinical outcome at EOT (MITT, mMITT and CE analyses sets)
- Investigator determined Clinical outcome at TOC (MITT, mMITT and CE analyses sets)
- Hospitalisation prior to FU (MITT analyses set)
- By-pathogen clinical response at Day 4 and by-pathogen clinical outcome at TOC (mMITT analyses sets)

Investigator determined Clinical Outcome at EOT (MITT, mMITT and CE Analyses Sets)

- Clinical Cure: Alive and signs and symptoms of CABP (dyspnoea, production of purulent sputum or pleuritic chest pain) are resolved or return to premorbid conditions and cough is improved such that further antibacterial therapy is no needed and the subject otherwise cannot be declared a clinical failure.
- Clinical Failure: The signs and symptoms of CABP (dyspnoea, production of purulent sputum or pleuritic chest pain) did not resolve or return to premorbid and/or cough worsened, such that non-study antibacterial therapy must be initiated for the treatment of CABP or death occurred prior to assessment or subject received alternative anti-microbial therapy for the treatment of CABP prior to this visit.

- Indeterminate: Study data are missing for the evaluation of efficacy at the assessment visit for any reason, including loss to FU.

Investigator determined Clinical Outcome at TOC (MITT, mMITT and CE Analyses Sets)

- Clinical Cure, Clinical Failure or Indeterminate: Definitions are as above. Failures at EOT will be carried forward to TOC.

Hospitalisation Prior to FU (MITT Analyses set)

- Hospital re-admission for any reason between the Day 1 and the FU visits, if previously hospitalised and discharged or initial hospital admission for any reason between the Day 2 and the FU visits (Day 31 ± 4 days), if not previously hospitalised on Day 1.

By-Pathogen Clinical Response at Day 4 and By-Pathogen Clinical Outcome at TOC in the mMITT Analysis Set

The by-subject clinical response at Day 4 and clinical outcome at TOC defined above will be applied to each pathogen to obtain the:

- By-pathogen favorable Response at Day 4 (definitions as mentioned above)
- By-pathogen cure rate at TOC: (definitions as mentioned above)

18.3 Safety Endpoints

- Incidence of treatment-emergent adverse events (TEAEs), serious adverse events (SAEs) and discontinuations due to TEAEs
- Incidence of potentially clinically significant (PCS) changes in safety laboratory parameters, vital signs and electrocardiograms (ECGs)

19. EFFICACY ANALYSES

Except for the safety analyses set, subject data will be analysed in the treatment group to which the subject was randomised. For the safety analyses set, subject data will be analysed according to the treatment group that the subject actually received.

19.1 Primary Efficacy Analysis

The primary efficacy endpoint for this study is the proportion of subjects with favourable clinical response at Day 4 in the MITT analyses set. Subjects will be categorised as having Favourable, Unfavourable or Indeterminate response. Subjects with missing data or who are lost to follow-up (FU) are defined as Indeterminate and will be included in the denominator for the calculation of the proportion of subjects with Favourable clinical response at Day 4.

The number and percentage of subjects in each treatment group and in each response category will be reported.

The null (H_0) and alternative (H_1) hypotheses are the following:

H_0: $\pi_1 - \pi_2 \leq -0.125$ vs. H_1: $\pi_1 - \pi_2 > -0.125$

Where:

π_1 = the proportion of subjects with favourable clinical response at Day 4 in the nafithromycin treatment group

π_2 = the proportion of subjects with favourable clinical response at Day 4 in the moxifloxacin treatment group

The non-inferiority hypothesis test is a 1-sided hypothesis test performed at the 2.5% level of significance. The statistical test is based on the lower limit of the 2-sided 95% CIs for the observed difference in the proportion of subjects with favourable clinical response at Day 4 (nafithromycin minus moxifloxacin). The CI will be obtained using the method of Miettinen and Nurminen.

If the lower limit of the 2-sided 95% CI for the difference between treatment groups (nafithromycin minus moxifloxacin) in the proportion of subjects with favourable clinical response rates at Day 4 in the MITT analyses set is greater than –0.125, the null hypothesis H0 will be rejected in favour of the alternative hypothesis H1 and the non-inferiority of nafithromycin monotherapy to moxifloxacin will be concluded. In addition, if the lower limit of the 2-sided 95% CI for the difference between treatment groups (nafithromycin minus moxifloxacin) in the proportion of subjects with favourable clinical response rates at Day 4 is greater than zero, the superiority of nafithromycin monotherapy to moxifloxacin monotherapy will also be concluded.

19.2 Sensitivity Analysis

The following sensitivity analyses for the primary efficacy outcome will be conducted:

1. Comparison of the clinical response rates at Day 4 between treatment groups in the MITT analyses set using the Miettinen-Nurminen statistic stratified by the randomisation factor: PORT Risk Class: I (ineligible for study), II (eligible), III (eligible), IV (score of 91-105 eligible and 106-130 ineligible) and V (ineligible for study). Stratum classes for PORT score are II and III/IV.
2. Subjects with missing data will be considered to have an Indeterminate response. By definition, subjects with indeterminate response will be included in the denominator for analyses in the MITT analyses set effectively counting these as unfavorable responses. A sensitivity analyses in which subjects with Indeterminate outcome in the MITT analyses set are imputed as having a favourable response will also be conducted using the same statistical methods as for the primary analysis.

19.3 Secondary Efficacy Analysis

The secondary analyses variables are listed in section 17.2. For each by-subject secondary efficacy outcome, the number and percentage of subjects with each response category (e.g., for the clinical outcome at EOT response is Cure, Failure or Indeterminate) will be summarized by treatment group.

The 2-sided 95% CI for the difference between treatment groups in the proportion of subjects with favourable response (e.g., for the clinical outcome at EOT: the difference between treatment groups in proportions of subjects with clinical cure at EOT) will be presented using the same approach as for the primary analyses. These analyses will be done for the outcomes/visits/analyses set combinations shown below (Table 2).

Table 2. Secondary Analysis Variables by Visit and Analyses Set.

Analysis Variable	Visit	Analyses set		
		MITT	mMITT	CE (at TOC)
**Clinical Response**	Day 4	NA (primary)	X	X
**Investigator-determined Clinical Outcome**	EOT	X	X	X
	TOC	X	X	X
**Hospital re-admission Rates**	Prior to FU	X		

Abbreviations: CE = Clinically Evaluable; MITT = Modified Intent-to-Treat; mMITT = Microbiological Modified Intent-to-Treat; TOC = Test of Cure

Similar tabulations will be done for the by-subject favourable clinical response at Day 4 and the by-subject investigator-determined clinical cure rate at TOC in subjects with bacteremia and in subjects with at least one multi-drug resistant (MDR) pathogen, defined as resistant to 2 or more antibiotics belonging to different classes (e.g.. macrolides, beta-lactams, fluoroquinolones and tetracyclines) using both the US Food and Drug Administration Susceptibility Test Interpretive Criteria (FDA STIC) and the European Committee on Antimicrobial Susceptibility Testing (EUCAST) interpretive criteria and

identified from an appropriately collected respiratory specimen or blood in the mMITT analysis set, except that CIs will not be included if less than 10 subjects per treatment group are obtained.

The clinical response at day 4 as well as the investigator-determined clinical outcome at TOC will be summarized for the following subgroups in the mMITT analyses set:

- Subjects with any monomicrobial infection
- Subjects with monomicrobial infection caused by Gram-negative pathogens
- Subjects with monomicrobial infection caused by fastidious Gram-negative pathogens
- Subjects with monomicrobial infections caused by non-fastidious Gram-negative pathogens
- Subjects with monomicrobial infections caused by Gram-positive pathogens
- Subjects with monomicrobial infections caused by atypical pathogens
- Subjects with any polymicrobial infections [Note: subjects with both typical (single typical or of the same Gram type is > 1) and atypical pathogen are included here]
- Subjects with polymicrobial infections due to Gram-positive pathogens only
- Subjects with polymicrobial infections due to Gram-negative pathogens only
- Subjects with polymicrobial infections due to atypical pathogens only
- Subjects with polymicrobial infections due to typical pathogens only of the same Gram type
- Subjects with mixed Gram-positive and Gram-negative Infections
- Subjects with mixed Gram-positive and Gram-negative Infections without atypical pathogens

The by-pathogen favourable clinical response at Day 4 and also the by-pathogen clinical cure rate at TOC will be summarized in the mMITT analyses set.

In the by-pathogen summaries, typical pathogens will be categorized as Gram-negative-fastidious, Gram-negative non-fastidious and Gram-positive.

The by-pathogen favourable clinical response at Day 4 and also the by-pathogen cure rate at TOC will also be summarized in the mMITT analyses set for:

- Subjects with monomicrobial infections due to typical pathogens
- Subjects with monomicrobial infections due to atypical pathogens
- Subjects with polymicrobial infections
    - Subjects with polymicrobial infections due to typical pathogens only
    - Subjects with polymicrobial infections due to typical and atypical

pathogens

- Subjects with polymicrobial infections due to atypical pathogens only

The incidence of superinfections and new infections will be summarized and listings by pathogen will be provided for each treatment group. In addition, subjects in the mMITT analyses set, whose baseline pathogen(s) has a decreased susceptibility (defined as a 4-fold increase in the baseline MIC) to the study treatment received in a post-baseline culture will be listed.

An assessment of hospitalisation (defined as re-admission to the hospital if previously hospitalised on or after study drug initiation [Day 1] and before FU or initial hospital admission [if not previously hospitalised] for any reason between the initiation of the study drug (Day 1) and before FU will be summarized using frequency and percentage for subjects in MITT analyses set.

Descriptive summaries of the changes from baseline/return to pre-morbid at each study visit will be generated for each individual CABP symptom: dyspnoea, cough, production of purulent sputum and pleuritic chest pain. For visits prior to the EOT visit a favourable symptom outcome will be defined as an improvement from baseline in the given symptom. For the EOT and TOC visits, Favourable symptom outcome will be defined as complete resolution or return to pre-morbid of the baseline symptom. Since not all symptoms may have been present at baseline, symptoms with No change will also be included.

19.4 Subgroup Analysis

Subgroup analyses for the Favourable clinical Response at Day 4 in the MITT analyses set will be presented. Subgroups are as follows:

Demographic and Baseline Characteristics

- Age groups (Group 1: <50 years, ≥50 years, Group 2: <65 years, ≥65 years, Group 3: 65 to 75 years, Group 4: <75years, ≥75 years)
- Gender
- Race
- BMI (kg/m^2) categories (< 18.5 (underweight), 18.5 to < 25 (normal), ≥ 25 to < 30 (overweight), ≥ 30 to < 35 (obese) or >= 35 (morbidly obese))

Markers for CABP- Clinical Parameters

- PORT Risk Class (II, III/IV)
- Subjects with and without prior systemic antibiotic usage within 72 hours of randomization.
- Subjects with and without presence of SIRS (Systemic Inflammatory Response Syndrome) defined as the presence of at least 2 of: Body temperature >38°C or < 36°C, heart rate > 90 beats/min, respiratory rate > 20 breaths/min, WBC < 4,000 cells/mm³ or > 12,000 cells/mm³ or the presence of >10% immature neutrophils

(band forms)

- Fever (Yes and No) (tympanic temperature > 38.0°C [100.4°F] or < 36.0°C [95.5°F])
- Auscultatory findings (Absent/Present)
- Presence of bacteremia at baseline (Yes and No)
- Presence of hypoxia (Yes/No) (PaO_2< 60 mmHg or O_2 saturation < 90%)
- Pleural Effusion (No, unilateral, bilateral)
- CABP involvement: Unilateral and Unilobar, Unilateral and multi-lobar, Bilateral

Co-Morbid Conditions

- Tuberculosis (Never, Past History, Active Tuberculosis)
- COPD (Y/N)
- Prior pneumonia (Y/N)
- History of cancer in the lungs (Never/Past History)
- Smoking History (Never, Past, Current)
- Baseline Creatinine clearance (mL/min) group (< 30, ≥ 30 to ≤ 60, > 60 to ≤ 90 or > 90). Baseline Creatinine clearance values obtained from the central laboratory will be used. For subjects without a value obtained from the central laboratory, the baseline value from the local laboratory will be used, if available.

20. SAFETY ANALYSES

Safety data will be presented based on the Safety Analyses set.

Safety will be evaluated by presenting summaries of AEs, vital signs, laboratory evaluations (hematology, chemistry panel, urine analysis) and ECG parameters.

For each safety parameter, unless otherwise stated, the last assessment made before the first administration of the study drug will be used as the baseline value.

20.1 Adverse Events

A treatment-emergent adverse event (TEAE) is defined as an adverse event that occurs during or after the first administration of study drug and up through the FU visit or a pre-existing AE that worsens in severity after study drug administration.

All TEAEs will be classified by using the MedDRA (Version 25.1) System organ class (SOC) and preferred term (PT)

The number and percentage of subjects who experienced at least one TEAE will be summarized by treatment group, SOC and PT. Although a subject may have two or more TEAEs, at each level of summarization, the subject is counted only once. The same subject may contribute to two or more PTs in the same System Organ Class category.

In TEAE summaries, the primary system organ class will be presented alphabetically and the preferred terms will be sorted within primary SOC alphabetically.

The following adverse event summary tables will be produced by treatment and overall:

- Overall Summary
- TEAE by SOC and PT
- Treatment Emergent SAE by SOC and PT
- TEAE by SOC and PT summarized by Severity (for multiple occurrences of the same TEAE, the worse severity (mild. moderate or severe) will be used)
- TEAE by SOC and PT summarized by Relationship (Related, not related. For multiple occurrences of the same TEAE, the most related one will be used)

TEAEs and drug-related TEAEs will also be summarized by treatment group, SOC and PT for the subgroups defined by: PORT risk class, Gender (Male or Female), Age categories (<50 years, ≥50 years, <65 years, ≥ 65 years; 65 to 75 years; < 75 years, ≥ 75 years), BMI (kg/m^2) categories (< 18.5 (underweight), 18.5 to < 25 (normal), ≥ 25 to < 30 (overweight), ≥ 30 to < 35 (obese) or >= 35 (morbidly obese)), and Baseline Creatinine Clearance (mL/min) group (< 30, ≥ 30 to ≤ 60, > 60 to ≤ 90, or > 90).

All safety data, including physical examination and urinalysis results (as available), will also be provided in by-subject listings.

20.2 Clinical Laboratory Evaluations

Descriptive statistics for mean and mean changes in clinical laboratory tests (hematology, chemistry, and coagulation) will be presented by study visit for each treatment group.

Potentially clinically significant (PCS) clinical laboratory results will be determined based on normal limits and the worst post-baseline value using the definitions shown below for each test. Summaries of the number and percentage (%) of subjects meeting PCS criteria will be generated using the subgroup of subjects in the safety analyses set that have both a baseline and any post-baseline laboratory evaluation.

Shift tables will be presented to show the number and percentage of subjects with a laboratory value below the lower limit of normal (LLN), within normal limits, above the upper limit of normal (ULN) and missing at baseline versus the value at each visit and the worst post-baseline value. Percentages for each laboratory test will be based on the number of subjects in the Safety analyses set.

Table 3: Potential Clinical Significance (PCS) for Hematology Parameters

Laboratory Parameter	*Flag*	*Criteria**	
		Observed Value	*Change from Baseline*
Hemoglobin (g/L)	High (CH)	>1.3 × ULN	>30% Increase from Baseline
	Low (CL)	<0.8 × LLN	>20% Decrease from Baseline
Hematocrit (%)	High (CH)	>1.3 × ULN	>30% Increase from Baseline
	Low (CL)	<0.8 × LLN	>20% Decrease from Baseline
Erythrocyte count (RBC) (10^12/L)	High (CH)	>1.3 × ULN	>30% Increase from Baseline
	Low (CL)	<0.8 × LLN	>20% Decrease from Baseline
Mean corpuscular Volume (MCV) (fL)	High (CH)	>1.15 × ULN	>15% Increase from Baseline
	Low (CL)	<0.85 × LLN	>15% Decrease from Baseline
Mean corpuscular hemoglobin (MCH) (pg)	High (CH)	>1.15 × ULN	>15% Increase from Baseline
	Low (CL)	<0.85 × LLN	>15% Decrease from Baseline
Mean corpuscular hemoglobin concentration (MCHC)(g/L)	High (CH)	>1.15 × ULN	>15% Increase from Baseline
	Low (CL)	<0.85 × LLN	>15% Decrease from Baseline
Leukocyte count (WBC)(10^9/L)	High (CH)	>1.6 × ULN	>100% Increase from Baseline
	Low (CL)	<0.65 × LLN	>60% Decrease from Baseline
Neutrophils (%)	High (CH)	>1.6 × ULN	>100% Increase from Baseline
	Low (CL)	<0.65 × LLN	>75% Decrease from Baseline
Lymphocytes(%)	High (CH)	>1.5 × ULN	>100% Increase from Baseline
	Low (CL)	<0.25 × LLN	>75% Decrease from Baseline
Eosinophils(%)	High (CH)	>4.0 × ULN	>300% Increase from Baseline
	Low (CL)	N/A	N/A
Monocytes(%)	High (CH)	>4.0 × ULN	>300% Increase from Baseline
	Low (CL)	N/A	N/A
Basophils(%)	High (CH)	>4.0 × ULN	>300% Increase from Baseline
	Low (CL)	N/A	N/A
Platelets (10^9/L)	High (CH)	>1.5 × ULN	>100% Increase from Baseline
	Low (CL)	<0.65 × LLN	>50% Decrease from Baseline

Notes: PCS = potentially clinically significant, ULN= upper limit of normal, LLN= lower limit of normal
CH = high PCS based on criterion value and increase from baseline.
CL = low PCS based on criterion value and decrease from baseline.
N/A= Not Applicable
*A post-dose value is considered as a PCS value if it meets both criteria for observed value and change from baseline

Table 4: Potential Clinical Significance (PCS) for Serum Chemistry Parameters

Laboratory Parameter	*Flag*	*Criteria**	
		Observed Value	*Change from Baseline*
Bicarbonate (mmol/L)	High (CH)	>1.3 × ULN	>40% Increase from Baseline
	Low (CL)	<0.7 × LLN	>40% Decrease from Baseline
Sodium (mmol/L)	High (CH)	>1.1 × ULN	>10% Increase from Baseline
	Low (CL)	<0.85 × LLN	>10% Decrease from Baseline
Potassium (mmol/L)	High (CH)	>1.2 × ULN	>20% Increase from Baseline
	Low (CL)	<0.8 × LLN	>20% Decrease from Baseline
Magnesium(mmol/L)	High (CH)	>3.0 x ULN	>200% Increase from Baseline
	Low (CL)	<0.5xLLN	>65% Decrease from Baseline
Phosphorus (mmol/L)	High (CH)	>3.0 × ULN	>200% Increase from Baseline
	Low (CL)	<0.5 × LLN	>50% Decrease from Baseline
Chloride (mmol/L)	High (CH)	>1.2 × ULN	>20% Increase from Baseline
	Low (CL)	<0.8 × LLN	>20% Decrease from Baseline
Calcium (mmol/L)	High (CH)	>1.3 × ULN	>30% Increase from Baseline
	Low (CL)	<0.7 × LLN	>30% Decrease from Baseline
Alkaline phosphatase (ALP)(U/L)	High (CH)	>2.0 × ULN	>100% Increase from Baseline
	Low (CL)	<0.5 × LLN	>80% Decrease from Baseline
Alanine aminotransferase(ALT)(U/L)	High (CH)	>3.0 × ULN	>200% Increase from Baseline
	Low (CL)	N/A	N/A
Aspartate aminotransferase(AST) (U/L)	High (CH)	>3.0 × ULN	>200% Increase from Baseline
	Low (CL)	N/A	N/A
Gamma-glutamyltransferase(U/L)	High (CH)	>3.0 × ULN	>200% Increase from Baseline
	Low (CL)	N/A	N/A
Lactatede hydrogenase (LDH)(U/L)	High (CH)	>4.0 × ULN	>300% Increase from Baseline
	Low (CL)	< 0.4 × LLN	>60% Decrease from Baseline
Bilirubin, Total (umol/L)	High (CH)	>2.5 × ULN	>150% Increase from Baseline
	Low (CL)	N/A	N/A
Bilirubin, Direct (umol/L)	High (CH)	>2.5 × ULN	>150% Increase from Baseline
	Low (CL)	N/A	N/A
Glucose, non-fasting (mmol/L)	High (CH)	>3.0 × ULN	>200% Increase from Baseline
	Low (CL)	<0.6 × LLN	>40% Decrease from Baseline
Protein, Total (g/L)	High (CH)	>1.5 × ULN	>50% Increase from Baseline
	Low (CL)	<0.5 × LLN	>50% Decrease from Baseline
Albumin (g/L)	High (CH)	>1.5 × ULN	>50% Increase from Baseline
	Low (CL)	<0.5 × LLN	>50% Decrease from Baseline
Serum creatinine (umol/L)	High (CH)	>2.0 × ULN	>100% Increase from Baseline

	Low (CL)	N/A	N/A
Blood Urea Nitrogen (BUN) (mmol/L)	High (CH)	>3.0 × ULN	>200% Increase from Baseline
	Low (CL)	N/A	N/A

Notes: PCS = potentially clinically significant, ULN= upper limit of normal, LLN= lower limit of normal
CH = high PCS based on criterion value and increase from baseline.
CL = low PCS based on criterion value and decrease from baseline.
N/A= Not Applicable
*A post-dose value is considered as a PCS value if it meets both criteria for observed value and change from baseline.

For the calculated creatinine clearance, shifts from baseline to lowest post-baseline value will be presented using the cut-offs: < 30, 30-60, > 60-90, > 90 (mL/min), missing. Within each cross-tabulation, the number and percent of subjects will be tabulated.

For subjects with normal liver parameters (on a per-analyte basis) at baseline, the number and percentage of subjects with the following liver chemistry parameters will be summarized:

- ALT ≥3×ULN, ≥5×ULN, ≥10×ULN
- AST ≥3×ULN, ≥5×ULN, ≥10×ULN,
- Total bilirubin > 1.5x ULN and >2×ULN
- Alkaline Phosphatase(ALP)≥1.5×ULN and ≥2×ULN
- ALT ≥3×ULN and Total bilirubin >1.5×ULN
- AST ≥3×ULN and Total bilirubin >2×ULN
- Potential Hy's Law cases: ALT or AST ≥3×ULN, Total bilirubin >2×ULN, and ALP ≤ 2×ULN (where each individual result does not need to occur in the same blood draw)

All clinical laboratory data will be listed. Values outside the normal ranges will be flagged.

The coagulation parameters: partial thromboplastin time (PTT), prothrombin time (PT) and International Normalized ratio (INR) was collected using local laboratories but the normal ranges were not obtained. The number and percentage of subjects with PCS changes in the international normalized ratio only will be presented by treatment group, based on the threshold in Table.

Table 5: Criteria for Potentially Clinically Significant (PCS) Coagulation Parameters

Coagulation Parameter	***Flag***	***Criteria****	
		Observed Value	***Change from Baseline***
International Normalized ratio (INR)	High (CH)	> 2.0	N/A
	Low(CL)	N/A	N/A

Notes: PCS = potentially clinically significant
CH = high PCS based on criterion value and increase from baseline (if present).]
N/A= Not Applicable

*A post-dose value is considered as a PCS value if it meets both criteria for observed value and change from baseline.

20.3 Vital Signs

Descriptive statistics for the mean and mean changes from baseline for the Systolic Blood Pressure, Diastolic blood pressure, Heart rate, Temperature, Respiratory rate, Oxygen Saturation, arterial PH and partial pressure of arterial oxygen at each study visit will be provided.

The number and percentage of subjects with PCS changes in vital signs (systolic and diastolic blood pressure and heart rate) will be presented by treatment group, based on the following thresholds:

Table 6: Criteria for Potentially Clinically Significant (PCS) Vital Signs

Vital Sign Parameter	*Flag*	*Criteria**	
		Observed Value	*Change from Baseline*
Systolic Blood Pressure (mmHg)	High (CH)	≥ 180 mmHg	Increase of ≥ 20 mmHg
	Low (CL)	≤ 90 mmHg	Decrease of ≥ 20 mmHg
Diastolic Blood Pressure (mmHg)	High (CH)	≥ 105 mmHg	Increase of ≥ 15 mmHg
	Low (CL)	≤ 50 mmHg	Decrease of ≥ 15 mmHg
Heart Rate (bpm)	High (CH)	≥ 120 bpm	Increase of ≥25%
	Low (CL)	≤ 50 bpm	Decrease of ≥25%
Respiratory Rate (bpm)	High (CH)	≥ 20	N/A
	Low (CL)	≤ 12	N/A

Notes: PCS = potentially clinically significant
CH = high PCS based on criterion value and increase from baseline.
CL = low PCS based on criterion value and decrease from baseline.
N/A= Not Applicable
mmHg = Millimeter mercury, bpm = Beat per minute.

*A post-dose value is considered as a PCS value if it meets both criteria for observed value and change from baseline.

A listing of all vital signs will be provided by subject.

20.4 Electrocardiogram (ECG)

The number and percentage of subjects with PCS changes in ECG will be presented by treatment group, based on the thresholds given below. Summaries of the number and

percentage (%) of subjects meeting PCS criteria will be generated using the subgroups of subjects in the safety analyses set that have both a baseline and any post-baseline ECG evaluation.

Table 7.Criteria for Potentially Clinically Significant Safety ECG Values.

ECG Parameter	Flag	PCS Criteria[a]	
		Criterion Value	Change from baseline or baseline Cut-off Value
Heart Rate (bpm)	High (CH)	>120	Increase of ≥ 25%
	Low (CL)	<50	Decrease of ≥ 25%
PR Interval (ms)	High (CH)	>200	Baseline <= 200 msec
	Low (CL)	< 120	Baseline >=120 msec
QRS Interval (ms)	High (CH)	>100	Baseline <=100
QT Interval (ms)	High (CH)	>500	Baseline ≤ 500 msec
QT Interval (ms)	High (CH)	>470	Baseline ≤ 470 msec
QTcF (ms)	High (CH)	>500	Baseline of ≤ 500 msec
QTcF (ms)	High (CH)	>500	Increase from baseline >60
QTcF (ms)	High (CH)	>480	Baseline ≤480 msec
QTcF (ms)	High (CH)		Increase from baseline >30
QTcF (ms)	High (CH)		Increase from baseline >60
QTcB (ms)	High (CH)	>500	Baseline≤ 500 msec

Notes: CH = high PCS based on criterion value and increase from baseline (or baseline cut off value).
CL = low PCS based on criterion value and decrease from baseline.
PCS = potentially clinically significant. bpm = Beat per minute. msec = Millisecond.
QTcF (based on RR i.e. QTcF= QT/(RR)**1/3)
[a]A post-dose value is considered as a PCS value if it meets both criteria for observed value and change from baseline.

All ECG measurements and the overall interpretation will be listed by subject.

21. PK ANALYSIS

All PK related summaries will be done separately and reported in the PK report.

22. MULTIPLE COMPARISONS

No multiple comparison adjustment shall be made in this study.

23. METHODS FOR HANDLING MISSING DATA

- For demographic and baseline characteristics, each variable shall be presented using all available data. Subjects with missing data shall be excluded only from analyses sets for which data are not available
- Handling of Missing/Partial Dates for Adverse Events:

Partial/Missing Start Date

Missing day - Impute the 1st of the month unless month is same as month of first dose of study drug then impute first dose date.

Missing day and month – Impute 1st January unless year is the same as first dose date then impute first dose date.

Partial Date

Missing day - Impute the last day of the month unless month is same as month of first dose of study drug then impute last dose date.

Missing day and month – Impute 31st December unless year is the same as first dose date then impute last dose date.

Note: For the purpose of displaying related events, events with relationship marked as “Missing” relationship will be considered as “Treatment Related”.

In case the AE end date is missing, the AE will be kept as ‘ongoing’. The duration of AE in the listing will be displayed as >xx days. Here xx days means the total duration in days from the date when the AE started till the end of the study.

Missing times, severity and causality for AEs will be queried for a value. No imputations will be made for missing times. AEs with a missing time will be considered treatment emergent if the date is on or after the first dose of the study drug, AEs with missing severity will be considered severe and AEs with a missing relationship to the study drug will be considered related to the study drug.

- For the primary efficacy outcome at Day 4, subjects with missing data will be considered Indeterminate response. By definition, subjects with Indeterminate response will be included in the denominator for analyses in the MITT analyses set.
- Missing values for other efficacy outcomes will be handled in a similar manner as the primary efficacy outcome.
- For evaluations at the TOC Visit, subjects with Indeterminate response will be included in the denominator for analyses in the MITT analyses set and will be excluded from the CE analyses set at TOC.

24. PLANNED ANALYSIS

24.1 Interim Analysis

There is no interim analysis planned for this study.

A blinded (aggregated across treatment groups) review of the percentage of subjects in the ITT analyses set who tested positive for MTBC (as per the results of the GeneXpert TB test) will be conducted when approximately 50% of subjects have been enrolled. If the percentage subjects who tested positive for MTBC is higher than anticipated, the target number of enrolled subjects may be increased to ensure the study is sufficiently powered for the MITT analyses set.

24.2 Final Analysis

The final analysis shall be conducted once all the subjects have completed the study, the data has been checked for quality and integrity and the database has been locked.

25. SOFTWARE USED FOR ANALYSIS

SAS Software, Version 9.4 (SAS Institute Inc., Cary, NC, USA) will be used for the randomisation, statistical analysis and Tables Listings and Figures (TLFs).

26. CHANGES FROM STUDY PROTOCOL

The changes listed below summarize differences between the protocol specified analyses and the statistical analysis plan.

1. Added the Enrolled analyses set as well as the Expanded-mMITT Analyses set in order to provide the broadest summarization of the data.
2. In the definition of the mMITT, the abbreviation ‘e.g.’ was inserted to clarify that the parenthetical list of acceptable organisms was not meant to be all inclusive
3. The allowed window for the TOC window in the CE analyses set was expanded from 11 to 19 days to 11 to 21 days in order to allow for inclusion of subjects whose visit was delayed for social reasons.

27. APPENDIX

1. Dictionary for Pathogens.
